# Supplementary material for: Vancomycin-Loaded, Nanohydroxyapatite-Based Scaffold for Osteomyelitis Treatment: In Vivo Rabbit Toxicological Tests and In Vivo Efficacy Tests in a Sheep Model
Source: Bioengineering (Basel). 2023 Feb 4;10(2):206. doi: 10.3390/bioengineering10020206 (PMC9952702; doi:10.3390/bioengineering10020206)
Supplement: Supplementary file 1 [file bioengineering-10-00206-s001.zip › bioengineering-2155793-supplementary.pdf]

MEDITOX

HECOLCAP
90-day Subchronic Toxicity Study after Intra-osseous Implantation in Rabbits

FINAL REPORT

GLP Study

Print No.:	1 of 3
Identification No.:	18/19/P
Study initiation date:	March 03, 2020
Final Report date:	November 03, 2020

CONTENT

		Page
**GLP COMPLIANCE STATEMENT**		**4**
**STATEMENT OF THE MANAGEMENT**		**5**
ABBREVIATIONS		6
**SUMMARY**		**8**
**1.**	**INTRODUCTION**	**10**
1.1.	Study Objective	10
1.2.	Project Staff	10
1.3.	Study Personnel	11
1.4.	Regulatory Guidelines	12
1.5.	GLP Compliance	12
1.6.	Facilities Management and Animal Husbandry	12
1.7.	Animal Welfare Act Compliance	12
**2.**	**TEST CONDITIONS**	**13**
2.1.	Experimental Design	13
2.2.	Description of Test Item	14
2.3.	Preparation of Test Item Formulation	15
2.4.	Test System	15
2.4.1.	Justification for Test System	15
2.4.2.	Allocation and Dosing	16
2.5.	Animal Selection, Randomization and Group Assignment	16
2.6.	Justification of Dose level and Route	16
2.7.	Housing	16
2.8.	Diet	16
2.9.	Water	16
2.10.	Acclimatization	16
2.11.	Animal Identification	17
**3.**	**METHODS**	**17**
3.1.	Implantation Procedure	17
3.2.	Anaesthesia	17
3.3.	Postoperative Care	18
3.4.	Clinical Observations and Mortality	18
3.5.	Body Weight	18
3.6.	Body Temperature	18
3.7.	Food Consumption	18
3.8.	Clinical Pathology	18
3.8.1.	Haematology and Clinical Chemistry	18
3.8.2.	Urinalysis	19
3.9.	Terminal Observation	20
3.9.1.	Necropsy	20
3.9.2.	Pathology Procedures	20
3.9.3.	Organ Weight	21
3.9.4.	Fixation	21
3.9.5.	Histotechnique	21
3.9.6.	Histopathology	21
3.10.	X-ray Examination	22
3.11.	Data Compilation	23
**4.**	**RESULTS**	**24**
4.1.	Clinical Observation and Mortality	24
4.1.1.	Detailed Clinical Observation	24
4.2.	Body Weight	24
4.3.	Body Temperature	24
4.4.	Food Consumption	24
4.5.	Clinical Pathology	25
4.5.1.	Haematology	25
4.5.2.	Clinical Chemistry	25
4.5.3.	Urinalysis	26
4.6.	Organ Weight Analysis	27
4.7.	Gross Pathology Examination and Histology	27
4.8.	X-ray Examination	29
**5.**	**CONCLUSION**	**29**

6. ARCHIVING **30**
7. AMENDMENTS AND DEVIATIONS **30**
8. DISTRIBUTION **31**

SUPPLEMENTS I **Number of Pages**

Table Set I	Body Weight	2
Graph Set I	Body Weight	1
Table Set II	Body Temperature	2
Graph Set II	Body Temperature	1
Table Set III	Food Consumption	3
Graph Set III	Food Consumption	1
Table Set IV	Haematology	5
Table Set V	Clinical Chemistry	6
Table Set VI	Urinalysis	1
Table Set VII	Organ Weight	5
Table Set VIII	Pathology	25

Statement of Quality Assurance Unit 1
GLP-certificate of the Test Facility 2

SUPPLEMENTS II

Photo-documentation 15
X-Ray photos 15

GLP COMPLIANCE STATEMENT

**Name of the Study:**	**HECOLCAP 90-day Subchronic Toxicity Study after Intra-osseous Implantation in Rabbits**
Identification No.:	18/19/P
Name of the Test Item:	HECOLCAP
Study initiation date:	March 03, 2020
Date of Interim Report:	August 17, 2020
Date of Draft Report:	October 12, 2020
Date of Final Report:	November 03, 2020

I the undersigned hereby declare that the objectives laid down in the Study Plan were achieved and as no untoward incidents occurred to adversely affect the quality or integrity of the study, I consider the data generated to be valid. This Final Report fully and accurately reflects the procedures used and data generated in the course of this study. This study was performed in compliance the OECD Principles of Good Laboratory Practice C (97)186/Final, agreed Study Plan and MediTox Standard Operating Procedures.

Date: 03.11.2020

Signature: ....

Jan Novák
Study Director

STATEMENT OF THE MANAGEMENT

According to the conditions of the basic contract PQ/112/2019 from November 26, 2019 between vivo Science GmbH (as Study Monitor) and MediTox s.r.o. (as Test Facility) "HECOLCAP 90-day Subchronic Toxicity Study after Intra-osseous Implantation in Rabbits" has been performed in compliance with EN ISO 10993-6, EN ISO 10993-11 and OECD Principles of Good Laboratory Practice.

Date: 02.11.2020 ....

Signature: ....

Jan Zábský
Test Facility Manager

ABBREVIATIONS

C	Control group
G1	Treated group - HECOLCAP
G2	Treated group – HECOLCAP + vancomycin
GLP	Good Laboratory Practise
h	Hours
i.m.	Intramuscular
i.v.	Intravenous
M	Male
NA	Not applicable
No.	Number
phf	Per High Powered
QAU	Quality Assurance Unit
RT	Room Temperature
rpm	Revolutions per minute
SOP	Standard Operating Procedure
TI	Test Item

Haematology:

APTT	Activated partial thromboplastin time
BAS	Count of basophils
EOS	Count of eosinophils
HCT	Haematocrit
HGB	Haemoglobin level
LYM	Count of lymphocytes
MCV	Mean corpuscular volume
MCH	Mean corpuscular haemoglobin
MCHC	Mean corpuscular haemoglobin concentration
MON	Count of monocytes
NEU	Cunt of neutrophils
PLT	Count of platelets
PT	Prothrombin time
RBC	Red blood cell (erythrocyte) count
WBC	White blood cell (leucocyte) count

Clinical Chemistry:

ALT	Alanine aminotransferase
Alb	Albumin
Alb/Glo	Albumin/Globulin ratio
ALP	Alkaline phosphatase
AST	Aspartate aminotransferase
Bil	Bilirubin total
Ca	Calcium
Chol	Cholesterol Total
Cl	Chloride
Crea	Creatinine
GGT	Gamma-Glutamyl Transpeptidase
Glo	Globulin

Glu	Glucose
K	Potassium
LDH	Lactate dehydrogenase
Na	Sodium
P	Phosphorus
TP	Total protein
TGC	Triglycerides
Urea	Blood urea nitrogen

Urinalysis:

Bil	Bilirubin
Ery	Erythrocytes
Glu	Glucose
Ket	Ketones
Leu	Leucocytes
neg	Negative
Nit	Nitrites
norm	Normal
pos	Positive
Pro	Protein
SG	Specific gravity
Turb	Turbidity
Ubg	Urobilinogen

Pathology

Grade 1	Minimal/very few/very small
Grade 2	Slight/few/small
Grade 3	Moderate/moderate number/moderate size
Grade 4	Marked/many/large
)	Finding unilateral in paired organs
P	Finding present, severity not scored
-	Organ examined, no pathology findings
/	Organ not examined

SUMMARY

The purpose of this study was to determine local effect and systemic toxicity of the Test Item, HECOLCAP (heparinized nanohydroxyapatite/collagen granules with and without vancomycin), after single intra-osseous implantation to the femur in rabbits for 90 days.

The study was carried out in 15 male rabbits + 3 spare animals obtained from Velaz s.r.o. divided into one control group (Group C) and two treated groups (Group G1 and G2). Application of the Test Item was done by surgery into both femurs. Three implant beds for each femur were prepared by intermittent drilling with a 2 mm drill bit and 6 mm deep cavities were created. Implants of size 2 x 6 mm were inserted into prepared holes in bone.

Each rabbit in the Group G1 was given six implants (3 to each femur) of HECOLCAP granules without vancomycin in dose 720 mg/animal (120 mg granules per one implant). Each rabbit in the Group G2 was given six implants (3 to each femur) of HECOLCAP granules with vancomycin in the same dose as Group G1.
Rabbits in the control Group C did not receive any material, only holes in the bone were drilled in the same regime as in the test groups.

All animals were observed for clinical signs, morbidity or mortality once a day during acclimatization and twice a day during study periods. Detailed clinical observation (local tolerance) of implantation sites in both femurs for nature and extent of any tissue reaction was performed once a day during the entire study period.

The body weight was measured at delivery, before the application, then weekly and before the necropsy. Individual body temperature measurement was performed before the administration, then weekly and before the necropsy. Individual food consumption was recorded weekly.

Haematology and clinical chemistry were performed before administration on the Week -1 (Examination 1), on the Week 2 (Examination 2), on the Week 4 (Examination 3) and before the necropsy – the Week 13 (Examination 4). Urinalysis was performed on the Day 91.

At the end of the study on the Day 91, all scheduled animals received a complete post-mortem examination. The full set of tissues was collected, weighted and fixed and histopathology examination was performed. The bone irritation index at the implantation sites was calculated by a semi-quantitative evaluation system according to ISO 10993-6.

Results

No clinical signs were recorded throughout the acclimatization and observation periods in any of the animals in the study except two cases. Relief of the left hind leg was observed in animal no. M13 (Group G2) from the Day 10 to the Day 91. Mild to severe yellow discharge from nostrils was recorded in animal no. M10 (Group G1) in the Days 40, 41 and 42 and this animal was prematurely euthanized on the Day 42, this clinical sign was not related to the Test Item implantation.

Rabbit no. M7 from the Group G1 was found dead on the Day 3. No clinical signs were recorded in this rabbit. Macroscopic examination revealed a fracture of the right hind limb.
During the implantation procedure two animals no. M11 and M15 from the Group G2 died. All deceased animals were replaced by the spare rabbits. In the other animals, surgery was without any complications and no adverse effects were recorded during and after anaesthesia. The wounds after surgery healed well in all animals.
In all animals from both treated groups and one control group, no tissue reaction in the implantation sites was recorded during the whole observation period.

All individual body weight values were within normal ranges in all the groups. Body weight decrease was recorded in two cases, one in prematurely euthanized animal no. M10 (G1) and one in deceased animal no. M3 (G1). Body weights of all other animals was stable or increased during the observation period.

The body temperature of all animals was recorded in the range between 38.4 – 39.6°C which corresponds to the physiological values for rabbits.

Decrease of food consumption was recorded in the prematurely euthanized animal no. M10 from the Group G1. Food consumption was well-balanced in all other animals.

No changes in the haematology and clinical chemistry parameters were observed during the study that could be connected with the Test Item administration.

There were observed the higher values of protein, bilirubin and urobilinogen urine concentration in G2 group (combination of HECOLCAP + vancomycin) at the end of the study period. The connection of these finding to the Test Item administration could not be excluded.
No changes in the other urinalysis parameter were observed during the study that could be directly connected with the Test Item administration.

Organ weight analysis did not find any treatment-related changes.

Both HECOLCAP and HECOLCAP with vancomycin in the dose of 720mg/animal did not cause gross or histopathological changes in the rabbits' liver and kidneys indicative of a toxic effect.

Other lesions found in the treated animals were either of spontaneous character or they were not in direct relation with the Test items.

All bone defects after intra-osseous implantation of the HECOLCAP were completely healed with presence of minimal to marked periosteal fibrosis similarly like in the control rabbits. On the contrary, there was presence of mild to severe amount of foreign body granulomas in the bone marrow around the grains of implanted material. These granulomas were in most cases lined by layer of newly formed bone. Implantation of the HECOLCAP with vancomycin caused similar findings without considerable differences.

The Irritation Index in the Group G1, evaluating local tissue reaction to the HECOLCAP implanted according to ISO 100993-10 after subscription of the control group is 7.08 (category of slight irritation).

The Irritation Index in the Group G2, evaluating local tissue reaction to the HECOLCAP with vancomycin implanted according to ISO 100993-10 after subscription of the control group is 5.76 (category of slight irritation)

Conclusion

The Test Item **HECOLCAP** applied in treated Group G1 in the dose 720 mg/animal and **HECOLCAP + vancomycin** applied in treated Group G2 in the dose 720 mg/animal did not cause any clinical signs of toxicity in all rabbits. No changes in the haematology and clinical chemistry parameters and no gross or histopathological changes were observed.
The **Irritation Index** evaluating local tissue reaction according to ISO 100993-10 after subtraction of the control group is **7.08** for the **Group 1** and **5.76** for the **Group 2.** Both values are in the category of **slight irritation.**

1. INTRODUCTION

1.1. Study Objective

The purpose of this study was to determine local effect and systemic toxicity of the Test Item, HECOLCAP (heparinized nanohydroxyapatite/collagen granules with and without vancomycin), after single intra-osseous implantation to the femur in rabbits for 90 days.

1.2. Project Staff

Sponsor:	INEB<br>R. Alfredo Allen, 208<br>4200-135 Porto,<br>Portugal
Sponsor’s Representative:	Fernando Jorge Monteiro<br>R. Alfredo Allen, 208,<br>4200-135 Porto<br>Portugal<br>fjmont@ineb.up.pt<br>+351 964098443
Study Monitor:	Vivo Science GmbH<br>Fabrikstraße 3, 48599 Gronau<br>Germany
Study Monitor Representative:	Dr. Robert Heinz Günter<br>Vivo Science GmbH<br>Fabrikstraße 3,48599 Gronau<br>Germany<br>Phone: +49 (0) 151 - 7014 - 5028<br>e-mail: robert.guenter@vivoscience.de
Test Facility:	MediTox s.r.o.,<br>Pod Zámkem 279, Konárovice 281 25,<br>The Czech Republic
Test Facility Manager:	Jan Zábský,<br>MediTox s.r.o.<br>Phone.: +420 313 129 374<br>e-mail: zabsky@meditox.eu
Study Director:	Jan Novák,<br>MediTox s.r.o.,<br>Phone: +420 313 129 374<br>e-mail: novak@meditox.eu
Test Site for Clinical Chemistry:	Department of Clinical Biochemistry and Diagnostics, City Hospital Pardubice<br>Kyjevská 44<br>The Czech Republic
Test Site Manager:	Jiří Skalický<br>Department of Clinical Biochemistry and Diagnostics, City Hospital Pardubice<br>Phone: +420 466 013 104<br>e-mail: veronika.koubova@nempk.cz
Quality Assurance Manager:	Kateřina Ptáčková<br>MediTox s.r.o.<br>Phone: +420 313 129 374<br>E-mail: ptackova@meditox.eu
Quality Assurance Personnel:	Kateřina Lacinová<br>MediTox s.r.o.<br>Phone: +420 313 129 374

	E-mail: lacinova@meditox.eu<br>Ivana Šurová<br>MediTox s.r.o.<br>Phone: +420 313 129 374<br>E-mail: surova@meditox.eu

1.3. Study Personnel

The following individuals were the primary contributors and supervisory personnel participating in this study.

Study Director:	Jan Novák,<br>MediTox s.r.o.,
Responsible Person for Dose Formulation, Handling and Preparation:	Klára Gruzovská<br>MediTox s.r.o.<br>Phone: +420 313 129 374<br>e-mail: gruzovska@meditox.eu
Clinical Observation:	Lukáš Reček,<br>MediTox s.r.o.<br>Phone.: +420 313 129 374<br>e-mail: recek@meditox.eu<br>Vladimir Pyatov,<br>MediTox s.r.o.<br>Phone.: +420 313 129 374<br>e-mail: pyatov@meditox.eu<br>Jan Novák,<br>MediTox s.r.o.
Veterinarian:	Lukáš Pánek<br>MediTox s.r.o.<br>Phone: +420 313 129 374<br>e-mail: panek@meditox.eu
Haematology, Clinical Chemistry and Urinalysis Results Evaluation:	Anna Kryszczuková<br>MediTox s.r.o.<br>Phone: +420 313 129 374<br>e-mail: hematologie@meditox.eu
Test Site for Clinical Chemistry - Principal Investigator (Sample Analysis):	Veronika Koubová,<br>Department of Clinical Biochemistry and Diagnostics, City Hospital Pardubice<br>e-mail: veronika.koubova@nempk.cz
Gross Pathology and Histopathology:	Aleš Brejcha,<br>MediTox s.r.o.<br>Phone: +420 313 129 374<br>e-mail: brejcha@meditox.eu<br>Jan Novák,<br>MediTox s.r.o.
Histopathology Peer Review:	Ivo Šteiner<br>MediTox s.r.o.<br>Phone: +420 313 129 374<br>e-mail: steiner@lfhk.cuni.cz
Supervisor, Animal Care:	Jana Nováková,<br>MediTox s.r.o.<br>Phone: +420 313 129 374<br>e-mail: jnovakova@meditox.eu
Statistical Analysis:	Věra Fialová<br>MediTox s.r.o., The Czech Republic<br>Tel: +420 608 029 793<br>e-mail: fialova@meditox.eu

1.4. Regulatory Guidelines

The study was carried out according to:

- EN ISO 10993-6: 2016. Biological evaluation of medical devices – Part 6: Tests for local effects after implantation
- EN ISO 10993-11: 2009. Biological evaluation of medical devices - Part 11: Tests for systemic toxicity
- Relevant SOPs of MediTox s.r.o.

1.5. GLP Compliance

This non-clinical laboratory study was done in strict compliance with:

the OECD Principles of Good Laboratory Practice C (97)186/Final, Directive 2004/10/EC, the Czech law No. 378/2007 and the Decree of Ministry of Health and Ministry of Agriculture of the Czech Republic No. 86/2008 of Collection of laws about Good Laboratory Practice for testing of drugs.

Quality Assurance Review: All of the relevant aspects of this study including all laboratory processes, data, and the Final Report were subject to Quality Assurance evaluations. Personnel from the Quality Assurance Unit (QAU) at MediTox s.r.o. inspected the study in accordance with the planned QA Program, relevant SOPs and the OECD GLP Principles. The QAU performed study-based inspections and facility-based and/or process-based inspections according to the yearly and monthly plans. The Statement of the QAU will be included in the Final Report.

1.6. Facilities Management and Animal Husbandry

Animal care was in compliance with the SOPs of MediTox s.r.o., the European Convention for the Protection of Vertebrate Animals Used for Experimental and other Scientific Purposes (ETS 123), the Czech Collection of laws No. 246/1992, inclusive of the amendments, on the Protection of animals against cruelty, and Public Notice of the Ministry of Agriculture of the Czech Republic, Collection of laws No. 419/2012 as amended, on keeping and exploitation of experimental animals. MediTox s.r.o. is a holder of the Accreditation Certificate for users issued by Central Committee for Animal Protection of the Czech Republic.

1.7. Animal Welfare Act Compliance

The study design was approved by the Institutional Animal Care and Use Committee (IACUC) and the Committee for Animal Protection of the Ministry of Health of the Czech Republic (3/2020). Procedures used in Study Plan were designed to conform to accepted practices and to minimize or avoid causing pain, distress, or discomfort to the animals.
The number of animals selected for use in this study is considered to be the minimum number necessary to meet scientific and regulatory guidelines for this type of study.

2. TEST CONDITIONS

2.1. Experimental Design

**Procedure:**	**Date:**
Study Initiation Date:	March 03, 2020
Animal Receipt:	March 03, 2020<br>March 12, 2020 (Group G2° – M16, M17)<br>March 24, 2020 (Group G1° – M18)
Health Check:	March 03, 2020<br>March 12, 2020 (Group G2° – M16, M17)<br>March 24, 2020 (Group G1° – M18)
Randomization:	March 03, 2020
Acclimatization:	7-19 days
Initiation of Experimental Part:	March 03, 2020
Test Item Implantation:	Day 1:<br>March 10, 2020 (Group C)<br>March 11, 2020 (Group G2)<br>March 12, 2020 (Group G1)<br>March 31, 2020 (Group G2° – M16, M17, Group G1° – M18)
Dosing:	Single intra-osseous implantation
Observation period:	90 days
Mortality and Clinical Observation:	Daily Twice
Detailed Clinical Observation – Implantation Site	Daily
Body Weight:	At delivery and then weekly
Food Consumption:	Weekly
Body Temperature (rectal):	Weekly
Haematology and Clinical Chemistry:	Week -1: Examination 1<br>March 05, 2020 (all groups)<br>March 17, 2020 (Group G2° – M16, M17)<br>March 26, 2020 (Group G1° – M18)<br>Week 2: Examination 2<br>March 17, 2020 (Group C)<br>March 18, 2020 (Group G2)<br>March 19, 2020 (Group G1)<br>April 07, 2020 (Group G2° – M16, M17, Group G1° – M18)<br>Week 4: Examination 3<br>March 31, 2020 (Group C)<br>April 01, 2020 (Group G2)<br>April 02, 2020 (Group G1)<br>April 21, 2020 (Group G2° – M16, M17, Group G1° – M18)<br>Week 13: Examination 4<br>June 02, 2020 (Group C)<br>June 03, 2020 (Group G2)<br>June 04, 2020 (Group G1)<br>June 23, 2020 (Group G2° – M16, M17, Group G1° – M18)
Urinalysis:	Day 91:<br>During the necropsy

Scheduled Necropsy:	Day 91:<br>June 08, 2020 (Group C)<br>June 09, 2020 (Group G2)<br>June 10, 2020 (Group G1)<br>June 29, 2020 (Group G2° – M16, M17, Group G1° – M18)
End of the Experimental Part:	August, 2020
Interim Report Date (without histopathology):	August 17, 2020
Draft Report Date:	October 12, 2020
Study Completion Date (Final Report):	November 03, 2020

°Spare animals

2.2. Description of Test Item

**Name of the Test Item**:	**HECOLCAP**
Description:	White granules with the following dimensions: 1.18-2.00 mm (diameter)
Batch No.:	24/01/2020
CAS No.:	NA
Pharmacodynamics effect:	Releasing the antibiotic during approximately 3 weeks to eliminate a bone infection and subsequently promote bone regeneration
Summary formula:	Hydroxyapatite [$Ca_{10}(PO_4)_6(OH)_2$] + type I collagen + heparin + vancomycin
Expiry:	24/01/2021
Solubility:	Granules without vancomycin partially soluble in acidic conditions
Storage conditions:	Room temperature in exicator, in supplied container - sterile centrifuge tube
Sterility:	Yes, ethanol 70 % and subsequently washing with ultrapure water in laminar flow hood chamber
Stability:	1 year from production date
Certificate of analysis:	24/01/2020
Verification of Test Item identity:	28/02/2020
Verification of additional Test Item identity:	16/04/2020
Handling of the unused Test Item:	Disposal

**Test Item formulation 1**:	**HECOLCAP granules without vancomycin**
Way of application:	Bone implantation
Formulation ready to use.:	Yes

**Test Item formulation 2**:	**HECOLCAP granules with vancomycin**
Way of application:	Bone implantation
Formulation ready to use.:	No
Additive:	Vancomycin solution
Maximum concentration in vehicle:	1 mL of 50 mg/mL of vancomycin/ 20 mg of granules
Stability:	48 h
Storage conditions:	Room temperature

Vancomycin identification:

**Name**:	**Vancomycin Hikma, 500 mg, powder for solution for infusion**
Supplier:	Hikma Farmacêutica (Portugal), S.A
Batch No.:	1809060.1
Storage conditions:	Room temperature, protect from light, in supplied container
Expiry:	06/2020
Handling of the unused Test Item:	Disposal

Vehicle for the Preparation of Vancomycin Solution:

**Name:**	**Aqua pro injection Braun**
Supplier:	B|Braun
Batch No.:	190318163
Storage conditions:	Up to 25°C
Expiry:	12/2021

2.3. Preparation of Test Item Formulation

The Test Item was delivered to MediTox s.r.o. as granules for intra-osseous implantation. Granules without vancomycin (Group G1) were prepared as ready to use. Granules with vancomycin (Group G2) were prepared as follows: 20 mg of HECOLCAP granules in Eppendorf tubes was immersed in 1 mL of vancomycin solution with a concentration of 50 mg/mL. The antibiotic adsorption onto granules was performed at 37 ºC and 120 rpm in an orbital shaker for 2 h.

2.4. Test System

Species & Strain:	Rabbits, New Zealand White
Supplier:	Velaz s.r.o., The Czech Republic
Source:	Petr Kočár, Ratibořice, The Czech Republic
Status at delivery:	Conventional
Age on delivery:	Adult rabbit
Weight at delivery:	2.5 – 3.0 kg
Total number of animals:	15 (15 males) + 3 additional spare rabbits
Number of groups:	3 (3 treated group)
Number of animals per group:	5 (5 males)
Animal identification:	Ear tattoo

2.4.1. Justification for Test System

Rabbit is a suitable animal model for conducting non-clinical evaluations of local effects after implantation. Rabbit has faster skeletal change and bone turnover and is commonly used in musculoskeletal research. Rodents are not suitable for this kind of study because of the production of necessary bone defects above critical size.

2.4.2. Allocation and Dosing

Group designation	Test Item	Dose	Animal numbers
C	Control	0 mg/animal	M1, M2, M3, M4, M5
G1	HECOLCAP	720 mg/animal	M6, M7, M8, M9, M10, M18°
G2	HECOLCAP + vancomycin	720 mg/animal	M11, M12, M13, M14, M15, M16°, M17°

°Spare animals

2.5. Animal Selection, Randomization and Group Assignment

All rabbits were randomly assigned to their respective treatment groups. The body weights required for randomization were done at delivery.

2.6. Justification of Dose level and Route

The doses and routes of intra-osseous administration to be used in this study were determined on basis of the Sponsor´s requirement and previous non-GLP tests in sheep and approved by the Study director.

2.7. Housing

Rabbits were individually housed in conventional conditions in the building No. 5, room No. 1, in environmentally monitored and ventilated rooms maintained at a temperature of 15 - 21°C and a relative humidity of 30 – 70 %. Fluorescent lighting was provided illumination for 12 hours per day. Cleaning of cages and surrounding area was performed on a daily basis. Feed and water containers were changed and sanitized at least once weekly.

2.8. Diet

The animals were fed with standard pelletized rabbit diet of monitored quality (analyzed two times per year for possible toxic or microbiological contamination – the certificates are available in MediTox s.r.o. archive) during the acclimatization and study periods. Feed KKV (Sehnoutek, Czech Republic) was provided *ad libitum*.
No contaminants were detected at levels that could reasonably be expected to affect the purpose or integrity of the study.

2.9. Water

Water of monitored quality (from own source, analyzed minimally two times per year for possible toxic or microbiological contamination) was supplied *ad libitum* during the acclimatization and study periods. The certificates are available in MediTox s.r.o. archive.
No contaminants were detected at levels that could reasonably be expected to affect the purpose or integrity of the study.

2.10. Acclimatization

The animals were acclimatized for 7 days (Group C), 8 days (Group G1) and 9 days (Group G2). Spare animals no. 16 and 17 from Group G2 were in acclimatization 19 days and spare animal no. 18 from Group G1 7 days. No prophylactic or therapeutic treatment was administered during the acclimatization or study periods. Only animals in good health conditions were used for the study.

2.11. Animal Identification

Each rabbit was individually identified by ear tattoo according to SOP SN-TOX-00. Each cage was identified with the following information: study number, group, dose, number and sex of each animal.

3. METHODS

3.1. Implantation Procedure

All necessary precautions were taken to ensure aseptic application planned using surgical procedure into the femur. The surgical sites, medial part of femur, were prepared by clipping the fur on both hind limbs from the knees to above the hips. The skin was incised and the subcutaneous and muscle tissue was dissected all the way down to the bone. Three implant beds in central diaphysis at a distance of approximately 1 cm in between were prepared in each femur. By intermittent drilling with a 2 mm drill bit during irrigation with saline, 6 mm deep cavities reaching into the medulla were created. Implants of size 2 x 6 mm were inserted into prepared holes in bone. During the surgery, the animals were under anaesthesia, see 3.2.

Each rabbit in the group G1 was given six implants (3 to each femur) of HECOLCAP granules without vancomycin in dose 720 mg/animal (120 mg granules per one implant). Each rabbit in the group G2 was given six implants (3 to each femur) of HECOLCAP granules with vancomycin in dose 720 mg/animal (120 mg granules per one implant).
Rabbits in the group C received no material, only holes in the bone were drilled in same regime as in the test groups.

The day of the Test Item implantation was designated as the Day 1 of the study. Observation period followed for 90 days after the implantation.

The animals were fasted overnight for approximately 12 h prior the implantation. After the surgery, food and water was be provided *ad libitum*.

3.2. Anaesthesia

During the surgery, the animals were under anaesthesia under supervision of the veterinarian.
Course of the anaesthesia: A Medetomidine/Midazolam/Butorphanolum was used for premedication to provide the optimum conditions for anaesthesia. The anaesthesia was performed by Ketamine.

Type:	Dose:	Application:	Purpose:
Medetomidine (Domitor, Orion Pharma)	0.12 mg/kg	*i.m.*	Premedication
Midazolam (Dormicum, Roche)	1 mg/kg	*i.m.*	Premedication
Butorphanolum (Nalgosed, Bioveta a.s.)	0.1 mg/kg	*i.m.*	Premedication
Ketamine (Narkamon, Bioveta a.s.)	35 mg/kg	*i.m.*	Anaesthesia

Anaesthesia with isoflurane (2 %) administered through face mask was performed.
After the finalization of the surgery, the anaesthesia was terminated by *i.m.* injection of atipamezole, see below.

Type:	Dose:	Application:	Purpose:
Atipamezole (Antisedan, Orion Pharma)	1 mg/kg	*i.m.*	Reversion of anaesthesia

3.3. Postoperative Care

Postoperative care was based on the decision of veterinarian and it consisted of protective collars for rabbits for 7 days.

3.4. Clinical Observations and Mortality

Daily Observations:
All animals were observed for clinical signs, morbidity or mortality once a day during acclimatization and twice a day during study periods.

Clinical Observations included: Signs of toxicity, changes in skin, fur, eyes, and mucous membranes, occurrence of secretions and excretions and autonomic activity (e.g. lacrimation, piloerection, pupil size, and unusual respiratory pattern). Changes in gait, posture and response to handling as well as the presence of clonic or tonic movements, stereotypes (e.g. excessive grooming, repetitive circling) or bizarre behaviour (e.g. self-mutilation, walking backwards) was also included.

Observations were carried out according to SOPs SN-TOX-00 and SN-TOX-09. Moribund animal was removed, sacrificed, and examined histopathologically.

One animal found dead after developed rigor mortis was examined only macroscopically.

Detailed Clinical Observation: All animals were observed for nature and extent of any tissue reaction of the implantation sites once a day during the entire study period.

3.5. Body Weight

All the animals were individually weighed at delivery, before the application, then weekly and before the necropsy.

3.6. Body Temperature

Individual body temperature measurement was performed before the administration, then weekly and before the necropsy. Body temperature was measured rectally.

3.7. Food Consumption

Individual food consumption was recorded weekly.

3.8. Clinical Pathology

3.8.1. Haematology and Clinical Chemistry

Blood samples for haematology and clinical chemistry were collected from all animals before administration on the Week -1 (Examination 1), on the Week 2 (Examination 2), on the Week 4 (Examination 3) and before the necropsy, Week 13 (Examination 4). The animals were fasted for approx. 12-18 hours before blood sampling, but water was provided *ad libitum*.

Blood samples were taken by venepuncture from *v. saphena* into tubes Vacuette, Greiner bio-one, containing K_3EDTA (haematology), sodium citrate (coagulation) and TAPVAL (without anti-coagulant for serum biochemistry).

ABX PENTRA 60 C+ was used for determination of haematology parameters from whole blood samples with K_3EDTA. Plasma samples from sodium citrate blood samples were obtained by centrifugation at 4000 rpm for 15 minutes and coagulation parameters were determined by Coagulometer STart 4.

Smears for differential leucocyte count were performed, stained by May-Grünwald and Giemsa-Romanowski and analyzed microscopically.

Serum samples were obtained by centrifugation at 6000 rpm for 15 minutes. Serum for clinical chemistry was transferred into appropriately labelled and sealed Eppendorf tubes and frozen at -20° C or below until transport to analyses (SOP-HEM, SOP-BCH).

Test Facility (Laboratory of Haematology and Biochemistry) was responsible for sample preparation and transport of serum samples to the Test Site. The Test Site (Clinical Biochemistry Dept. of Hospital Pardubice) was responsible for determination of biochemical parameters of serum samples by Dimension Vista® 1500. Raw data results were delivered to the MediTox s.r.o. Laboratory of Haematology and Biochemistry. Results evaluation, reporting and archiving of Test Site raw data were performed by Test Facility (Laboratory of Haematology and Biochemistry).

Parameters monitored:

Haematology

Parameter	Name of instrument/Method	Unit of measure (SI)
Leucocyte count (WBC)	ABX PENTRA 60 C+, Horiba	$10^9/l$
Differential leucocyte count:<br>Lymphocytes count (LYM)<br>Monocytes count (MON)<br>Neutrophils count (NEU)<br>Eosinophils count (EOS)<br>Basophils count (BAS)	Panoptically (May-Grünwald, Giemsa Romanowski) stained slides of peripheral blood, microscopic analysis	$10^9/l$
Erythrocyte count (RBC)	ABX PENTRA 60 C+, Horiba	$10^{12}/l$
Haematocrit (HCT)	ABX PENTRA 60 C+, Horiba	l/l
Haemoglobin (HGB)	ABX PENTRA 60 C+, Horiba	g/l
Mean corpuscular haemoglobin (MCH)	ABX PENTRA 60 C+, Horiba	pg
Mean corpuscular haemoglobin concentration (MCHC)	ABX PENTRA 60 C+, Horiba	g/l
Mean corpuscular volume (MCV)	ABX PENTRA 60 C+, Horiba	fl
Platelet count (PLT)	ABX PENTRA 60 C+, Horiba	$10^9/l$
Activated partial thromboplastin time (APTT)	Coagulometer STart 4, Diagnostica Stago, PTT Automate ⑤	sec.
Prothrombin time (PT)	Coagulometer STart 4, Diagnostica Stago, Neoplastine® CI PLUS ⑤	sec.

Clinical Chemistry

Parameter	Name of instrument/Method	Unit of measure (SI)
Alanine aminotransferase (ALT)	Dimension Vista® 1500, Siemens Healthcare Diagnostics/Dimension ALTI	µkat/l
Albumin (Alb)	Dimension Vista® 1500, Siemens Healthcare Diagnostics/Dimension ALB	g/l
Albumin/Globulin ratio (Alb/Glo)	Calculated	-
Alkaline phosphatase (ALP)	Dimension Vista® 1500, Siemens Healthcare Diagnostics/Dimension ALPI	µkat/l
Aspartate aminotransferase (AST)	Dimension Vista® 1500, Siemens Healthcare Diagnostics/Dimension AST	µkat/l
Bilirubin Total (Bil)	Dimension Vista® 1500, Siemens Healthcare Diagnostics/Dimension TBil	µmol/l
Calcium (Ca)	Dimension Vista® 1500, Siemens Healthcare Diagnostics/Dimension CA	mmol/l
Chloride (Cl)	Dimension Vista® 1500, Siemens Healthcare Diagnostics/Dimension Cl^-	mmol/l
Cholesterol Total (Chol)	Dimension Vista® 1500, Siemens Healthcare Diagnostics/Dimension CHOL	mmol/l
Creatinine (Crea)	Dimension Vista® 1500, Siemens Healthcare Diagnostics/Dimension CREA	µmol/l
Gamma-Glutamyl Transpeptidase (GGT)	Dimension Vista® 1500, Siemens Healthcare Diagnostics/Dimension GGT	µkat/l
Glucose (Glu)	Dimension Vista® 1500, Siemens Healthcare Diagnostics/Dimension GLU	mmol/l
Globulin (Glo)	Calculated	g/l
Lactate Dehydrogenase (LDH)	Dimension Vista® 1500, Siemens Healthcare Diagnostics/Dimension LDI	µkat/l
Phosphorus (P)	Dimension Vista® 1500, Siemens Healthcare Diagnostics/Dimension PHOS	mmol/l
Potassium (K)	Dimension Vista® 1500, Siemens Healthcare Diagnostics/Dimension K^+	mmol/l
Sodium (Na)	Dimension Vista® 1500, Siemens Healthcare Diagnostics/Dimension Na^+	mmol/l
Total protein (TP)	Dimension Vista® 1500, Siemens Healthcare Diagnostics/Dimension TP	g/l
Triglycerides (TGC)	Dimension Vista® 1500, Siemens Healthcare Diagnostics/Dimension TRIG	mmol/l
Urea	Dimension Vista® 1500, Siemens Healthcare Diagnostics/Dimension BUN	mmol/l

3.8.2. Urinalysis

Urine from the animals was collected during the necropsy on the Day 91 by puncture of the bladder. Total volume was recorded. Macroscopic analysis and semi-quantitative biochemical analysis were performed on Analyzer Urilyzer® 100 (Pro), Analyticon® Biotechnologies AG, strips Combi Screen PLUS, Analyticon® Biotechnologies AG.

Parameters monitored:

Urinalysis

Parameter	Name of instrument/Method	Unit of measure (SI)
Volume	-	ml
Appearance, color	Visually	-
Specific gravity (SG)	Urilyzer® 100 (Pro), Combi Screen PLUS, Analyticon® Biotechnologies AG	kg/l
pH	Urilyzer® 100 (Pro), Combi Screen PLUS, Analyticon® Biotechnologies AG	-
Protein (Pro)	Urilyzer® 100 (Pro), Combi Screen PLUS, Analyticon® Biotechnologies AG	g/l
Bilirubin (Bil)	Urilyzer® 100 (Pro), Combi Screen PLUS, Analyticon® Biotechnologies AG	µmol/l
Urobilinogen (Ubg)	Urilyzer® 100 (Pro), Combi Screen PLUS, Analyticon® Biotechnologies AG	µmol/l
Erythrocytes (Ery)	Urilyzer® 100 (Pro), Combi Screen PLUS, Analyticon® Biotechnologies AG	Ery/µl
Ketones (Ket)	Urilyzer® 100 (Pro), Combi Screen PLUS, Analyticon® Biotechnologies AG	mmol/l
Nitrite (Nit)	Urilyzer® 100 (Pro), Combi Screen PLUS, Analyticon® Biotechnologies AG	-
Leucocytes (Leu)	Urilyzer® 100 (Pro), Combi Screen PLUS, Analyticon® Biotechnologies AG	Leu/µl
Glucose (Glu)	Urilyzer® 100 (Pro), Combi Screen PLUS, Analyticon® Biotechnologies AG	mmol/l

3.9. Terminal Observation

Two rabbits no. M11 and M15 from Group G2 died during the implantation procedure on the Day 1. No post-mortem examination was performed in this case and these two animals were replaced by two spare rabbits no. M16 and M17, see Study Plan Amendment 03. One rabbit no. M7 (G1) was found dead, macroscopically examination was performed and organs were taken. Based on the Sponsor's decision, histopathology examination was not performed in this animal due to the short time after implantation procedure, see Study Plan Amendment 05. Animal no. M18 was included into the study instead of this deceased animal. Animal no. M10 was euthanized due to poor health condition, complete post-mortem examination was performed and full set of tissues were collected and fixed. All other animals survived to their scheduled euthanasia (preparation for euthanasia of animals - Exagon) on the Day 91 and received complete post-mortem examination and a full set of tissues were collected and fixed as per the list given below in 3.9.2.

3.9.1. Necropsy

All the animals were weighed and examined externally. The external surface of the body and all orifices of the body were examined. The cranial, thoracic and abdominal cavities were opened and examined macroscopically. Implant sites were macroscopically examined for alterations of the normal structure. For better identification, each implantation site in both femurs was specified as number 1-3 (from distal to proximal direction) and by the letter L or R respectively according to left femur or right femur**.** Any abnormalities were recorded with details of location, color, shape and size.

3.9.2. Pathology Procedures

Table 1: The organs with histopathology examination

Tissue/Organ	Weight	Fix	Slide	Microscopy
Adrenal glands	×	×	×	×
Aorta		×	×	×
Brain	×	×	×	×
Bone (femur with joint and cartilage)		×	×	×
Cecum		×	×	×
Colon		×	×	×
Duodenum		×	×	×
Epididymides *	×	×	×	×
Eyes (incl. optic nerves) *		×	×	×
Heart	×	×	×	×
Ileum		×	×	×
Implantation sites (3 from each femur)		×	×	×
Jejunum		×	×	×
Kidneys *	×	×	×	×

Tissue/Organ	Weight	Fix	Slide	Microscopy
Lacrimal gland		×	×	×
Larynx		×	×	×
Liver	×	×	×	×
Lungs (incl. mainstem bronchi)		×	×	×
Lymph node (iliac, cervical, mesenteric)		×	×	×
Oesophagus		×	×	×
Pancreas		×	×	×
Payers patches		×	×	×
Pituitary		×	×	×
Prostate	×	×	×	×
Salivary gland (mandibular, parotid) *		×	×	×
Seminal vesicles		×	×	×
Sciatic nerve		×	×	×
Skeletal muscle		×	×	×
Skin		×	×	×
Spinal cord (at three levels)		×	×	×
Spleen	×	×	×	×
Sternum (incl. bone marrow)		×	×	×
Stomach		×	×	×
Testes *	×	×	×	×
Thymus	×	×	×	×
Thyroids (incl. parathyroid) *	×	×	×	×
Tongue		×	×	×
Trachea		×	×	×
Ureters		×	×	×
Urinary bladder		×	×	×
All gross lesions		×	×	×

* paired organs

3.9.3. Organ Weight

The organs specified in the section 3.9.2, Table 1 were weighed after trimming of fat and other contiguous tissues. Contra-lateral organs were weighed together. Organ weights were not determined for any animal found dead or sacrificed prematurely during the study. Organ weights and terminal body weights were used for the calculation of organ-to-body weight ratios.

3.9.4. Fixation

The tissues listed in Table 1 were preserved in 4% neutral buffered formaldehyde. The eyes, optic nerves, testes and epididymides were fixed in Davidson's fluid for 24 hours and then transferred to 4% neutral buffered formaldehyde.

3.9.5. Histotechnique

The tissues collected from all the scheduled animals were processed, wax embedded, cut at a nominal thickness of approx. 5µm, stained with haematoxylin and erytrosin. Bones and implantation sites were decalcified with formic acid (SOP PAT).

3.9.6. Histopathology

Local biological effects after intra-osseous implantation were determined by the semi-quantitative scoring system according to ISO 10993-6, see Tables 2-3. Histological characteristic such as capsule formation, inflammation, presence of polymorphonuclear cells, giant cells, plasma cells or degradation of material were evaluated for each implantation site.
Full histopathology was carried out, apart from deceased animals no. M7 (G1), M11 and M15 from the Group G2, on the preserved organs and tissues in moribund rabbit no. M10 (G1) euthanized prematurely, and in all the remaining animals in this study surviving to their scheduled necropsy.

Table 2: Histological evaluation system — Cell type/response

Cell type/response	Score 0	1	2	3	4
Polymorphonuclear cells	0	Rare,1-5/phf	5-10/phf	Heavy infiltrate	Packed
Lymphocytes	0	Rare,1-5/phf	5-10/phf	Heavy infiltrate	Packed
Plasma cells	0	Rare,1-5/phf	5-10/phf	Heavy infiltrate	Packed
Macrophages	0	Rare,1-5/phf	5-10/phf	Heavy infiltrate	Packed
Giant Cells	0	Rare,1-5/phf	5-10/phf	Heavy infiltrate	Sheets
Necrosis	0	Minimal	Mild	Moderate	Severe

phf = per high powered (400 ×) field.

Table 3: Histological evaluation system — Response

Response	Score 0	1	2	3	4
Neovascularisation	0	Minimal capillary proliferation, focal, 1-3 buds	Groups of 4-7 capillaries with supporting fibroblastic structures	Broad band of capillaries with supporting structures	Extensive band of capillaries with supporting fibroblastic structures
Fibrosis	0	Narrow band	Moderately thick band	Thick band	Extensive band
Fatty Infiltrate	0	Minimal amount of fat associated with fibrosis	Several layers of fat and fibrosis	Elongated and broad accumulation of fat cells about the implant site	Extensive fat completely surrounding the implant

Individual score from Table 2 – Cell type/response and Table 3 - Response were added into Semi-quantitative evaluation system table. Total value for each implant site and then, mean irritation score for each animal was calculated according to semi-quantitative evaluation system. Group mean value of the numerical score of irritation was calculated for the treated groups and for control group. The difference between mean value of the treated group and mean value of the control group indicates the irritation index of the Test Item. The negative difference was determined as 0. The values of irritation index are stated according to ISO 10993-6: Prematurely euthanized animal M10 was excluded from the Semi-quantitative evaluation system. Group G1 mean value for Irritation index was calculated from only four animals.

Irritation index	
**Mean value**	**Reaction**
0.0 - 2.9	Non-irritant
3.0 – 8.9	Slight irritant
9.0 – 15.0	Moderate irritant
> 15	Severe irritant

3.10. X-ray Examination

After necropsy, both femurs from each rabbit (implantation sites in two planes) were x-rayed. The X-ray photos were made to check the bone status and implants position. The X-ray photos were taken by a veterinarian and these photos were printed and attached to the Final Report**.**

X-ray device:

**Name of X-ray device:**	GIERTH HF 200A
**Description:**	High – frequency X-ray generator with full bridge inverter system
**Supplier:**	GIERTH X-Ray international GmbH
**Output in 2kV steps:**	30 – 60 kV = 40 mA (max.)<br>62 – 70 kV = 35 mA (max.)<br>72 – 80 kV = 30 mA (max.)<br>82 – 100 kV = 25 mA (max.)
**X-ray tube:**	Toshiba, Type D-124 S
**Focal spot:**	1.2 mm x 1.2 mm
**mAs:**	0,3 – 60 mAs
**Inverter frequency:**	120 kHz
**Power requirement:**	2.5 kVA

Note: X-ray device is not included in the List of equipment of MediTox s.r.o and was supplied by the veterinarian (non-GLP). It is sufficient for this purpose without impact on the study.

3.11. Data Compilation

Statistical software GraphPad Prism (version 8.1.2., last internal validation August 2020) was used for statistical evaluation in this study.
Data were analyzed to determine whether analyzed parameters of treated groups (G1 and G2) are significantly different from control group (C) at the 95.0 % confidence level.
Analyzed data were tested for normality (Kolmogorov-Smirnov test) and homogeneity of variance (Bartlett´s test or F test).
Body Weight, Body Temperature and Food Consumption Data were separately analyzed using ANOVA followed by Dunnett´s Multiple Comparison Test. Haematology, Clinical Chemistry and Organ Weight data were analyzed using ANOVA followed by Tukey´s Multiple Comparison Test. The F-test in the ANOVA tests whether there are any significant differences amongst the means at the 95.0 % confidence level. With small sample sizes (less than 10), the normality of the data becomes increasingly uncertain and nonparametric methods such as Kruskal-Wallis test (which compares medians instead of means) are more appropriate, so we used it too, followed by Dunn´s Multiple Comparison Test.
Statistically significant difference at the 95.0 % confidence level is pointed up in tables in bold. The difference between treated group (G1 and G2) and control group (C) is marked by asterisk. One asterisk (*) is used for significant difference only between means. Two asterisks (**) were used in cases for significant difference only between medians. Three asterisks (***) denote statistically significant difference between means and medians at the 95.0 % confidence level.

4. RESULTS

4.1. Clinical Observation and Mortality

All the animals were in good health condition throughout the acclimatization and observation periods in any of the animals in the study except two cases. Relief of the left hind leg was observed in animal no. M13 from the Group G2, from Day 10 to Day 91 of the study. This symptom was probably caused by the fractured of the left hind leg at the implantation site which was confirmed by the macroscopic examination. Mild to severe yellow discharge from nostrils was recorded in animal no. M10 (Group G1) in the Days 40, 41 and 42, this animal was prematurely euthanized on the Day 42, this clinical sign was not to related to the Test Item implantation.

One rabbit no. M7 from the Group G1 was found dead on the Day 3. No clinical signs were recorded in this rabbit. The cause of death was probably a fracture of the right hind limb and subsequent stress after the surgery.

All the animals were anesthetized with an appropriate combination of anaesthetics. During the implantation procedure two rabbits no. M11 and M15 from the Group G2 died. In the other animals, surgery was without any complications and no adverse effects were recorded during and after anaesthesia.
The wounds after surgery healed well in all animals. No clinical findings or signs of toxicity were observed after implantation of Test Item materials or during whole observation period.

4.1.1. Detailed Clinical Observation

In all animals from both treated groups and one control group, no tissue reaction in the implantation sites was recorded during the whole observation period.

4.2. Body Weight

Individual values of the body weight are presented in Supplements I, Table Set I, Tables 1-3. Mean, median and SD values are presented in Table Set I, Table 4 and Graph Set I, Graph 1.

All individual body weight values were within normal ranges in all the groups. The mean body weights were stable or increased during the observation period in all groups. However, body weight decrease was recorded in one prematurely euthanized animal no. M10 (G1) between the Days 36-42 before the premature euthanasia and in one deceased animal no. M3 between the Days 1-3.
The statistical evaluation did not find any significant difference of the means and medians during the whole study period when compare control group C with Group 1 and Group 2.

4.3. Body Temperature

Individual values of the body temperature are presented in Supplements I, Table Set II, Tables 5-7. Mean, median and SD values are presented in Table Set II, Table 8 and Graph Set II, Graph 2.

All individual body temperature values were in the physiological range of rabbit. The body temperature of all animals was recorded in the range between 38.4 – 39.6°C.
Isolated statistically significant difference in median was noted in the Group G1 on the Day 43 of the study period as compared to control Group C.

4.4. Food Consumption

Individual values of the food consumption are presented in Supplements I, Table Set III, Tables 9-11. Mean, median and SD values are presented in Table Set III, Table 12 and Graph Set III, Graph 3.

Decreased of food consumption was recorded only in the prematurely euthanized animal no. M10 from the Group G1 in the week 6. Food consumption was well-balanced in all other animals.
Due to the different time range of acclimatization in animals, only the first week of Week -1 was calculated for statistical analyzation. Statistically significant differences in means were found in Group G1 in the Week -1 and in Group G2 in the Week 5 as compared to Group C. Differences in means and medians was revealed in Group G2 in the Week -1 in comparison with Group C.

4.5. Clinical Pathology

4.5.1. Haematology

Individual haematology values are presented in Supplements I, Table Set IV, Tables 13-15. Mean, median and SD values are presented in Table Set IV, Table 16.

Statistically significantly lower mean NEU value was found in G1 group as compared to control group in the Examination 1 (before the administration start), therefore the connection to the Test Item administration is excluded.

Red Blood Cells Count (RBC, HGB, HCT, MCV, MCH, MCHC)
No statistically significant changes between control and dosed groups were observed in RBC parameters and also no changes between examinations were found, that could be connected to the Test Item administration.
Some of the individual values of RBC, HGB, HCT, MCV, MCH and MCHC slightly exceeded either the lower or the upper reference limit, however no dose dependent changes were observed, all the results from the dosed groups were comparable to the results from control group.

White Blood Cells Count and Differential Leucocytes Count (WBC, NEU, EOS, BAS, LYM, MON)
Statistical analysis revealed significantly lower mean WBC and NEU values in G2 group and higher mean LYM value in G1 group in the Examination 2 as compared to control group, however the differences between Examination 1 and 2 did not exceed 10% in all three cases and exceeding of the reference limits[1] was observed.

Coagulation (PLT, APTT, PT)
The statistical analysis revealed significantly lower mean PT value in G1 group in the Examination 2 as compared to control group, however the difference between Examination 1 and 2 was only 2%. No other changes connected to the administration were found in coagulation parameters.

Reference:
[1] Reference values of haematology, Laboratory rabbit, valid until December 31, 2020, Unpublished data of MediTox s.r.o.

4.5.2. Clinical Chemistry

Individual clinical chemistry values are presented in Supplements I, Table Set V, Tables 17-19. Mean, median and SD values are presented in Table Set V, Table 20.

The statistical analysis revealed some significant differences between control and dosed groups in the Examination 1 in Ca (G2) and AST (G1). It was before the administration start, so the connection to the Test Item administration is excluded, the reason is more likely higher inter-individual variability.

The individual values of **Glu**, **Na**, **K**, **Cl**, **Ca** and **Alb/Glo** concentrations and **LDH**, **ALT** and **GGT** activities varied within the reference limits[2] during the study (or in a few cases, the values were just around the limits). No substantial differences were observed between the control and treated groups throughout the study in these parameters.

Statistical analysis revealed significantly lower mean **AST** value in G1 group as compared to control group in the Examination 2, however the decrease of serum enzyme activities is considered clinically insignificant. The same finding was recorded before administration. No other changes in AST levels that could be connected to the Test Item administration were found during the study.

The decrease of mean **ALP** values was found in all groups and all examinations by 8-65%, however again, the decrease of serum enzyme activities is considered clinically insignificant and no differences between groups were found.

There was observed the increase of mean **Urea** values in G1 group the Examination 2,3 and 4 by 33%, 42% and 48% consequently, however statistical analysis did not reveal any significant changes as compared to control group and most of the individual Urea values varied within reference limits[2] in G1 group.

Most of the individual **Bil** values exceeded the lower reference limit[2], however no statistically significant changes were found between the dosed groups and control group.

A decrease of **Chol** values (by 34-100%) was observed during the study in the Examination 2-4 as compared to the Examination 1 in all tested groups. No differences between groups were found and all of the individual values varied within reference limits[2].

Statistical analysis revealed significantly lower mean **P** value in G1 group in the Examination 2 as compared to control group, however the difference between Examination 1 and 2 did not exceed 10% in G1 group.

An increase of **TP**, **Alb** and **Glo** (by 5-30%) was observed in the Examination 4 as compared to the Examination 1 in all tested groups (including control group), however most of the individual values varied within reference limits[2] and no statistically significant changed were found between the groups during the study.

The increase of mean **Crea** values was found in the Examination 3 and 4 as compared to the Examination 1 in all groups (by 13-73%), however the increase was most pronounced in control group. Moreover, no statistically significant differences were found between control and treated groups during the study.

A decrease of mean **TGC** values was observed in all groups and examinations as compared to the Examination 1. However, the decrease was most pronounced in control group (by 37-68%) and no statistical significance was found during the study.

Reference:

[2] Reference values of clinical chemistry, Laboratory rabbit, valid until December 31, 2020, Unpublished data of MediTox s.r.o.

4.5.3. Urinalysis

Individual urinalysis values are presented in Supplements I, Table Set VI, Tables 21-23.

The samples collected from all of the animals during the study were yellow without marked turbidity, except for slight turbidity and dark yellow colour found in isolated cases throughout the study.

Since the urine samples were collected during necropsy by puncture, the relation of the urine volume value to Test Item administration is irrelevant.

The range of **pH** values varied between 7.5-9.0 in all of the animals during the study. No substantial differences were observed between the control and tested groups.

Normal values of **glucose** were observed in all the samples during the study.

Specific gravity varied predominantly within range 1.000-1.005 kg/l in all groups throughout the study. Higher values (1.010 and 1.015 kg/l) were observed in isolated cases throughout the study. No treatment related changes were found during the study.

The presence of **protein** varied between neg to 0.3 g/l in C and G1 group. Slightly higher values (0.3-5.0 g/l) were observed in G2 group.

The presence of **nitrites** was negative in all samples during the study.

Bilirubin occurrence varied between negative to 70 µmol/l in all samples during the study. Higher occurrence as compared to control group was found in G2 group (70 µmol/l found in 4 animals of 5).

Urobilinogen occurrence was normal in all of the samples in C and G1 group except for 35 µmol/l found in M18 (Group G1). Higher occurrence of urobilinogen was observed in G2 group (70 µmol/l in 2 animals and 140 µmol/l in 2 animals).

The **erythrocytes** occurrence was negative in all samples during the study except for 50 Ery/µl found in M8 (Group G1).

Leucocyte occurrence was negative in all of the samples during the study, except for 25 Leu/µl found in M16 (Group G2).

The **ketones** presence varied between negative to 1.0 mmol/l in all animals during the study except for 2.5 mmol/l found in M16 (Group G2). No substantial changes were observed between the individual groups throughout the study.

4.6. Organ Weight Analysis

Individual organ weight data are presented in Supplements I, Table Set VII, Tables 24-29. Mean, median and SD values are presented in Table Set VII, Tables 30 and 31.

The statistical analysis revealed significantly lower absolute weight of the heart in the Group G2. Significant increase was recorded in absolute adrenals and prostate weights of the Group G2. Relative weight of the heart was decreased and relative prostate weight increased in rabbits of the Group G2.

The described changes in organ weights were without any relation to clinical chemistry parameters and histopathological examination.

4.7. Gross Pathology Examination and Histology

Individual data of the gross pathology findings, microscopic findings and microscopic classification of bone reaction are presented in Supplements I, Table Set VIII, Tables 32- 71.

Deceased animals

Two rabbits (M11, M15, G2) dying during the operation procedure and one rabbit dying on Day 3 (M7, G1) have been substituted by spare animals. Male M7 (G1) was substituted by male M18 and two males M11 and M12 (G2) by animals M16 and M17, respectively. The deceased rabbits were not pathologically examined.

Euthanized animal

Male M10 (group G1) was euthanized in moribund state on day 42 of the study. The cause of its state was marked purulent bronchopneumonia and purulent pleuritis of unclear origin.

Liver

Chronic productive pericholangoitis of different degree was observed in the liver of most animals of all dose groups including controls. This lesion was characterized by portal infiltrates of lymphocytes and plasma cells with fibrosis, mostly affecting bile ducts. This lesion was most probably caused by an infection by protozoal parasite *Encephalitozoon cuniculi* which had been revealed in some rabbits in this study. Mild to marked clear cell change, characterized by clear cytoplasm of hepatocytes, was found in two control and two G2 males as a spontaneous lesion. Small focal necrosis of liver parenchyma found in one G1 male is of unclear origin, possible trauma during terminal handling could not be excluded. Venostasis, observed in the liver of most control and administered rabbits, is a terminal lesion related to the mode of euthanasia.

Kidneys

The most frequent lesion in the kidneys of rabbits of all dose groups including controls was mild focal chronic inflammation. Together with chronic pyelitis, cortical scars, and pelvic edema revealed in some rabbits, it was most probably related to the parasitic infection by *Encephalitozoon cuniculi*.

Mild venostasis, revealed in one G1 rabbit, is a terminal lesion.

There were no kidney lesions related to the Test item administered.

Lungs

Minimal to marked chronic bronchitis was observed in most of both control and administered rabbits as a spontaneous lesion.

Small solitary granuloma, observed in the lungs of one G1 male, could be caused by infection by the protozoal parasite *Encephalitozoon cuniculi*.

Focal hemorrhage and edema revealed in the lungs of some G1 and G2 animals are secondary terminal lesions without direct relation to the treatment.

Hematopoietic and lymphatic systems

Cervical lymph node of one control male showed mild hyperplasia as a spontaneous lesion.

Deposition of small amount of hemosiderin (post bleeding) of unclear origin was revealed in the iliac lymph node of one rabbit from the G2 group.

Thymus of most animals from all groups showed minimal to marked lipomatous atrophy of spontaneous origin.

Venostasis found in the spleen of some control and some administered animals is a terminal lesion.

Male reproductive system

No pathological findings were revealed in the testes of both control and treated rabbits.

Prostate gland of some rabbits from all dose groups including controls showed solitary small foci of squamous metaplasia hence relation of this lesion to the substances tested is not probable. Mild focal chronic inflammation revealed in the prostate gland of one control male is of spontaneous origin.

Site of administration (right and left femur)

The skin and thigh muscles in the operation site were in all rabbits healed without any signs of inflammation. Small amount of whitish granular substance was found in the skeletal muscles closely to the implantation site in most of rabbits from the G1 and G2 group. These substances were remnants of the Test items implanted. Fracture of the right femur with dislocation was revealed in an euthanized male M10 from the G1 group. Similar fracture was found in the left femur of male M13 from the G2 group at the end of the observation period. Implantation sites were in most cases difficult to identify. Small focal depression was found in one implantation site of the control rabbit M3 and small red foci at the implantation sites were observed in three control and one G2 animal. Small flat solid protuberances were found at the implantation sites of femurs of two control, two G1, and two G2 rabbits.

Histopathological examination revealed small foreign body granulomas around implanted granular material in the skeletal muscles of the thighs in most of G1 animals and all G2 rabbits. In two G1 and all G2 animals there were found small scars. Skeletal muscles of the control rabbits were without pathological findings.

Microscopic examination of control femurs found completely healed bone defect in all cases with minimal to marked periosteal fibrosis in most cases. The grossly observed small protuberances were composed by regular osseous tissue. It is not clear whether they are a consequence of the previous bone defect.

All bone defects in the G1 group rabbits were completely healed including the fracture of the right femur at sites of R1 and R2 defects in male M10. Similarly like in controls there was minimal to marked periosteal fibrosis. In contrary to control animals, foreign body granulomas of mild to severe extent were found in the bone marrow. They were formed around grains of implanted material. Most of granulomas were lined by newly formed bone tissue. The same granulomas were also found in adjacent skeletal muscles. The right femur fracture in male M10 was healed by ossified fibrous tissue with organizing hematoma.

Practically the same picture without considerable differences showed the bone defects in the G2 rabbits. The fracture of left femur, near to the L3 implantation site, was healed by osseous callus with fatty bone marrow.

Evaluation of Irritation index at place of implantation

Local biological effect of the Test items after intra-osseous implantation was determined by the semi-quantitative scoring system according to ISO 10993-6 (See Tables 53 – 67). Semi-quantitative evaluation of implantation sites in rabbit M10 was not included to calculation of the average irritation index in the Group G1.

The average **Irritation Index** in the **Group C** has been **1.30 (no irritation).**

The average **Irritation Index** in the **Group G1** has been **8.38 (slight irritation).**

Final Irritation Index after subscription of the control group **is 7.08 (slight irritation).**

The average **Irritation Index** in the **Group G2** has been **7.06 (slight irritation).**

Final Irritation Index after subscription of the control group **is 5.76 (slight irritation).**

Miscellaneous findings

Chronic granulomatous encephalitis, ependymitis, granulomatous inflammation in the spinal cord, and chronic meningitis, revealed in several control and administered rabbits, were caused by the protozoal parasite *Encephalitozoon cuniculi* infection. These lesions were accompanied by focal chronic inflammation in the kidneys and by chronic productive pericholangoitis in the liver.

Mild nodular hyperplasia was revealed in the thyroid gland of male M9 (G1) as a spontaneous lesion.

Minimal to mild chronic inflammation and mucosal edema of different degree were found in the trachea of several control and administered rabbits, hence their relation to the Test items implanted is not probable.

Lacrimal gland of a few control and treated rabbits revealed minimal to mild chronic inflammation.

Minimal chronic urocystitis of spontaneous origin was found in one control male.

4.8. X-ray Examination

X-ray photos and photo-documentation of the implantation sites are given in Supplements II.

Bone status of all animals in the Group C was good without any signs of fracture. In both tested groups G1 and G2, material of the Test Item was in position and radiocontrast. Due to bone weakening after the implantation procedure, a pathological fracture was observed at one implantation site in animal no. 10 (G1) and animal no. 13 (G2).

5. CONCLUSION

No clinical signs were recorded throughout the acclimatization and observation periods in any of the animals in the study except two cases. Relief of the left hind leg was observed in animal no. M13 (Group G2) from the Day 10 to the Day 91. Mild to severe yellow discharge from nostrils was recorded in animal no. M10 (Group G1) in the Days 40, 41 and 42 and this animal was prematurely euthanized on the Day 42, this clinical sign was not to related to the Test Item implantation.

In all animals from both treated groups and control group, no tissue reaction in the implantation sites was recorded during the whole observation period.

All individual body weight values were within normal ranges in all the groups. Body weight decrease was recorded in two cases, one in prematurely euthanized animal no. M10 (G1) and one in deceased animal no. M3 (G1). Body weights of all other animals was stable or increased during the observation period.

The body temperature of all animals was recorded in the range between 38.4 – 39.6°C which corresponds to the physiological values of the rabbit.

A decrease in food consumption was recorded in the prematurely euthanized animal no. M10 from the Group G1. Food consumption was well-balanced in all other animals

No changes in the haematology and clinical chemistry parameters were observed during the study that could be connected with the Test Item administration.

The highest values of protein, bilirubin and urobilinogen urine concentration were observed in G2 group at the end of the study period. The connection of these finding to the Test Item administration could not be excluded. No changes in the other urinalysis parameter were observed during the study that could be directly connected with the Test Item administration.

Organ weight analysis did not find any treatment-related changes.

Both HECOLCAP and HECOLCAP with vancomycin did not cause gross or histopathological changes in the rabbit's liver and kidneys indicative of a toxic effect.

Other lesions found in the treated animals were either of spontaneous character or they were not in direct relation with the Test items.

All bone defects after intra-osseous implantation of the HECOLCAP were completely healed with presence of minimal to marked periosteal fibrosis similarly like in the control rabbits. On the contrary, there was presence of mild to severe amount of foreign body granulomas in the bone marrow around the grains of implanted material. These granulomas were in most cases lined by layer of newly formed bone. Implantation of the HECOLCAP with vancomycin caused similar findings without considerable differences.

The Irritation Index in the Group G1, evaluating local tissue reaction to the HECOLCAP implanted according to ISO 100993-10 after subscription of the control group is **7.08** (category of **slight irritation**).

The Irritation Index in the Group G2, evaluating local tissue reaction to the HECOLCAP with vancomycin implanted according to ISO 100993-10 after subscription of the control group is **5.76** (category of **slight irritation**)

6. ARCHIVING

All data pertaining to this study will be stored in the Archives of MediTox s.r.o., Konárovice for a minimum period of 10 years. After 10 years, the Sponsor's consent will be sought and the archived material will be either destroyed or transferred to the Sponsor.
The material to be archived: Study Plan with amendments, correspondence, all the documentation and raw data related to the Test and Control Item, test system and test conditions, draft report and Final Report with statement of QAU, records of QAU inspections and the Test and Control Item reference samples.

7. AMENDMENTS AND DEVIATIONS

Amendments:

Amendment No. 01 of March 09, 2020: Addition in Post-Operative Care.

Amendment No. 02 of March 11, 2020: Change in Anaesthesia, Replacement of deceased rabbits with spare animals.

Amendment No. 03 of March 18, 2020: Change in the time schedule for spare rabbits M16, M17, Replacement of deceased rabbit with spare animal M18.

Amendment No. 04 of March 23, 2020: Delivery of additional Test Item.

Amendment No. 05 of April 06, 2020: Histopathology examination of dead rabbit.

Amendment No. 06 of May 27, 2020: Specification of implantation sites, X-ray photos and photo documentation of implantation sites.

Deviations:

Deviation 01
The germicidal radiator Prolux 9 GIP65 36W intended for the preparation of room in the surgical tract according to SOP SN-PRO-12 (Bactericidal lights) has been broken and replaced by a mobile lamp LB 301.2 with Philips TUV 30W / 630T8 germicidal tube – 46 hours operation (lifetime 8000 hours). This germicidal lamp was used on the days of surgery on March 10, 11, 12, and 31, 2020. The defect was identified prior to the start of operations and there was no impact on the integrity of the study.

This deviation did not affect in any way the integrity of the study.

Deviation 02
Slight deviations in the relative air humidity (30-70 %) were recorded during the study period. The upper prescribed limit of the relative air humidity was exceeded in 4 cases (1 hour) by max. 1.0 % (70.3 % - 71.0 %). The overall duration time was 0.0014 %.

The duration of each deviation and the total duration of up to 10% of the total study period is considered to be low risk and not affecting the animal health status or the integrity of the study

8. DISTRIBUTION

This Final Report was distributed as follows:

Print No. 1 Sponsor
Print No. 2 Study Monitor
Print No. 3 Test Facility

In addition to the paper prints, the sponsor received the final version in Word and pdf (acrobat) format.

SUPPLEMENTS I

		Number of Pages
TABLE SET I	Body Weight	2
Graph SET I	Body Weight	1
TABLE SET II	Body Temperature	2
GRAPH SET II	Body Temperature	1
TABLE SET III	Food Consumption	3
GRAPH SET III	Food Consumption	1
TABLE SET IV	Haematology	5
TABLE SET V	Clinical Chemistry	6
TABLE SET VI	Urinalysis	1
TABLE SET VII	Organ Weight	5
TABLE SET VIII	Pathology Examination	25
Statement of Quality Assurance Unit		1
GLP-certificate of the Test Facility		2

TABLE SET I

Body Weight

Table 1: Body weight - Group C (0 mg/animal) - Individual animal data(kg)

Day	At delivery	1	8	15	22	29	36	43	50	57	64	71	78	85	91
**Anim.No.**															
**M1**	2.9	2.9	2.8	3.1	3.3	3.5	3.6	3.7	3.8	3.9	3.9	4.0	4.1	4.1	4.2
**M2**	2.8	2.9	2.9	3.3	3.5	3.8	4.0	4.2	4.3	4.4	4.5	4.6	4.8	4.8	5.0
**M3**	2.8	2.9	3.0	3.3	3.6	3.8	3.8	3.8	3.8	4.0	4.2	4.3	4.4	4.4	4.4
**M4**	2.7	2.7	2.6	2.9	3.0	3.3	3.5	3.6	3.7	3.8	3.9	4.0	4.1	4.2	4.2
**M5**	2.6	2.7	2.8	3.1	3.2	3.5	3.6	3.6	3.7	3.6	3.8	3.9	3.9	3.9	4.1

Table 2: Body weight - Group G1 (720 mg/animal) - Individual animal data(kg)

Day	At delivery	1	3	8	15	22	29	36	42	43	50	57	64	71	78	85	91
**Anim.No.**																	
**M6**	2.9	3.0		3.0	3.3	3.4	3.7	3.9		4.0	4.1	4.2	4.3	4.3	4.4	4.3	4.4
**M7**	2.8	2.9	2.5	n.t.	n.t.	n.t.	n.t.	n.t.	n.t.	n.t.	n.t.	n.t.	n.t.	n.t.	n.t.	n.t.	n.t.
**M8**	2.8	2.9		2.8	2.9	3.0	3.3	3.5		3.6	3.7	3.8	3.9	4.1	4.1	4.2	4.3
**M9**	2.7	2.7		2.7	3.1	3.2	3.5	3.7		3.7	3.8	3.8	3.8	3.8	3.8	3.8	3.8
**M10**	2.5	2.6		2.5	2.6	2.6	2.8	2.9	2.5	n.t.	n.t.	n.t.	n.t.	n.t.	n.t.	n.t.	n.t.
**M18**	3.0	3.0		3.1	3.5	3.5	3.7	3.9		3.9	4.1	4.1	4.2	4.3	4.4	4.4	4.3

Table 3: Body weight - Group G2 (720 mg/animal + vancomycin) - Individual animal data(kg)

Day	At delivery	1	8	15	22	29	36	43	50	57	64	71	78	85	91
**Anim.No.**															
**M11**	2.9	3.0	n.t.	n.t.	n.t.	n.t.	n.t.	n.t.	n.t.	n.t.	n.t.	n.t.	n.t.	n.t.	n.t.
**M12**	2.9	3.0	3.0	3.4	3.5	3.8	4.1	4.2	4.2	4.4	4.4	4.5	4.4	4.3	4.4
**M13**	2.8	2.8	2.8	3.0	3.0	3.3	3.5	3.7	3.8	3.9	4.0	4.1	4.3	4.3	4.4
**M14**	2.7	2.7	2.7	3.1	3.1	3.5	3.7	3.8	3.9	4.0	4.0	4.1	4.2	4.2	4.3
**M15**	2.5	2.6	n.t.	n.t.	n.t.	n.t.	n.t.	n.t.	n.t.	n.t.	n.t.	n.t.	n.t.	n.t.	n.t.
**M16**	2.8	3.2	3.2	3.6	3.7	3.7	3.9	4.1	4.3	4.5	4.5	4.7	4.7	4.7	4.6
**M17**	2.7	2.9	3.1	3.5	3.6	3.8	3.9	4.0	4.2	4.3	4.4	4.4	4.5	4.5	4.6

Note: n.t. – not tested (deceased animal)

Table 4: Body Weight - mean, median and SD values

Group	Day	At delivery	1	8	15	22	29	36	43	50	57	64	71	78	85	91
C			x							x						
	N	5	5	5	5	5	5	5	5	5	5	5	5	5	5	5
	Mean	2.8	2.8	2.8	3.1	3.3	3.6	3.7	3.8	3.9	3.9	4.1	4.2	4.3	4.3	4.4
	Median	2.8	2.9	2.8	3.1	3.3	3.5	3.6	3.7	3.8	3.9	3.9	4.0	4.1	4.2	4.2
	SD	0.1	0.1	0.1	0.2	0.2	0.2	0.2	0.2	0.3	0.3	0.3	0.3	0.4	0.3	0.4
G1																
	N	6	6	5	5	5	5	5	4	4	4	4	4	4	4	4
	Mean	2.8	2.9	2.8	3.1	3.1	3.4	3.6	3.8	3.9	4.0	4.1	4.1	4.2	4.2	4.2
	Median	2.8	2.9	2.8	3.1	3.2	3.5	3.7	3.8	4.0	4.0	4.1	4.2	4.3	4.3	4.3
	SD	0.2	0.2	0.2	0.3	0.4	0.4	0.4	0.2	0.2	0.2	0.2	0.2	0.3	0.3	0.3
G2																
	N	7	7	5	5	5	5	5	5	5	5	5	5	5	5	5
	Mean	2.8	2.9	3.0	3.3	3.4	3.6	3.8	4.0	4.1	4.2	4.3	4.4	4.4	4.4	4.5
	Median	2.8	2.9	3.0	3.4	3.5	3.7	3.9	4.0	4.2	4.3	4.4	4.4	4.4	4.3	4.4
	SD	0.1	0.2	0.2	0.3	0.3	0.2	0.2	0.2	0.2	0.3	0.2	0.3	0.2	0.2	0.1

Note for Table 4:
Statistical evaluation by ANOVA p with Dunnett's MC test (parametric test for means) and Kruskal-Wallis test p with Dunn's MC test (non-parametric test for medians)
Statistically significant difference at the 95.0 % confidence level
x Normality test not passed (for N > 4) - non-parametric test should be used for evaluation

GRAPH SET I

Body Weight

Graph 1: Body Weight values - Means

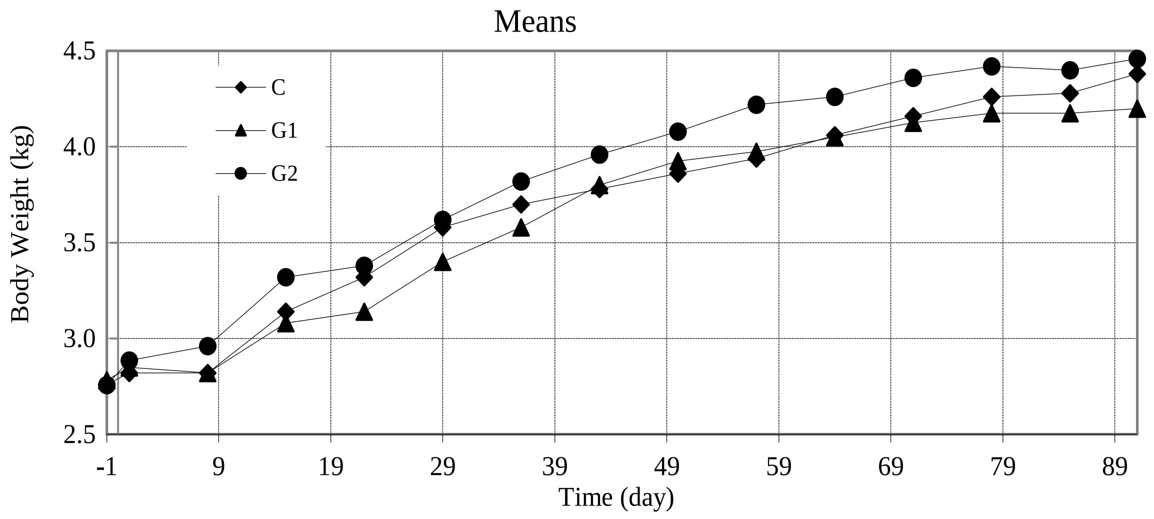

TABLE SET II

Body Temperature

Table 5: Body temperature - Group C (0 mg/animal) - Individual animal data (°C)

Day	1	8	15	22	29	36	43	50	57	64	71	78	85	91
**Anim.No.**														
**M1**	38.7	38.5	38.4	38.8	38.8	38.9	38.7	39.2	39.1	39.3	39.5	38.7	38.8	39.7
**M2**	38.9	38.7	38.4	38.7	39.0	38.8	38.7	38.6	38.9	38.9	38.7	38.7	38.9	38.3
**M3**	39.4	38.9	39.0	39.3	39.5	38.9	38.6	39.5	39.2	38.7	39.0	39.5	39.3	38.1
**M4**	39.1	39.2	38.9	38.6	39.2	38.7	39.0	39.1	39.4	39.6	38.7	39.2	38.8	38.9
**M5**	38.8	39.0	38.5	38.5	39.1	39.0	39.0	39.0	39.1	39.1	39.1	39.1	39.1	39.3

Table 6: Body temperature - Group G1 (720mg/animal) - Individual animal data (°C)

Day	1	8	15	22	29	36	42	43	50	57	64	71	78	85	91
**Anim.No.**															
**M6**	38.2	38.8	39.3	38.5	39.0	39.3		39.1	39.5	39.5	39.2	39.8	39.2	39.3	39.0
**M7**	38.5	n.t.	n.t.	n.t.	n.t.	n.t.	n.t.	n.t.	n.t.	n.t.	n.t.	n.t.	n.t.	n.t.	n.t.
**M8**	38.8	38.9	39.2	38.6	38.8	39.6		39.6	39.6	39.7	39.4	39.5	38.4	39.8	38.7
**M9**	38.3	38.7	38.8	38.7	39.1	38.6		39.8	39.6	39.2	38.9	39.5	38.8	39.3	39.0
**M10**	38.2	38.3	38.6	38.6	38.5	39.5	39.8	n.t.	n.t.	n.t.	n.t.	n.t.	n.t.	n.t.	n.t.
**M18**	39.0	38.6	39.6	39.0	38.9	38.8		39.1	38.7	38.8	39.3	38.8	39.1	38.6	39.1

Table 7: Body temperature - Group G2 (720mg/animal + vancomycin) - Individual animal data (°C)

Day	1	8	15	22	29	36	43	50	57	64	71	78	85	91
**Anim.No.**														
**M11**	38.9	n.t.	n.t.	n.t.	n.t.	n.t.	n.t.	n.t.	n.t.	n.t.	n.t.	n.t.	n.t.	n.t.
**M12**	38.7	38.6	39.0	38.4	39.5	38.9	39.2	39.1	39.3	39.4	39.1	39.3	39.3	38.9
**M13**	39.0	38.8	38.4	38.7	39.1	38.2	39.1	39.0	38.7	39.3	38.9	38.9	38.4	39.0
**M14**	38.6	38.9	38.8	38.5	39.1	38.8	39.2	39.0	38.9	39.0	39.1	39.1	38.8	38.4
**M15**	39.0	n.t.	n.t.	n.t.	n.t.	n.t.	n.t.	n.t.	n.t.	n.t.	n.t.	n.t.	n.t.	n.t.
**M16**	38.1	38.4	38.7	38.8	38.2	38.9	38.0	38.6	38.3	39.1	38.2	39.0	38.8	39.6
**M17**	38.9	38.6	39.2	38.7	39.2	39.3	39.3	39.2	39.4	39.2	39.2	39.0	39.0	39.7

Note: n.t. – not tested (deceased animal)

Table 8: Body temperature - mean, median and SD values

Group	Day	1	8	15	22	29	36	43	50	57	64	71	78	85	91
**C**															
	N	5	5	5	5	5	5	5	5	5	5	5	5	5	5
	Mean	39.0	38.9	38.6	38.8	39.1	38.9	38.8	39.1	39.1	39.1	39.0	39.0	39.0	38.9
	Median	38.9	38.9	38.5	38.7	39.1	38.9	38.7	39.1	39.1	39.1	39.0	39.1	38.9	38.9
	SD	0.3	0.3	0.3	0.3	0.3	0.1	0.2	0.3	0.2	0.3	0.3	0.3	0.2	0.7
**G1**								**	x						
	N	6	5	5	5	5	5	**4**	4	4	4	4	4	4	4
	Mean	38.5	38.7	39.1	38.7	38.9	39.2	**39.4**	39.4	39.3	39.2	39.4	38.9	39.3	39.0
	Median	38.4	38.7	39.2	38.6	38.9	39.3	**39.4**	39.6	39.4	39.3	39.5	39.0	39.3	39.0
	SD	0.3	0.2	0.4	0.2	0.2	0.4	**0.4**	0.4	0.4	0.2	0.4	0.4	0.5	0.2
**G2**								x				x			
	N	7	5	5	5	5	5	5	5	5	5	5	5	5	5
	Mean	38.7	38.7	38.8	38.6	39.0	38.8	39.0	39.0	38.9	39.2	38.9	39.1	38.9	39.1
	Median	38.9	38.6	38.8	38.7	39.1	38.9	39.2	39.0	38.9	39.2	39.1	39.0	38.8	39.0
	SD	0.3	0.2	0.3	0.2	0.5	0.4	0.5	0.2	0.4	0.2	0.4	0.2	0.3	0.5

Notes for Table 8:
Statistical evaluation by ANOVA p with Dunnett's MC test (parametric test for means) and Kruskal-Wallis test p with Dunn's MC test (non-parametric test for medians)
Statistically significant difference at the 95.0 % confidence level
** Statistically significant difference only between medians test groups G1 or G2 versus control group C
x Normality test not passed (for N > 4) - non-parametric test should be used for evaluation

GRAPH SET II

Body Temperature

Graph 2: Body Temperature values - Means

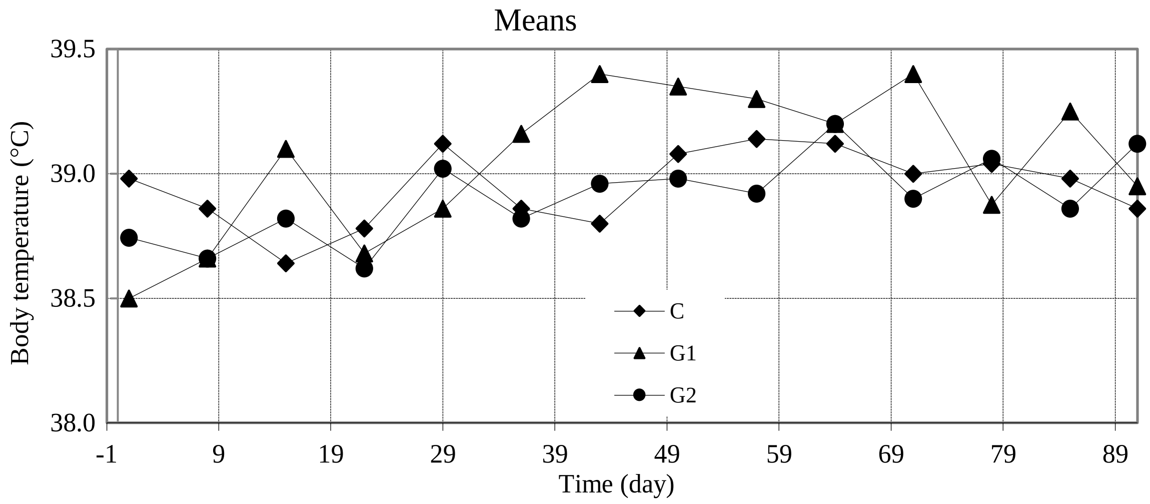

TABLE SET III

Food Consumption

Table 9: Food consumption - Group C (0 mg/animal) - Individual animal data(g)

Week	-1	1	2	3	4	5	6	7	8	9	10	11	12	13
Anim.No.														
M1	938	1040	1302	1224	1382	1246	1166	1212	1228	1162	1148	1162	1038	986
M2	1065	1405	1748	1462	1754	1865	1652	1676	1704	1716	1564	1610	1397	1382
M3	1002	1360	1644	1522	1680	1318	924	1078	1432	1524	1460	1464	1416	1362
M4	1008	912	1608	1250	1546	1332	1250	1462	1576	1512	1460	1396	1162	1018
M5	1072	1344	1755	1494	1754	1554	1326	1380	1072	1560	1502	1468	1246	1236

Table 10: Food consumption - Group G1 (720 mg/animal) - Individual animal data(g)

Week	-1	-1	1	2	3	4	5	6	7	8	9	10	11	12	13
Anim.No.															
M6	1245	256	1174	1564	1342	1570	1572	1556	1502	1430	1386	1388	1278	1116	1020
M7	1239	272	26	n.t.	n.t.	n.t.	n.t.	n.t.	n.t.	n.t.	n.t.	n.t.	n.t.	n.t.	n.t.
M8	1052	256	994	990	1004	1292	1220	1206	1216	1274	1370	1262	1278	1156	1088
M9	1088	246	1282	1748	1378	1554	1352	1308	1270	1166	1132	976	1104	1020	866
M10	1093	272	1012	1204	960	1254	1104	406	n.t.	n.t.	n.t.	n.t.	n.t.	n.t.	n.t.
M18	1138	-	1170	1578	1352	1520	1446	1372	1535	1412	1470	1450	1308	1228	1038

Table 11: Food consumption - Group G2 (720 mg/animal+vancomycin) - Individual animal data(g)

Week	-1	-1	-1	1	2	3	4	5	6	7	8	9	10	11	12	13
**Anim.No.**																
**M11**	1209	30	-	0	n.t.	n.t.	n.t.	n.t.	n.t.	n.t.	n.t.	n.t.	n.t.	n.t.	n.t.	n.t.
**M12**	1284	50	-	1376	1764	1546	1797	1602	1562	1502	1552	1499	1366	1028	646	770
**M13**	1068	22	-	1313	1328	1232	1516	1394	1444	1478	1574	1600	1570	1524	1264	1252
**M14**	1189	26	-	1172	1654	1414	1670	1562	1516	1462	1400	1328	1338	1356	1176	1128
**M15**	1298	34	-	0	n.t.	n.t.	n.t.	n.t.	n.t.	n.t.	n.t.	n.t.	n.t.	n.t.	n.t.	n.t.
**M16**	1174	1604	896	1220	1600	1386	1616	1666	1756	1664	1468	1644	1504	1294	1200	1078
**M17**	1218	1208	758	1392	1832	1644	1798	1614	1744	1676	1660	1563	1526	1324	1232	1190

Note: n.t. not tested (deceased animal)

Table 12: Food consumption - mean, median and SD values

Group	Week	-1	1	2	3	4	5	6	7	8	9	10	11	12	13
C															
	N	5	5	5	5	5	5	5	5	5	5	5	5	5	5
	Mean	1 017	1 212	1 611	1 390	1 623	1 463	1 264	1 362	1 402	1 495	1 427	1 420	1 252	1 197
	Median	1 008	1 344	1 644	1 462	1 680	1 332	1 250	1 380	1 432	1 524	1 460	1 464	1 246	1 236
	SD	54	221	184	142	159	253	264	230	256	203	162	164	160	187
G1		*			x								x		
	N	**6**	5	5	5	5	5	4	4	4	4	4	4	4	4
	Mean	**1 143**	1 126	1 417	1 207	1 438	1 339	1 361	1 381	1 321	1 340	1 269	1 242	1 130	1 003
	Median	**1 116**	1 170	1 564	1 342	1 520	1 352	1 340	1 386	1 343	1 378	1 325	1 278	1 136	1 029
	SD	**82**	121	310	207	152	184	147	161	124	145	210	93	87	96
G2		***						*						x	
	N	**7**	5	5	5	5	5	**5**	5	5	5	5	5	5	5
	Mean	**1 206**	1 295	1 636	1 444	1 679	1 568	**1 604**	1 556	1 531	1 527	1 461	1 305	1 104	1 084
	Median	**1 209**	1 313	1 654	1 414	1 670	1 602	**1 562**	1 502	1 552	1 563	1 504	1 324	1 200	1 128
	SD	**76**	96	194	158	121	104	**139**	105	100	123	103	179	258	187

Notes for Table 12:
Statistical evaluation by ANOVA p with Dunnett's MC test (parametric test for means) and Kruskal-Wallis test p with Dunn's MC test (non-parametric test for medians)
Statistically significant difference at the 95.0 % confidence level
* Statistically significant difference only between means test groups G1 or G2 versus control group C
*** Statistically significant difference between means and medians test groups G1 or G2 versus control group C
x Normality test not passed (for N > 4) - non-parametric test should be used for evaluation

GRAPH SET III

Food Consumption

Graph 3: Food Consumption values - Means

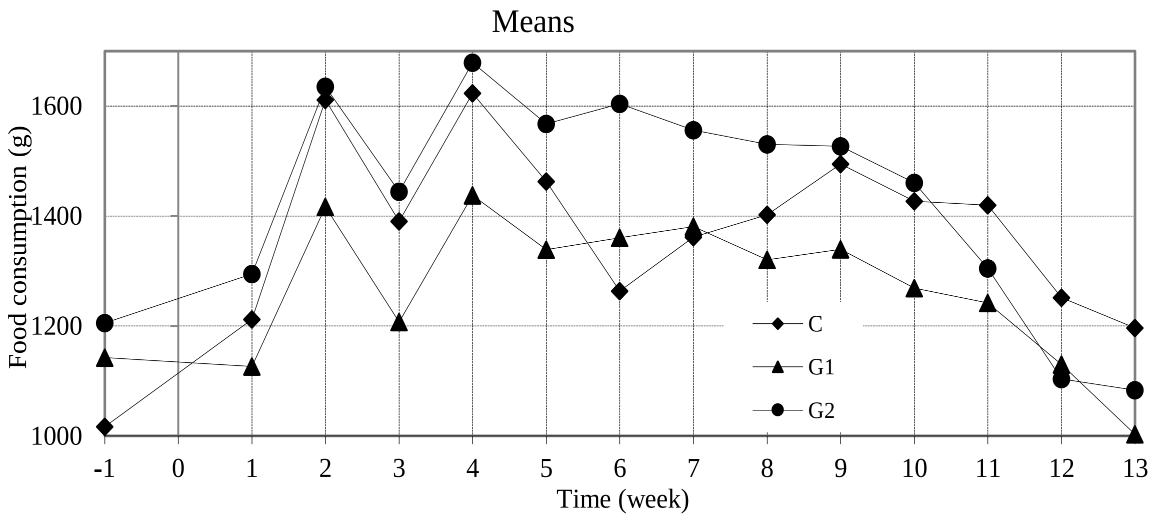

TABLE SET IV

Haematology

Table 13: Haematology - Group C (0 mg/animal) - Individual animal data

Exam. No.	Date	Anim. No.	WBC 10^9/l	LYM 10^9/l	MON 10^9/l	NEU 10^9/l	EOS 10^9/l	BAS 10^9/l	RBC 10^{12}/l	HGB g/l	HCT l/l	MCV fl	MCH pg	MCHC g/l	PLT 10^9/l	PT s	APTT s
1	05.03.2020	M1	5.5	2.26	0.11	2.86	0.06	0.22	6.58	149	0.453	69	22.6	329	249	7.2	40.7
		M2	10.1	4.04	0.10	5.25	0.51	0.20	5.53	123	0.374	68	22.3	329	366	7.2	46.7
		M3	11.2	4.48	0.56	5.94	0.00	0.22	6.02	138	0.419	70	23.0	330	418	7.4	43.5
		M4	7.6	2.43	0.30	4.26	0.23	0.38	6.46	130	0.408	63	20.2	319	388	7.0	42.1
		M5	5.6	2.07	0.06	3.30	0.11	0.06	6.00	124	0.382	64	20.6	324	547	7.0	46.3
2	17.03.2020	M1	8.3	3.07	0.50	4.23	0.00	0.50	6.58	145	0.444	68	22.0	326	335	7.2	34.2
		M2	9.8	3.23	0.10	6.17	0.20	0.10	5.32	116	0.352	66	21.8	329	509	7.6	37.2
		M3	10.8	3.46	1.51	5.51	0.00	0.32	6.26	141	0.434	69	22.4	324	563	7.6	43.5
		M4	9.4	3.48	0.38	5.45	0.00	0.09	6.32	127	0.396	63	20.0	320	536	7.5	41.2
		M5	6.7	2.41	0.34	3.48	0.27	0.20	6.42	128	0.408	64	20.0	315	574	7.4	54.2
3	31.03.2020	M1	7.3	3.58	0.15	3.43	0.00	0.15	6.91	152	0.470	68	22.1	325	264	7.0	31.0
		M2	6.3	3.97	0.19	1.70	0.00	0.44	6.44	139	0.423	66	21.5	328	337	7.1	47.1
		M3	8.4	5.54	0.08	2.60	0.00	0.17	6.40	144	0.443	69	22.5	326	366	7.0	45.8
		M4	8.0	4.00	0.48	3.28	0.08	0.16	7.69	153	0.479	62	20.0	321	460	6.7	41.6
		M5	9.2	3.31	1.01	4.78	0.09	0.00	7.03	141	0.442	63	20.1	320	518	6.7	55.3
4	02.06.2020	M1	7.8	3.90	0.39	3.20	0.08	0.23	7.02	159	0.481	68	22.7	331	194	7.3	31.3
		M2	7.3	4.16	0.66	2.12	0.37	0.00	6.34	143	0.429	68	22.5	333	273	7.3	34.4
		M3	8.1	4.78	0.49	2.84	0.00	0.00	6.69	153	0.458	68	22.9	335	310	7.3	42.7
		M4	11.0	4.29	0.77	5.72	0.11	0.11	7.72	164	0.497	64	21.2	329	324	7.2	43.8
		M5	6.1	2.81	0.12	2.99	0.18	0.00	7.26	157	0.471	65	21.7	335	329	7.2	45.4

Table 14: Haematology - Group G1 (720 mg/animal) - Individual animal data

Exam. No.	Date	Anim. No.	WBC 10^9/l	LYM 10^9/l	MON 10^9/l	NEU 10^9/l	EOS 10^9/l	BAS 10^9/l	RBC 10^{12}/l	HGB g/l	HCT l/l	MCV fl	MCH pg	MCHC g/l	PLT 10^9/l	PT s	APTT s
1	05.03.2020	M6	5.9	3.66	0.30	1.71	0.12	0.12	6.35	133	0.410	65	21.0	325	320	7.2	45.6
		M8	6.6	4.16	0.33	1.85	0.13	0.13	6.19	133	0.411	66	21.5	324	371	7.2	43.8
		M9	7.5	4.65	0.08	2.25	0.23	0.30	6.19	137	0.417	67	22.1	328	533	7.1	43.8
		M10	7.9	4.66	0.32	2.37	0.00	0.55	6.49	132	0.410	63	20.3	321	488	7.3	34.5
	26.03.2020	M18	9.6	5.86	0.58	3.07	0.00	0.10	6.47	141	0.421	65	21.7	334	451	7.3	46.6
2	19.03.2020	M6	6.9	4.83	0.21	1.38	0.48	0.00	5.51	112	0.358	65	20.4	313	504	7.3	39.6
		M8	7.7	4.24	0.39	3.00	0.08	0.00	5.87	123	0.386	66	21.0	319	487	6.8	48.8
		M9	8.1	3.89	0.57	3.08	0.08	0.49	5.77	122	0.377	65	21.1	323	632	7.0	46.0
		M10	9.5	3.14	0.29	5.51	0.29	0.29	6.93	131	0.432	62	18.9	304	967	7.0	51.9
	07.04.2020	M18	10.0	5.10	1.30	3.40	0.00	0.20	6.20	133	0.407	66	21.5	327	622	7.3	51.5
3	02.04.2020	M6	6.6	3.89	0.13	2.44	0.00	0.13	6.47	135	0.421	65	20.8	320	294	7.3	45.7
		M8	8.4	4.28	0.08	3.53	0.25	0.25	6.71	143	0.443	66	21.3	321	328	7.0	50.2
		M9	10.1	4.44	0.51	5.05	0.00	0.10	6.55	142	0.431	66	21.6	328	552	7.1	50.1
		M10	9.2	3.04	0.28	5.80	0.09	0.00	7.25	136	0.429	59	18.7	316	573	7.0	48.9
	21.04.2020	M18	11.8	5.43	0.35	5.19	0.47	0.35	6.61	144	0.437	66	21.8	330	455	6.9	51.6
4	04.06.2020	M6	8.7	4.52	0.61	3.39	0.17	0.00	6.58	146	0.437	66	22.2	334	225	7.3	49.3
		M8	5.9	3.36	0.12	2.42	0.00	0.00	6.56	147	0.440	67	22.4	334	311	6.9	43.8
		M9	13.1	7.34	0.39	5.37	0.00	0.00	6.61	148	0.443	67	22.4	334	376	6.8	43.2
		M10	n.t.	n.t.	n.t.	n.t.	n.t.	n.t.	n.t.	n.t.	n.t.	n.t.	n.t.	n.t.	n.t.	n.t.	n.t.
	23.06.2020	M18	10.0	2.90	0.70	6.20	0.00	0.20	6.68	150	0.462	69	22.4	324	406	7.2	44.1

Note: n.t. not tested (euthanized animal)

Table 15: Haematology - Group G2 (720 mg/animal + vancomycin) - Individual animal data

Exam. No.	Date	Anim. No.	WBC $10^9/l$	LYM $10^9/l$	MON $10^9/l$	NEU $10^9/l$	EOS $10^9/l$	BAS $10^9/l$	RBC $10^{12}/l$	HGB g/l	HCT l/l	MCV fl	MCH pg	MCHC g/l	PLT $10^9/l$	PT s	APTT s
1	05.03.2020	M12	7.1	4.76	0.28	1.92	0.07	0.07	6.30	138	0.424	67	21.9	326	503	6.9	29.6
		M13	7.1	3.91	0.28	2.70	0.00	0.21	6.42	140	0.431	67	21.9	326	394	7.2	34.2
		M14	4.2	2.27	0.17	1.60	0.00	0.17	6.78	148	0.450	66	21.8	328	231	7.7	32.2
	17.03.2020	M16	7.1	2.06	0.28	4.19	0.00	0.57	6.27	133	0.410	65	21.2	324	498	7.2	51.4
		M17	6.1	2.38	0.49	3.11	0.06	0.06	6.18	129	0.406	66	20.9	318	580	7.2	49.8
2	18.03.2020	M12	5.8	3.71	0.29	1.68	0.06	0.06	5.95	127	0.397	67	21.4	320	628	7.0	33.8
		M13	8.1	3.97	0.41	3.56	0.00	0.16	6.59	141	0.443	67	21.4	319	537	7.0	41.5
		M14	6.1	3.23	0.73	1.89	0.12	0.12	5.89	129	0.402	68	21.8	321	413	7.6	44.0
	07.04.2020	M16	4.8	2.21	0.29	2.21	0.05	0.05	6.77	141	0.437	65	20.9	324	567	7.3	50.4
		M17	6.4	3.14	0.32	2.88	0.06	0.00	6.36	132	0.409	64	20.8	323	568	7.0	45.2
3	01.04.2020	M12	5.6	3.58	0.11	1.68	0.17	0.06	6.68	144	0.448	67	21.5	321	474	7.1	28.5
		M13	6.7	3.15	0.27	2.88	0.00	0.40	7.36	157	0.484	66	21.4	325	438	7.0	45.9
		M14	6.0	2.88	0.54	2.34	0.06	0.18	7.24	162	0.491	68	22.4	330	304	7.3	43.8
	21.04.2020	M16	5.5	2.59	0.33	2.04	0.39	0.17	7.00	147	0.454	65	21.0	324	273	7.2	41.2
		M17	6.7	3.42	0.34	2.48	0.13	0.34	6.95	146	0.445	64	21.0	329	233	7.1	46.0
4	03.06.2020	M12	8.8	4.40	0.09	4.14	0.09	0.09	6.69	153	0.455	68	22.8	336	481	7.5	40.5
		M13	8.0	4.32	0.40	2.88	0.32	0.08	6.93	156	0.468	68	22.5	333	310	7.4	40.5
		M14	7.0	3.43	0.42	2.87	0.21	0.07	6.86	160	0.482	70	23.4	333	279	7.6	51.3
	23.06.2020	M16	7.4	5.18	0.15	1.92	0.07	0.07	7.40	162	0.507	68	21.9	320	241	7.5	43.8
		M17	7.9	2.69	0.16	4.98	0.08	0.00	7.01	158	0.482	69	22.5	327	332	7.2	40.6

Table 16: Haematology - Statistics, mean, median and SD values

Exam. No.	Group	Parameter	WBC $10^9/l$	LYM $10^9/l$	MON $10^9/l$	NEU $10^9/l$	EOS $10^9/l$	BAS $10^9/l$	RBC $10^{12}/l$	HGB g/l	HCT l/l	MCV fl	MCH pg	MCHC g/l	PLT $10^9/l$	PT s	APTT s
1	C																
		**N**	5	5	5	5	5	5	5	5	5	5	5	5	5	5	5
		**Mean**	8.00	3.056	0.226	4.322	0.180	0.216	6.118	132.8	0.4072	66.8	21.74	326.2	393.6	7.2	43.86
		**Median**	7.60	2.432	0.110	4.256	0.112	0.220	6.020	130.0	0.4080	68.0	22.30	329.0	388.0	7.2	43.50
		**SD**	2.59	1.118	0.210	1.289	0.200	0.115	0.418	10.8	0.0315	3.1	1.26	4.7	107.0	0.2	2.61
	G1					***					x						x
		**N**	5	5	5	**5**	5	5	5	5	5	5	5	5	5	5	5
		**Mean**	7.50	4.597	0.318	**2.250**	0.095	0.240	6.338	135.2	0.4138	65.2	21.32	326.4	432.6	7.2	42.86
		**Median**	7.50	4.650	0.316	**2.250**	0.118	0.132	6.350	133.0	0.4110	65.0	21.50	325.0	451.0	7.2	43.80
		**SD**	1.41	0.816	0.178	**0.534**	0.096	0.193	0.145	3.8	0.0050	1.5	0.69	4.9	86.5	0.1	4.83
	G2		x				x										
		**N**	5	5	5	5	5	5	5	5	5	5	5	5	5	5	5
		**Mean**	6.32	3.074	0.302	2.702	0.026	0.216	6.390	137.6	0.4242	66.2	21.54	324.4	441.2	7.2	39.44
		**Median**	7.10	2.379	0.284	2.698	0.000	0.168	6.300	138.0	0.4240	66.0	21.80	326.0	498.0	7.2	34.20
		**SD**	1.26	1.192	0.116	1.027	0.036	0.207	0.234	7.2	0.0176	0.8	0.46	3.8	134.8	0.3	10.33
2	C						x										
		**N**	5	5	5	5	5	5	5	5	5	5	5	5	5	5	5
		**Mean**	9.00	3.130	0.564	4.970	0.093	0.243	6.180	131.4	0.4068	66.0	21.24	322.8	503.4	7.5	42.06
		**Median**	9.40	3.234	0.376	5.452	0.000	0.201	6.320	128.0	0.4080	66.0	21.80	324.0	536.0	7.5	41.20
		**SD**	1.57	0.435	0.550	1.087	0.130	0.171	0.496	11.7	0.0362	2.5	1.15	5.4	97.5	0.2	7.67
	G1			*												*	
		**N**	5	**5**	5	5	5	5	5	5	5	5	5	5	5	**5**	5
		**Mean**	8.44	**4.238**	0.549	3.274	0.185	0.194	6.056	124.2	0.3920	64.8	20.58	317.2	642.4	**7.1**	47.56
		**Median**	8.10	**4.235**	0.385	3.078	0.081	0.200	5.870	123.0	0.3860	65.0	21.00	319.0	622.0	**7.0**	48.80
		**SD**	1.28	**0.780**	0.441	1.476	0.197	0.205	0.548	8.3	0.0285	1.6	1.02	9.0	193.1	**0.2**	5.04
	G2		***		x	***										x	
		**N**	**5**	5	5	**5**	5	5	5	5	5	5	5	5	5	5	5
		**Mean**	**6.24**	3.252	0.407	**2.445**	0.058	0.078	6.312	134.0	0.4176	66.2	21.26	321.4	542.6	7.2	42.98
		**Median**	**6.10**	3.233	0.320	**2.208**	0.058	0.058	6.360	132.0	0.4090	67.0	21.40	321.0	567.0	7.0	44.00
		**SD**	**1.20**	0.676	0.188	**0.772**	0.044	0.064	0.387	6.6	0.0210	1.6	0.41	2.1	79.6	0.3	6.07

To be continued

Table 16: Haematology (continued) - Statistics, mean, median and SD values

Exam. No.	Group	Parameter	WBC 10^9/l	LYM 10^9/l	MON 10^9/l	NEU 10^9/l	EOS 10^9/l	BAS 10^9/l	RBC 10^{12}/l	HGB g/l	HCT l/l	MCV fl	MCH pg	MCHC g/l	PLT 10^9/l	PT s	APTT s
3	C						x										
		N	5	5	5	5	5	5	5	5	5	5	5	5	5	5	5
		Mean	7.84	4.080	0.382	3.160	0.034	0.183	6.894	145.8	0.4514	65.6	21.24	324.0	389.0	6.9	44.16
		Median	8.00	3.969	0.189	3.280	0.000	0.160	6.910	144.0	0.4430	66.0	21.50	325.0	366.0	7.0	45.80
		SD	1.10	0.867	0.383	1.136	0.047	0.160	0.525	6.4	0.0228	3.0	1.14	3.4	100.7	0.2	8.88
	G1											x					
		N	5	5	5	5	5	5	5	5	5	5	5	5	5	5	5
		Mean	9.22	4.217	0.270	4.402	0.163	0.168	6.718	140.0	0.4322	64.4	20.84	323.0	440.4	7.1	49.30
		Median	9.20	4.284	0.276	5.050	0.092	0.132	6.610	142.0	0.4310	66.0	21.30	321.0	455.0	7.0	50.10
		SD	1.93	0.869	0.170	1.378	0.201	0.138	0.310	4.2	0.0083	3.0	1.25	5.8	126.8	0.2	2.23
	G2										x						x
		N	5	5	5	5	5	5	5	5	5	5	5	5	5	5	5
		Mean	6.10	3.123	0.317	2.283	0.149	0.228	7.046	151.2	0.4644	66.0	21.46	325.8	344.4	7.1	41.08
		Median	6.00	3.149	0.330	2.340	0.134	0.180	7.000	147.0	0.4540	66.0	21.40	325.0	304.0	7.1	43.80
		SD	0.58	0.403	0.154	0.454	0.147	0.139	0.265	7.9	0.0215	1.6	0.57	3.7	105.7	0.1	7.30
4	C							x				x				x	
		N	5	5	5	5	5	5	5	5	5	5	5	5	5	5	5
		Mean	8.06	3.987	0.485	3.372	0.147	0.069	7.006	155.2	0.4672	66.6	22.20	332.6	286.0	7.3	39.52
		Median	7.80	4.161	0.486	2.989	0.110	0.000	7.020	157.0	0.4710	68.0	22.50	333.0	310.0	7.3	42.70
		SD	1.81	0.733	0.251	1.374	0.138	0.104	0.529	7.9	0.0257	1.9	0.72	2.6	55.9	0.1	6.26
	G1						x	x			x		x	x			x
		N	4	4	4	4	4	4	4	4	4	4	4	4	4	4	4
		Mean	9.43	4.531	0.455	4.346	0.044	0.050	6.608	147.8	0.4455	67.3	22.35	331.5	329.5	7.1	45.10
		Median	9.35	3.944	0.501	4.382	0.000	0.000	6.595	147.5	0.4415	67.0	22.40	334.0	343.5	7.1	43.95
		SD	2.99	1.991	0.259	1.743	0.087	0.100	0.053	1.7	0.0113	1.3	0.10	5.0	80.2	0.2	2.82
	G2							x				x					x
		N	5	5	5	5	5	5	5	5	5	5	5	5	5	5	5
		Mean	7.82	4.003	0.243	3.357	0.154	0.062	6.978	157.8	0.4788	68.6	22.62	329.8	328.6	7.4	43.34
		Median	7.90	4.320	0.158	2.880	0.088	0.074	6.930	158.0	0.4820	68.0	22.50	333.0	310.0	7.5	40.60
		SD	0.68	0.963	0.155	1.199	0.108	0.036	0.264	3.5	0.0194	0.9	0.54	6.4	91.8	0.2	4.67

Notes for Table 16:
Statistical evaluation by ANOVA p with Dunnett's MC test (parametric test for means) and Kruskal-Wallis test p with Dunn's MC test (non-parametric test for medians)
Statistically significant difference at the 95.0 % confidence level
* Statistically significant difference only between means test groups G1 or G2 versus control group C
*** Statistically significant difference between means and medians test groups G1 or G2 versus control group C
x Normality test not passed (for N > 4) - non-parametric test should be used for evaluation

TABLE SET V

Clinical Chemistry

Table 17: Clinical Chemistry - Group C (0 mg/animal) - Individual animal data

Exam. No.	Date	Anim. No.	Glu mmol/l	Na mmol/l	K mmol/l	Cl mmo/l	Ca mmol/l	P mmo/l	Urea mmol/l	Crea µmol/l	Bil µmol/l	LDH µkat/l	ALT µkat/l	AST µkat/l	GGT µkat/l	ALP µkat/l	Chol mmol/l	TGC mmol/l	TP g/l	Alb g/l	Glo g/l	Alb/Glo ratio
1	05.03.2020	M1	5.91	140	4.2	98	3.36	2.54	6.8	67	5	1.30	1.56	1.28	0.32	5.24	1.57	0.85	62	20	42	0.48
		M2	6.10	150	4.0	110	3.42	2.72	5.9	58	3	2.48	1.79	0.91	0.32	4.73	1.61	1.44	74	19	55	0.35
		M3	5.98	141	4.0	101	3.31	2.42	7.4	70	3	1.82	1.90	0.74	0.28	5.56	< 1.30	4.50	65	19	46	0.41
		M4	5.65	135	4.8	94	3.28	2.68	7.3	62	5	1.75	0.92	0.41	0.32	4.95	1.42	1.45	69	18	51	0.35
		M5	4.89	130	3.9	89	3.13	2.41	6.2	64	3	1.47	0.73	0.72	0.24	3.98	1.33	1.43	63	18	45	0.40
2	17.03.2020	M1	6.40	146	4.2	106	3.51	2.32	6.9	84	2	1.18	1.05	0.56	0.30	3.05	1.50	1.25	64	19	45	0.42
		M2	6.33	142	3.6	103	3.48	2.37	5.9	65	2	1.44	1.48	0.60	0.29	3.01	< 1.30	1.08	68	18	50	0.36
		M3	6.37	141	4.2	104	3.50	2.27	6.7	77	2	0.86	1.44	0.58	0.32	2.81	< 1.30	1.59	64	18	46	0.39
		M4	5.60	138	4.2	100	3.42	2.24	6.4	59	3	1.05	0.98	0.56	0.29	2.01	< 1.30	1.25	64	16	48	0.33
		M5	5.98	140	4.4	97	3.67	2.42	6.3	86	3	1.03	0.65	0.55	0.25	3.03	1.33	0.96	71	20	51	0.39
3	31.03.2020	M1	5.70	134	3.7	98	3.48	2.21	6.6	83	< 2	0.72	1.32	0.44	0.30	4.36	1.41	0.86	68	23	45	0.51
		M2	5.80	139	4.0	98	3.43	2.07	5.7	76	< 2	0.79	1.79	0.56	0.30	3.89	< 1.30	1.07	67	21	46	0.46
		M3	5.64	143	3.6	106	3.43	2.27	7.8	73	< 2	0.71	1.40	0.43	0.33	3.07	< 1.30	1.34	68	21	47	0.45
		M4	5.84	140	4.6	98	3.50	2.23	7.1	81	< 2	0.99	0.95	0.49	0.29	2.94	< 1.30	0.88	75	22	53	0.42
		M5	6.03	143	4.6	102	3.62	2.58	6.6	89	3	1.03	0.59	0.51	0.26	3.69	1.35	0.96	73	22	51	0.43
4	02.06.2020	M1	6.24	144	4.5	106	3.60	1.67	8.1	110	4	0.94	1.32	0.85	0.34	1.70	< 1.30	0.53	70	23	47	0.49
		M2	6.83	143	4.3	105	3.47	1.65	6.6	84	4	2.15	2.57	1.83	0.31	1.86	< 1.30	0.58	72	23	49	0.47
		M3	6.64	149	4.3	110	3.69	1.64	9.5	123	2	1.60	1.36	1.10	0.34	1.66	< 1.30	1.00	86	25	61	0.41
		M4	6.70	145	4.5	102	3.68	1.76	7.1	110	3	1.44	0.92	0.59	0.31	1.73	< 1.30	0.35	88	26	62	0.42
		M5	7.53	154	4.7	111	3.63	2.18	7.2	128	5	4.71	0.57	0.55	0.25	1.66	< 1.30	0.68	90	25	65	0.38

Table 18: Clinical Chemistry - Group G1 (720 mg/animal) - Individual animal data

Exam. No.	Date	Anim. No.	Glu mmol/l	Na mmol/l	K mmol/l	Cl mmo/l	Ca mmol/l	P mmo/l	Urea mmol/l	Crea µmol/l	Bil µmol/l	LDH µkat/l	ALT µkat/l	AST µkat/l	GGT µkat/l	ALP µkat/l	Chol mmol/l	TGC mmol/l	TP g/l	Alb g/l	Glo g/l	Alb/Glo ratio
1	05.03.2020	M6	5.90	143	4.8	102	3.39	2.43	7.7	64	2	0.72	0.65	0.34	0.42	6.79	1.43	0.97	64	18	46	0.39
		M8	5.35	134	4.4	97	3.12	2.18	5.9	68	4	1.87	1.23	0.43	0.31	3.33	1.58	2.42	59	16	43	0.37
		M9	6.77	146	4.4	106	3.52	2.17	5.3	82	2	0.96	0.56	0.36	0.28	5.41	1.31	0.80	69	20	49	0.41
		M10	6.69	140	4.6	99	3.33	2.73	4.0	96	5	1.78	1.29	0.40	0.33	6.46	2.39	0.76	63	17	46	0.37
	26.03.2020	M18	5.94	142	4.8	105	3.41	2.51	5.6	71	< 2	1.87	1.12	0.33	0.32	3.46	< 1.30	0.69	67	20	47	0.43
2	19.03.2020	M6	6.12	143	3.9	106	3.45	2.24	8.2	70	2	0.53	0.83	0.34	0.37	3.06	< 1.30	0.78	63	18	45	0.40
		M8	6.29	140	3.9	99	3.56	2.14	9.0	82	2	0.76	1.04	0.32	0.33	2.24	1.47	1.50	64	17	47	0.36
		M9	6.40	140	4.2	102	3.65	2.07	7.0	84	2	0.83	1.23	0.41	0.26	3.99	1.31	1.11	68	19	49	0.39
		M10	5.67	141	4.1	103	3.51	2.16	6.8	64	7	2.34	0.96	0.24	0.30	2.68	1.63	0.66	66	17	49	0.35
	07.04.2020	M18	6.05	143	4.3	106	3.53	2.24	6.8	76	2	0.78	1.28	0.36	0.31	2.69	< 1.30	0.75	68	20	48	0.42
3	02.04.2020	M6	5.43	141	4.0	103	3.39	2.27	8.1	71	< 2	0.77	1.27	0.49	0.34	4.37	< 1.30	0.66	67	21	46	0.46
		M8	5.43	141	4.0	100	3.42	2.40	11.6	131	4	1.50	1.12	0.43	0.33	4.28	1.47	2.03	71	19	52	0.37
		M9	5.05	144	4.0	106	3.58	2.18	6.8	87	< 2	1.26	0.87	0.51	0.24	4.15	< 1.30	0.83	77	21	56	0.38
		M10	5.34	146	4.0	109	3.46	2.09	7.4	70	2	1.79	0.95	0.27	0.27	2.36	1.68	0.61	64	18	46	0.39
	21.04.2020	M18	6.56	142	5.1	101	3.46	2.27	6.5	81	< 2	0.91	1.32	0.40	0.33	3.48	< 1.30	0.57	67	20	47	0.43
4	04.06.2020	M6	6.33	146	3.9	106	3.58	1.59	8.1	97	3	0.91	1.00	0.50	0.39	1.65	< 1.30	0.43	73	23	50	0.46
		M8	5.97	141	4.1	104	3.55	1.52	9.2	125	5	1.79	1.26	0.43	0.34	2.12	< 1.30	0.65	76	22	54	0.41
		M9	6.19	142	4.1	104	3.57	1.60	9.8	135	3	1.14	0.57	0.35	0.30	1.53	< 1.30	0.61	83	21	62	0.34
		M10	n.t.	n.t.	n.t.	n.t.	n.t.	n.t.	n.t.	n.t.	n.t.	n.t.	n.t.	n.t.	n.t.	n.t.	n.t.	n.t.	n.t.	n.t.	n.t.	n.t.
	23.06.2020	M18	6.49	145	4.3	99	3.61	1.71	6.7	95	< 2	1.31	1.72	0.33	0.37	3.39	< 1.30	2.53	82	24	58	0.41

Note: n.t. not tested (euthanized animal)

Table 19: Clinical Chemistry - Group G2 (720 mg/animal + vancomycin) - Individual animal data

Exam. No.	Date	Anim. No.	Glu mmol/l	Na mmol/l	K mmol/l	Cl mmo/l	Ca mmol/l	P mmo/l	Urea mmol/l	Crea µmol/l	Bil µmol/l	LDH µkat/l	ALT µkat/l	AST µkat/l	GGT µkat/l	ALP µkat/l	Chol mmol/l	TGC mmol/l	TP g/l	Alb g/l	Glo g/l	Alb/Glo ratio
**1**	**05.03.2020**	**M12**	6.60	146	5.6	107	3.74	2.48	4.8	78	8	1.31	1.63	0.93	0.31	3.80	1.89	0.60	63	18	45	0.40
		**M13**	6.22	142	5.0	102	3.64	2.88	6.9	86	4	1.07	1.74	0.39	0.41	4.20	1.92	1.11	70	20	50	0.40
		**M14**	5.93	140	4.5	99	3.46	2.85	8.9	77	3	1.26	0.88	0.38	0.32	5.81	1.35	1.52	69	20	49	0.41
	**17.03.2020**	**M16**	6.00	141	4.1	103	3.43	2.24	5.9	78	3	1.22	1.41	0.54	0.30	2.44	1.76	1.69	68	19	49	0.39
		**M17**	5.93	139	4.3	100	3.43	2.10	4.9	62	2	0.90	1.12	0.87	0.31	3.65	< 1.30	0.84	71	19	52	0.37
**2**	**18.03.2020**	**M12**	6.64	146	4.5	106	3.72	2.15	5.9	83	4	1.01	1.30	0.64	0.29	2.70	1.31	0.75	67	19	48	0.40
		**M13**	6.32	142	4.5	102	3.74	2.10	6.7	73	2	0.99	1.04	0.52	0.36	2.85	< 1.30	0.97	70	20	50	0.40
		**M14**	6.37	139	4.1	100	3.50	2.10	7.8	74	2	0.98	0.73	0.25	0.28	2.83	< 1.30	1.04	63	18	45	0.40
	**07.04.2020**	**M16**	5.62	142	3.9	102	3.43	2.32	8.2	80	2	0.59	1.13	0.34	0.27	1.75	1.72	1.00	67	20	47	0.43
		**M17**	5.72	143	4.5	104	3.45	2.34	6.4	81	3	0.88	0.81	0.45	0.29	2.49	1.33	1.01	69	19	50	0.38
**3**	**01.04.2020**	**M12**	6.25	149	4.3	112	3.64	2.11	8.6	79	3	0.84	1.55	0.84	0.30	3.43	1.77	0.91	69	19	50	0.38
		**M13**	5.96	147	4.4	109	3.68	2.14	6.8	83	2	1.06	1.70	0.62	0.36	3.63	1.51	0.86	74	22	52	0.42
		**M14**	5.78	140	4.3	97	3.61	2.43	9.1	90	3	0.79	0.79	0.42	0.24	5.80	< 1.30	1.17	75	24	51	0.47
	**21.04.2020**	**M16**	5.89	141	4.3	101	3.44	2.03	6.9	90	< 2	0.61	1.24	0.27	0.34	1.95	< 1.30	0.79	60	19	41	0.46
		**M17**	5.89	140	5.3	99	3.50	2.06	6.2	89	3	1.25	0.84	0.59	0.35	3.53	< 1.30	0.70	73	22	51	0.43
**4**	**03.06.2020**	**M12**	6.72	145	3.8	107	3.53	1.19	4.6	100	3	0.76	1.11	0.38	0.28	0.83	< 1.30	0.53	68	20	48	0.42
		**M13**	6.62	143	4.4	102	3.58	1.81	7.3	94	3	1.09	1.61	0.63	0.33	2.35	< 1.30	0.67	72	23	49	0.47
		**M14**	6.47	141	3.8	101	3.44	1.68	7.6	90	2	1.12	0.52	0.25	0.25	2.07	< 1.30	0.64	76	24	52	0.46
	**23.06.2020**	**M16**	7.00	148	4.6	108	3.61	1.78	8.1	125	4	1.02	1.44	0.36	0.33	1.09	< 1.30	0.55	75	23	52	0.44
		**M17**	6.69	149	4.5	107	3.63	1.63	6.2	97	5	1.67	1.91	0.72	0.32	3.15	< 1.30	0.46	82	26	56	0.46

Table 20: Clinical chemistry- Statistics, mean, median and SD values

Exam. No.	Group	Parameter	Glu mmol/l	Na mmol/l	K mmol/l	Cl mmol/l	Ca mmol/l	P mmol/l	Urea mmol/l	Crea µmol/l	Bil µmol/l	LDH µkat/l	ALT µkat/l	AST µkat/l	GGT µkat/l	ALP µkat/l	Chol mmol/l	TGC mmol/l	TP g/l	Alb g/l	Glo g/l	Alb/Glo ratio
											x			+	x		x	x				
		N	5	5	5	5	5	5	5	5	5	5	5	5	5	5	5	5	5	5	5	5
	C	Mean	5.706	139.2	4.18	98.4	3.300	2.554	6.72	64.2	3.8	1.764	1.380	0.812	0.296	4.892	1.186	1.934	66.6	18.8	47.8	0.398
		Median	5.910	140.0	4.00	98.0	3.310	2.540	6.80	64.0	3.0	1.750	1.560	0.740	0.320	4.950	1.420	1.440	65.0	19.0	46.0	0.400
		SD	0.485	7.5	0.36	7.9	0.109	0.143	0.66	4.6	1.1	0.452	0.526	0.318	0.036	0.598	0.673	1.457	4.9	0.8	5.2	0.053
														***				x				
		N	5	5	5	5	5	5	5	5	5	5	5	**5**	5	5	5	5	5	5	5	5
1	G1	Mean	6.130	141.0	4.60	101.8	3.354	2.404	5.70	76.2	2.6	1.440	0.970	**0.372**	0.332	5.090	1.342	1.128	64.4	18.2	46.2	0.393
		Median	5.940	142.0	4.60	102.0	3.390	2.430	5.60	71.0	2.0	1.780	1.120	**0.360**	0.320	5.410	1.430	0.800	64.0	18.0	46.0	0.391
		SD	0.596	4.5	0.20	3.8	0.148	0.236	1.33	12.9	1.9	0.555	0.340	**0.042**	0.053	1.630	0.861	0.730	3.8	1.8	2.2	0.024
							***								x							
		N	5	5	5	5	**5**	5	5	5	5	5	5	5	5	5	5	5	5	5	5	5
	G2	Mean	6.136	141.6	4.70	102.2	**3.540**	2.510	6.28	76.2	4.0	1.152	1.356	0.622	0.330	3.980	1.384	1.152	68.2	19.2	49.0	0.392
		Median	6.000	141.0	4.50	102.0	**3.460**	2.480	5.90	78.0	3.0	1.220	1.410	0.540	0.310	3.800	1.760	1.110	69.0	19.0	49.0	0.400
		SD	0.285	2.7	0.60	3.1	**0.142**	0.352	1.69	8.7	2.3	0.167	0.356	0.262	0.045	1.216	0.806	0.455	3.1	0.8	2.5	0.017
					x						x			+		x	x		x			
		N	5	5	5	5	5	5	5	5	5	5	5	5	5	5	5	5	5	5	5	5
	C	Mean	6.136	141.4	4.12	102.0	3.516	2.324	6.44	74.2	2.4	1.112	1.120	0.570	0.290	2.782	0.566	1.226	66.2	18.2	48.0	0.380
		Median	6.330	141.0	4.20	103.0	3.500	2.320	6.40	77.0	2.0	1.050	1.050	0.560	0.290	3.010	0.000	1.250	64.0	18.0	48.0	0.391
		SD	0.344	3.0	0.30	3.5	0.093	0.073	0.38	11.8	0.5	0.216	0.345	0.020	0.025	0.442	0.777	0.238	3.2	1.5	2.5	0.034
								*			x	x		***								
		N	5	5	5	5	5	**5**	5	5	5	5	5	**5**	5	5	5	5	5	5	5	5
2	G1	Mean	6.106	141.4	4.08	103.2	3.540	**2.170**	7.56	75.2	3.0	1.048	1.068	**0.334**	0.314	2.932	0.882	0.960	65.8	18.2	47.6	0.383
		Median	6.120	141.0	4.10	103.0	3.530	**2.160**	7.00	76.0	2.0	0.780	1.040	**0.340**	0.310	2.690	1.310	0.780	66.0	18.0	48.0	0.388
		SD	0.280	1.5	0.18	2.9	0.073	**0.072**	0.99	8.3	2.2	0.731	0.187	**0.062**	0.040	0.659	0.813	0.347	2.3	1.3	1.7	0.028
					x						x	x			x			x				
		N	5	5	5	5	5	5	5	5	5	5	5	5	5	5	5	5	5	5	5	5
	G2	Mean	6.134	142.4	4.30	102.8	3.568	2.202	7.00	78.2	2.6	0.890	1.002	0.440	0.298	2.524	0.872	0.954	67.2	19.2	48.0	0.400
		Median	6.320	142.0	4.50	102.0	3.500	2.150	6.70	80.0	2.0	0.980	1.040	0.450	0.290	2.700	1.310	1.000	67.0	19.0	48.0	0.400
		SD	0.442	2.5	0.28	2.3	0.150	0.119	0.97	4.4	0.9	0.175	0.233	0.152	0.036	0.456	0.813	0.117	2.7	0.8	2.1	0.016

To be continued

Table 20: Clinical chemistry (continued) - Statistics, mean, median and SD values

Exam. No.	Group	Parameter	Glu mmol/l	Na mmol/l	K mmol/l	Cl mmol/l	Ca mmol/l	P mmol/l	Urea mmol/l	Crea µmol/l	Bil µmol/l	LDH µkat/l	ALT µkat/l	AST µkat/l	GGT µkat/l	ALP µkat/l	Chol mmol/l	TGC mmol/l	TP g/l	Alb g/l	Glo g/l	Alb/Glo ratio
3	C					x					x						x					
		N	5	5	5	5	5	5	5	5	5	5	5	5	5	5	5	5	5	5	5	5
		Mean	5.802	139.8	4.10	100.4	3.492	2.272	6.76	80.4	0.6	0.848	1.210	0.486	0.296	3.590	0.552	1.022	70.2	21.8	48.4	0.452
		Median	5.800	140.0	4.00	98.0	3.480	2.230	6.60	81.0	0.0	0.790	1.320	0.490	0.300	3.690	0.000	0.960	68.0	22.0	47.0	0.447
		SD	0.150	3.7	0.48	3.6	0.078	0.188	0.77	6.2	1.3	0.152	0.457	0.053	0.025	0.589	0.756	0.196	3.6	0.8	3.4	0.036
	G1				x						x						x	x				
		N	5	5	5	5	5	5	5	5	5	5	5	5	5	5	5	5	5	5	5	5
		Mean	5.562	142.8	4.22	103.8	3.462	2.242	8.08	88.0	1.2	1.246	1.106	0.420	0.302	3.728	0.630	0.940	69.2	19.8	49.4	0.403
		Median	5.430	142.0	4.00	103.0	3.460	2.270	7.40	81.0	0.0	1.260	1.120	0.430	0.330	4.150	0.000	0.660	67.0	20.0	47.0	0.391
		SD	0.579	2.2	0.49	3.7	0.072	0.116	2.06	25.1	1.8	0.418	0.196	0.095	0.044	0.841	0.866	0.617	5.0	1.3	4.4	0.038
	G2				x						x						x				x	
		N	5	5	5	5	5	5	5	5	5	5	5	5	5	5	5	5	5	5	5	5
		Mean	5.954	143.4	4.52	103.6	3.574	2.154	7.52	86.2	2.2	0.910	1.224	0.548	0.318	3.668	0.656	0.886	70.2	21.2	49.0	0.434
		Median	5.890	141.0	4.30	101.0	3.610	2.110	6.90	89.0	3.0	0.840	1.240	0.590	0.340	3.530	0.000	0.860	73.0	22.0	51.0	0.431
		SD	0.178	4.3	0.44	6.5	0.100	0.160	1.26	5.0	1.3	0.249	0.409	0.216	0.049	1.376	0.903	0.177	6.1	2.2	4.5	0.036
4	C							x									#					
		N	5	5	5	5	5	5	5	5	5	5	5	5	5	5	5	5	5	5	5	5
		Mean	6.788	147.0	4.46	106.8	3.614	1.780	7.70	111.0	3.6	2.168	1.348	0.984	0.310	1.722	0.000	0.628	81.2	24.4	56.8	0.435
		Median	6.700	145.0	4.50	106.0	3.630	1.670	7.20	110.0	4.0	1.600	1.320	0.850	0.310	1.700	0.000	0.580	86.0	25.0	61.0	0.419
		SD	0.470	4.5	0.17	3.7	0.088	0.229	1.14	17.1	1.1	1.485	0.755	0.522	0.037	0.083	0.000	0.240	9.4	1.3	8.2	0.043
	G1																#	x				
		N	4	4	4	4	4	4	4	4	4	4	4	4	4	4	4	4	4	4	4	4
		Mean	6.245	143.5	4.10	103.3	3.578	1.605	8.45	113.0	2.8	1.288	1.138	0.403	0.350	2.173	0.000	1.055	78.5	22.5	56.0	0.405
		Median	6.260	143.5	4.10	104.0	3.575	1.595	8.65	111.0	3.0	1.225	1.130	0.390	0.355	1.885	0.000	0.630	79.0	22.5	56.0	0.411
		SD	0.221	2.4	0.16	3.0	0.025	0.079	1.36	20.1	2.1	0.373	0.481	0.078	0.039	0.851	0.000	0.988	4.8	1.3	5.2	0.050
	G2																#					
		N	5	5	5	5	5	5	5	5	5	5	5	5	5	5	5	5	5	5	5	5
		Mean	6.700	145.2	4.22	105.0	3.558	1.618	6.76	101.2	3.4	1.132	1.318	0.468	0.302	1.898	0.000	0.570	74.6	23.2	51.4	0.451
		Median	6.690	145.0	4.40	107.0	3.580	1.680	7.30	97.0	3.0	1.090	1.440	0.380	0.320	2.070	0.000	0.550	75.0	23.0	52.0	0.462
		SD	0.194	3.3	0.39	3.2	0.076	0.250	1.39	13.8	1.1	0.333	0.532	0.198	0.036	0.948	0.000	0.085	5.2	2.2	3.1	0.022

Notes for Table 20:
Values for parameter Bil < 2 and Chol < 1.30 were replaced by zero.
Statistical evaluation by ANOVA p with Tukey's MC test (parametric test for means) and Kruskal-Wallis test p with Dunn's MC test (non-parametric test for medians)
Statistically significant difference at the 95.0 % confidence level
* Statistically significant difference only between means test groups G1 or G2 versus control group C
*** Statistically significant difference between means and medians test groups G1 or G2 versus control group C
+ Statistically significant difference among standard deviations (F test) (for N > 4) - non-parametric test was used for evaluation
x Normality test not passed (for N > 4) - non-parametric test was used for evaluation
No variability in one of the columns

TABLE SET VI

Urinalysis

Table 21: Urinalysis - Group C (0 mg/animal) - Individual animal data

Exam. No.	Date	Anim. No.	Bil µmol/l	Ubg µmol/l	Ket mmol/l	Glu mmol/l	Pro g/l	Ery Ery/µl	pH	Nit	Leu Leu/µl	SG kg/l	Color	Turbidity	Volume ml
1	08.06.2020	M1	35	norm	1.0	norm	neg	neg	7.5	neg	neg	1.005	dark yellow	marked	52.0
		M2	35	norm	1.0	norm	0.3	neg	9.0	neg	neg	1.000	yellow	marked	20.0
		M3	neg	norm	neg	norm	neg	neg	8.0	neg	neg	1.005	yellow	marked	58.0
		M4	70	norm	1.0	norm	neg	neg	8.0	neg	neg	1.005	yellow	marked	13.0
		M5	17	norm	1.0	norm	0.3	neg	9.0	neg	neg	1.000	yellow	marked	22.0

Table 22: Urinalysis - Group G1 (720 mg/animal) - Individual animal data

Exam. No.	Date	Anim. No.	Bil µmol/l	Ubg µmol/l	Ket mmol/l	Glu mmol/l	Pro g/l	Ery Ery/µl	pH	Nit	Leu Leu/µl	SG kg/l	Color	Turbidity	Volume ml
1	10.06.2020	M6	neg	norm	1.0	norm	neg	neg	7.5	neg	neg	1.010	yellow	marked	10.0
		M8	neg	norm	1.0	norm	0.3	50	8.0	neg	neg	1.005	yellow	marked	4.0
		M9	neg	norm	neg	norm	neg	neg	8.0	neg	neg	1.000	yellow	marked	11.0
		M10	n.t.	n.t.	n.t.	n.t.	n.t.	n.t.	n.t.	n.t.	n.t.	n.t.	n.t.	n.t.	n.t.
	29.06.2020	M18	70	35	1.0	norm	0.3	neg	8.0	neg	neg	1.005	dark yellow	slight	12.0

Note: n.t. not tested (euthanized animal)

Table 23: Urinalysis - Group G2 (720 mg/animal + vancomycin) - Individual animal data

Exam. No.	Date	Anim. No.	Bil µmol/l	Ubg µmol/l	Ket mmol/l	Glu mmol/l	Pro g/l	Ery Ery/µl	pH	Nit	Leu Leu/µl	SG kg/l	Color	Turbidity	Volume ml
1	09.06.2020	M12	70	70	1.0	norm	1.0	neg	8.0	neg	neg	1.005	yellow	marked	6.0
		M13	70	140	1.0	norm	5.0	neg	8.0	neg	neg	1.005	yellow	marked	3.0
		M14	70	140	1.0	norm	1.0	neg	8.0	neg	neg	1.015	yellow	marked	25.0
	29.06.2020	M16	70	70	2.5	norm	1.0	neg	7.5	neg	25	1.005	yellow	marked	5.0
		M17	17	norm	1.0	norm	0.3	neg	8.0	neg	neg	1.015	yellow	marked	40.0

TABLE SET VII

Organ Weight

Table 24: Absolute Organ Weight (g) – Group C (0 mg/animal) - Individual animal data

Anim. No.	Body weight (kg)	Liver	Kidneys	Spleen	Brain	Heart	Thymus	Adrenals	Thyroid gland	Testes	Prostate	Epididym.
**M1**	4.2	128.41	21.32	1.591	11.52	15.84	2.730	0.336	0.453	7.46	2.133	3.93
**M2**	5.0	165.31	30.26	2.062	9.10	12.57	2.928	0.514	0.601	8.13	2.550	4.65
**M3**	4.4	140.69	26.62	2.391	10.53	15.86	3.110	0.387	0.315	8.50	2.628	3.82
**M4**	4.2	145.51	27.19	3.010	10.04	16.79	4.800	0.476	0.339	7.59	1.794	3.90
**M5**	4.1	163.17	24.38	1.345	9.20	15.95	2.622	0.468	0.370	9.54	2.368	4.69

Table 25: Absolute Organ Weight (g) – Group G1 (720 mg/animal) - Individual animal data

Anim. No.	Body weight (kg)	Liver	Kidneys	Spleen	Brain	Heart	Thymus	Adrenals	Thyroid gland	Testes	Prostate	Epididym.
**M6**	4.4	129.25	27.15	3.63	10.96	14.78	3.84	0.579	0.321	9.47	3.360	4.98
**M8**	4.3	144.16	23.42	4.03	11.40	13.47	3.50	0.741	0.324	8.34	2.651	4.44
**M9**	3.8	112.70	25.24	5.55	11.11	14.83	4.87	0.506	0.240	4.01	3.400	8.62
**M18**	4.3	145.30	26.89	1.692	10.13	13.11	2.617	0.2889	0.365	7.13	2.559	4.33

Table 26: Absolute Organ Weight (g) – Group G2 (720 mg/kg + vancomycin) – Individual animal data

Anim. No.	Body weight (kg)	Liver	Kidneys	Spleen	Brain	Heart	Thymus	Adrenals	Thyroid gland	Testes	Prostate	Epididym.
**M12**	4.4	106.29	24.29	1.751	9.99	10.71	2.263	0.544	0.2324	9.87	3.42	5.26
**M13**	4.4	186.51	25.92	1.682	10.14	14.01	3.370	0.578	0.3500	7.95	4.02	4.53
**M14**	4.3	162.24	23.86	2.111	11.29	12.70	2.661	0.783	0.3920	8.74	3.78	3.51
**M16**	4.6	146.04	20.65	1.635	10.89	10.12	5.09	0.613	0.402	5.80	2.773	3.50
**M17**	4.6	167.22	29.54	2.294	10.72	11.66	3.99	0.782	0.359	8.72	3.230	4.85

Table 27: Organ-to Body Weight Ratio (g/kg) – Group C (0 mg/kg) – Individual animal data

Anim. No.	Liver	Kidneys	Spleen	Brain	Heart	Thymus	Adrenals	Thyroid gland	Testes	Prostate	Epididym.
**M1**	30.57	5.08	0.379	2.74	3.77	0.650	0.080	0.108	1.78	0.508	0.94
**M2**	33.06	6.05	0.412	1.82	2.51	0.586	0.103	0.120	1.63	0.510	0.93
**M3**	31.98	6.05	0.543	2.39	3.60	0.707	0.088	0.072	1.93	0.597	0.87
**M4**	34.65	6.47	0.717	2.39	4.00	1.143	0.113	0.081	1.81	0.427	0.93
**M5**	39.80	5.95	0.328	2.24	3.89	0.640	0.114	0.090	2.33	0.578	1.14

Table 28: Organ-to Body Weight Ratio (g/kg) – Group G1 (720 mg/kg) – Individual animal data

Anim. No.	Liver	Kidneys	Spleen	Brain	Heart	Thymus	Adrenals	Thyroid gland	Testes	Prostate	Epididym.
**M6**	29.38	6.17	0.825	2.49	3.36	0.873	0.132	0.073	2.15	0.764	1.13
**M8**	33.53	5.45	0.937	2.65	3.13	0.814	0.172	0.075	1.94	0.617	1.03
**M9**	29.66	6.64	1.461	2.92	3.90	1.282	0.133	0.063	1.06	0.895	2.27
**M18**	33.79	6.25	0.393	2.36	3.05	0.609	0.067	0.085	1.66	0.595	1.01

Table 29: Organ-to Body Weight Ratio (g/kg) – Group G2 (720 mg/kg + vancomycin) – Individual animal data

Anim. No.	Liver	Kidneys	Spleen	Brain	Heart	Thymus	Adrenals	Thyroid gland	Testes	Prostate	Epididym.
**M12**	24.16	5.52	0.398	2.27	2.43	0.514	0.124	0.053	2.24	0.777	1.20
**M13**	42.39	5.89	0.382	2.30	3.18	0.766	0.131	0.080	1.81	0.914	1.03
**M14**	37.73	5.55	0.491	2.63	2.95	0.619	0.182	0.091	2.03	0.879	0.82
**M16**	31.75	4.49	0.355	2.37	2.20	1.107	0.133	0.087	1.26	0.603	0.76
**M17**	36.35	6.42	0.499	2.33	2.53	0.867	0.170	0.078	1.90	0.702	1.05

Table 30: Absolute Organ Weight (g) – Statistics, mean, median and SD values

Group	Exam. No.:	Body weight (kg)	Liver	Kidneys	Spleen	Brain	Heart	Thymus	Adrenals	Thyroid gland	Testes	Prostate	Epididym.
C							x	x					x
	N	5	5	5	5	5	5	5	5	5	5	5	5
	Mean	4.38	148.618	25.954	2.0798	10.078	15.402	3.2380	0.4362	0.4156	8.244	2.2946	4.198
	Median	4.20	145.510	26.620	2.0620	10.040	15.860	2.9280	0.4680	0.3700	8.130	2.3680	3.930
	SD	0.36	15.583	3.334	0.6598	1.002	1.632	0.8930	0.0726	0.1160	0.837	0.3386	0.433
G1													x
	N	4	4	4	4	4	4	4	4	4	4	4	4
	Mean	4.20	132.853	25.675	3.7255	10.900	14.048	3.7068	0.5287	0.3125	7.238	2.9925	5.593
	Median	4.30	136.705	26.065	3.8300	11.035	14.125	3.6700	0.5425	0.3225	7.735	3.0055	4.710
	SD	0.27	15.296	1.725	1.5881	0.545	0.887	0.9312	0.1876	0.0523	2.354	0.4493	2.038
G2							***		***			***	
	N	5	5	5	5	5	**5**	5	**5**	5	5	**5**	5
	Mean	4.46	153.660	24.852	1.8946	10.606	**11.840**	3.4748	**0.6600**	0.3471	8.216	**3.4446**	4.330
	Median	4.40	162.240	24.290	1.7510	10.720	**11.660**	3.3700	**0.6130**	0.3590	8.720	**3.4200**	4.530
	SD	0.13	30.160	3.243	0.2914	0.538	**1.559**	1.1198	**0.1145**	0.0677	1.514	**0.4851**	0.796

Table 31: Organ-To Body Weight Ratio (g/kg) – Statistics, mean, median and SD values

Group	Exam. No.:	Liver	Kidneys	Spleen	Brain	Heart	Thymus	Adrenals	Thyroid gland	Testes	Prostate	Epididym.
C						x	x					x
	N	5	5	5	5	5	5	5	5	5	5	5
	Mean	34.011	5.920	0.4759	2.318	3.556	0.7450	0.0996	0.0941	1.894	0.5240	0.961
	Median	33.062	6.050	0.4124	2.390	3.771	0.6500	0.1028	0.0902	1.807	0.5100	0.930
	SD	3.562	0.513	0.1564	0.334	0.600	0.2266	0.0152	0.0198	0.266	0.0672	0.106
G1												x
	N	4	4	4	4	4	4	4	4	4	4	4
	Mean	31.587	6.128	0.9041	2.605	3.361	0.8942	0.1261	0.0741	1.701	0.7175	1.360
	Median	31.592	6.212	0.8811	2.571	3.246	0.8433	0.1324	0.0742	1.799	0.6901	1.082
	SD	2.396	0.499	0.4388	0.244	0.384	0.2820	0.0435	0.0089	0.476	0.1399	0.608
G2						*****					*******	
	N	5	5	5	5	**5**	5	5	5	**5**	**5**	5
	Mean	34.475	5.574	0.4251	2.380	**2.661**	0.7746	0.1481	0.0778	1.848	**0.7750**	0.971
	Median	36.352	5.549	0.3980	2.330	**2.535**	0.7659	0.1333	0.0795	1.896	**0.7773**	1.030
	SD	6.904	0.707	0.0655	0.142	**0.400**	0.2296	0.0261	0.0150	0.367	**0.1275**	0.179

Notes for Tables 30 and 31:
Statistical evaluation by ANOVA p with Dunnett's MC test (parametric test for means) and Kruskal-Wallis test p with Dunn's MC test (non-parametric test for medians)
Statistically significant difference at the 95.0 % confidence level
* Statistically significant difference only between means test groups G1 or G2 versus control group C
*** Statistically significant difference between means and medians test groups G1 or G2 versus control group C
x Normality test not passed (for N > 4) - non-parametric test should be used for evaluation

TABLE SET VIII

Pathology Examination

Notes for tables 32- 71:

**M**	Male animal
-	Organ examined, no pathology findings
/	Organ not examined
**NA**	Not applicable
**GRADE 1**	Minimal/very few/very small
**GRADE 2**	Slight/few/small
**GRADE 3**	Moderate/moderate number/moderate size
**GRADE 4**	Marked/many/large
)	Finding unilateral in paired organs
**P**	Finding present, severity not scored
*	deceased animal
°	spare animal
+	euthanized moribund

Table 32: Severity grade of gross pathology findings in male rabbits of the C group

**Dose group:**	**C**				
**Sex:**	**M**				
**Animal number:**	**M1**	**M2**	**M3**	**M4**	**M5**
**Femur (implant. site)**					
**Right**					
- focal depression	-	-	1	-	-
- solid protuberance	1	-	-	-	-
- red focus	-	1	-	-	1
**Left**					
- solid protuberance	-	2	-	-	-
- red focus	1	-	-	-	-
**Lungs**					
- focal hemorrhage	2	-	-	-	-
**Trachea**					
- hyperemia	-	2	2	-	2

Table 33: Severity grade of gross pathology findings in male rabbits of the G1 group

**Dose group:**	**G1**					
**Sex:**	**M**					
**Animal number:**	**M6**	**M7***	**M8**	**M9**	**M10+**	**M18°**
**Kidneys**						
- granular surface	-	/	2	1	-	-
- red foci	-	/	2	-	-	-
**Femur (implant. site)**						
**Right**						
- fracture	-	/	-	-	P	-
- solid protuberance	3	/	3	-	-	-
**Left**						
- solid protuberance	2	/	-	-	-	-
- red focus	-	/	-	1	1	-
**Lungs**						
- focal hemorrhage	-	/	-	-	3	-
- red-brown foci	-	/	-	-	2	-
- whitish foci	-	/	-	-	2	-
- venostasis	3	/	-	-	-	2
**Muscle skeletal**						
**Hind leg left**						
- whitish granular substance	1	/	1	1	2	2
**Hind leg right**						
- whitish granular substance	1	/	1	1	-	-
**Muzzle**						
- yellowish discharge	-	/	-	-	P	-
**Trachea**						
- hemorrhage in mucosa	-	/	-	-	3	-

Table 34: Severity grade of gross pathology findings in male rabbits of the G2 group

**Dose group:**	**G2**						
**Sex:**	**M**						
**Animal number:**	**M11***	**M12**	**M13**	**M14**	**M15***	**M16°**	**M17°**
**Femur (implant. site)**							
**Right**							
- solid protuberance	/	-	2	-	/	2	-
- red focus	/	1	-	-	/	-	-
**Left**							
- fracture	/	-	P	-	/	-	-
**Lungs**							
- venostasis	/	-	3	1	/	3	3
**Muscle skeletal**							
**Hind leg left**							
- whitish granular substance	/	2	1	1	/	2	1
**Hind leg right**							
- whitish granular substance	/	2	1	1	/	1	1
**Trachea**							
- hyperemia	/	2	2	-	/	-	2

Table 35: Severity grade of microscopic findings in male rabbits of the C group

**Dose group:**	**C**				
**Sex:**	**M**				
**Animal number:**	**M1**	**M2**	**M3**	**M4**	**M5**
**Brain**					
- chronic ependymitis	-	-	-	-	2
- chronic meningitis	-	-	2	-	-
- granulomatous encephalitis	-	-	2	2	-
**Kidneys**					
- chronic pyelitis	-	2)	2)	-	1)
- cortical scar	-	-	3	-	-
- focal chron.inflamm.	-	1)	2	-	2
**Lacrimal gland**					
- focal chron.inflamm.	-	2	1	-	-
**Larynx**					
- edema	2	2	-	-	-
**Liver**					
- chronic productive pericholangoitis	-	1	2	2	1
- clear cell change	-	3	-	-	2
- venostasis	3	-	2	-	2
**Lungs**					
- chronic bronchitis	2	2	1	2	2
- venostasis	-	-	-	-	2
**Lymph nodes**					
**Cervical**					
- hyperplasia	2	-	-	-	-
**Prostate gland**					
- focal chron.inflamm.	-	-	-	-	1
- focal squamous metaplasia	1	-	1	1	-
**Spleen**					
- venostasis	-	-	-	-	2
**Thymus**					
- lipomatous atrophy	-	2	2	2	3
**Trachea**					
- chronic inflammation	2	2	-	2	-
- mucosal edema	2	3	2	2	1
- venostasis	-	-	2	2	2
**Urinary bladder**					
- foc.chron.inflamm.	-	-	-	1	-

Table 36: Severity grade of microscopic findings in male rabbits of the G1 group

**Dose group:**	**G1**					
**Sex:**	**M**					
**Animal number:**	**M6**	**M7***	**M8**	**M9**	**M10+**	**M18°**
**Brain**						
- granulomatous encephalitis	-	/	2	2	-	-
**Kidneys**						
- cortical scar	-	/	3	2	-	-
- focal chron.inflamm.	1	/	2	2	-	-
- venostasis	-	/	-	-	2	-
**Lacrimal gland**						
- focal chron.inflamm.	-	/	-	-	2	-
**Liver**						
- chronic productive pericholangoitis	-	/	2	1	2	1
- focal necrosis	-	/	-	1	-	-
- venostasis	3	/	2	3	3	3
**Lungs**						
- chronic bronchitis	-	/	2	2	-	-
- edema	3	/	-	-	3	3
- focal hemorrhage	-	/	-	2	3	-
- granuloma	-	/	-	1	-	-
- purulent bronchopneumonia	-	/	-	-	3	-
- purulent pleuritis	-	/	-	-	2	-
- venostasis	3	/	-	3	3	3
**Muscle skeletal**						
**Hind leg left**						
- foreign body granuloma	-	/	1	1	-	1
- scar	-	/	-	-	-	2
**Hind leg right**						
- foreign body granuloma	2	/	1	1	-	-
- scar	2	/	-	-	-	-
**Prostate gland**						
- focal squamous metaplasia	-	/	1	-	-	-
**Spinal cord**						
- granulomatous inflammation	-	/	1	-	-	-

To be continued

Table 36: (continued) Severity grade of microscopic findings in male rabbits of the G1 group

**Spleen**						
- venostasis	2	/	-	2	2	3
**Thymus**						
- lipomatous atrophy	-	/	-	-	3	3
**Thyroid gland**						
- nodular hyperplasia	-	/	-	2	-	-
**Trachea**						
- chronic inflammation	2	/	-	-	1	
- mucosal edema	2	/	-	1	-	2
- venostasis	3	/	-	-	3	2

Table 37: Severity grade of microscopic findings in male rabbits of the G2 group

**Dose group:**	**G2**						
**Sex:**	**M**						
**Animal number:**	**M11**	**M12**	**M13**	**M14**	**M15**	**M16**	**M17**
**Brain**							
- granulomatous encephalitis	/	-	-	-	/	-	2
**Kidneys**							
- cortical scar	/	-	-		/	-	1
- focal chron.inflamm.	/	2)	-	-	/	-	-
- pelvic edema	/	-	-	-	/	3	-
- venostasis	/	-	-	-	/	3	-
**Lacrimal gland**							
- focal chron.inflamm.	/	-	-	-	/	2	2
**Liver**							
- chron. productive pericholangoitis	/	1	1	-	/	-	1
- clear cell change	/	-	3	2	/	-	-
- focal hemorrhage	/	-	-	1	/	-	-
- venostasis	/	2	3	3	/	2	-
**Lungs**							
- chronic bronchitis	/	3	-	3	/	1	1
- edema	/	-	3	3	/	3	3
- venostasis	/	2	2	3	/	3	3
**Lymph nodes**							
**Iliac**							
- hemosiderin	/	2	-	-	/	-	-
**Muscle skeletal**							
**Hind leg left**							
- foreign body granuloma	/	2	1	1	/	2	1
- scar	/	2	2	2	/	2	1
**Hind leg right**							
- foreign body granuloma	/	1	1	1	/	2	1
- scar	/	2	2	2	/	2	-
**Prostate gland**							
- focal squamous metaplasia	/	1	1	-	/	-	-
**Spleen**							
- venostasis	/	2	-	-	/	-	-
**Thymus**							
- lipomatous atrophy	/	3	2	1	/	-	3

To be continued

Table 37: (continued) Severity grade of microscopic findings in male rabbits of the G2 group

**Trachea**							
- chronic inflammation	/	-	-	-	/	1	1
- mucosal edema	/	1	-	-	/	1	2
- venostasis	/	2	2	-	/	-	2

Table 38: Severity grade of microscopic findings in bone implantation site in male rabbit M1 of the C group

**Dose group:**	**C**					
**Animal No.:**	**M1**					
**Implantation site:**	**R1**	**R2**	**R3**	**L1**	**L2**	**L3**
**Femur**						
- bone defect healed	P	P	P	P	P	P
- periosteal fibrosis	1	1	1	2	2	2

Table 39: Severity grade of microscopic findings in bone implantation site in male rabbit M2 of the C group

**Dose group:**	**C**					
**Animal No.:**	**M2**					
**Implantation site:**	**R1**	**R2**	**R3**	**L1**	**L2**	**L3**
**Femur**						
- bone defect healed	P	P	P	P	P	P
- periosteal fibrosis	-	2	2	1	1	-

Table 40: Severity grade of microscopic findings in bone implantation site in male rabbit M3 of the C group

**Dose group:**	**C**					
**Animal No.:**	**M3**					
**Implantation site:**	**R1**	**R2**	**R3**	**L1**	**L2**	**L3**
**Femur**						
- bone defect healed	P	P	P	P	P	P
- periosteal fibrosis	2	3	1	2	1	1

Table 41: Severity grade of microscopic findings in bone implantation site in male rabbit M4 of the C group

**Dose group:**	**C**					
**Animal No.:**	**M4**					
**Implantation site:**	**R1**	**R2**	**R3**	**L1**	**L2**	**L3**
**Femur**						
- bone defect healed	P	P	P	P	P	P
- periosteal fibrosis	1	1	1	-	1	-

Table 42: Severity grade of microscopic findings in bone implantation site in male rabbit M5 of the C group.

**Dose group:**	**C**					
**Animal No.:**	**M5**					
**Implantation site:**	**R1**	**R2**	**R3**	**L1**	**L2**	**L3**
**Femur**						
- bone defect healed	P	P	P	P	P	P
- periosteal fibrosis	-	1	1	2	-	-

Table 43: Severity grade of microscopic findings in bone implantation site in male rabbit M6 of the G1 group

**Dose group:**	**G1**					
**Animal No.:**	**M6**					
**Implantation site:**	**R1**	**R2**	**R3**	**L1**	**L2**	**L3**
**Femur**						
- bone defect healed	P	P	P	P	P	P
- new bone formation	3	2	2	3	2	3
- foreign body granuloma in bone marrow	3	2	2	3	2	3
- foreign body granuloma in thigh muscle	1	1	2	1	1	2
- periosteal fibrosis	1	2	1	2	2	2

Table 44: Severity grade of microscopic findings in bone implantation site in male rabbit M8 of the G1 group

**Dose group:**	**G1**					
**Animal No.:**	**M8**					
**Implantation site:**	**R1**	**R2**	**R3**	**L1**	**L2**	**L3**
**Femur**						
- bone defect healed	P	P	P	P	P	P
- new bone formation	3	3	3	3	3	3
- foreign body granuloma in bone marrow	3	4	3	3	3	3
- foreign body granuloma in thigh muscle	1	1	2	1	1	2
- periosteal fibrosis	1	2	2	2	2	2

Table 45: Severity grade of microscopic findings in bone implantation site in male rabbit M9 of the G1 group

Dose group:	G1					
**Animal No.:**	**M9**					
**Implantation site:**	**R1**	**R2**	**R3**	**L1**	**L2**	**L3**
**Femur**						
- bone defect healed	P	P	P	P	P	P
- new bone formation	3	3	3	3	3	3
- foreign body granuloma in bone marrow	3	3	3	3	3	3
- foreign body granuloma in thigh muscle	-	1	2	2	2	2
- periosteal fibrosis	1	1	2	1	2	2

Table 46: Severity grade of microscopic findings in bone implantation site in male rabbit M10 of the G1 group

Dose group:	G1					
**Animal No.:**	**M10**					
**Implantation site:**	**R1**	**R2**	**R3**	**L1**	**L2**	**L3**
**Femur**						
- bone defect healed	NA	NA	P	P	P	P
- fracture	P	P	-	-	-	-
- new bone formation	3	3	-	2	3	-
- foreign body granuloma in bone marrow	3	3	-	3	3	-
- foreign body granuloma in thigh muscle	2	2	1	1	-	1
-organizing hematoma	3	3	-	-	-	-
- ossifying fibrous callus	3	3	-	-	-	-
- periosteal fibrosis	3	3	1	1	1	1

Table 47: Severity grade of microscopic findings in bone implantation site in male rabbit M18 of the G1 group

**Dose group:**	**G1**					
**Animal No.:**	**M18**					
**Implantation site:**	**R1**	**R2**	**R3**	**L1**	**L2**	**L3**
**Femur**						
- bone defect healed	P	P	P	P	P	P
- new bone formation	3	3	2	3	2	2
- foreign body granuloma in bone marrow	3	3	2	3	2	2
- foreign body granuloma in thigh muscle	-	1	2	2	1	2
- periosteal fibrosis	-	1	2	2	1	1

Table 48: Severity grade of microscopic findings in bone implantation site in male rabbit M12 of the G2 group

**Dose group:**	**G2**					
**Animal No.:**	**M12**					
**Implantation site:**	**R1**	**R2**	**R3**	**L1**	**L2**	**L3**
**Femur**						
- bone defect healed	P	P	P	P	P	P
- new bone formation	2	2	2	3	-	1
- foreign body granuloma in bone marrow	2	3	3	3	2	1
- foreign body granuloma in thigh muscle	1	1	2	2	2	2
- periosteal fibrosis	2	2	2	2	2	2

Table 49: Severity grade of microscopic findings in bone implantation site in male rabbit M13 of the G2 group

**Dose group:**	**G2**					
**Animal No.:**	**M13**					
**Implantation site:**	**R1**	**R2**	**R3**	**L1**	**L2**	**L3**
**Femur**						
- bone defect healed	P	P	P	P	P	P
- new bone formation	2	3	3	-	2	-
- foreign body granuloma in bone marrow	2	3	3	1	2	-
- foreign body granuloma in thigh muscle	-	1	1	1	1	-
- fracture	-	-	-	-	-	P
- osseous callus	-	-	-	-	-	3
- periosteal fibrosis	1	2	1	1	2	-

Table 50: Severity grade of microscopic findings in bone implantation site in male rabbit M14 of the G2 group

**Dose group:**	**G2**					
**Animal No.:**	**M14**					
**Implantation site:**	**R1**	**R2**	**R3**	**L1**	**L2**	**L3**
**Femur**						
- bone defect healed	P	P	P	P	P	P
- new bone formation	1	2	2	3	2	3
- foreign body granuloma in bone marrow	2	3	2	3	3	3
- foreign body granuloma in thigh muscle	-	1	2	1	1	2
- periosteal fibrosis	-	2	2	2	2	2

Table 51: Severity grade of microscopic findings in bone implantation site in male rabbit M16 of the G2 group

**Dose group:**	**G2**					
**Animal No.:**	**M16**					
**Implantation site:**	**R1**	**R2**	**R3**	**L1**	**L2**	**L3**
**Femur**						
- bone defect healed	P	P	P	P	P	P
- new bone formation	3	2	2	3	2	-
- foreign body granuloma in bone marrow	3	2	2	3	3	1
- foreign body granuloma in thigh muscle	-	2	-	1	1	1
- periosteal fibrosis	1	2	1	2	2	1

Table 52: Severity grade of microscopic findings in bone implantation site in male rabbit M17 of the G2 group

**Dose group:**	**G2**					
**Animal No.:**	**M17**					
**Implantation site:**	**R1**	**R2**	**R3**	**L1**	**L2**	**L3**
**Femur**						
- bone defect healed	P	P	P	P	P	P
- new bone formation	3	1	1	-	2	-
- foreign body granuloma in bone marrow	3	2	1	-	2	-
- foreign body granuloma in thigh muscle	1	1	1	1	2	-
- periosteal fibrosis	1	1	1	1	2	2

Table 53: Semi-quantitative evaluation of implantation sites in male rabbit M1 of the C group

**Test Sample:**	**Control**					
**Implantation Interval:**	**90 days**					
**Group:**	**C**					
**Animal number:**	**M1**					
**Implantation site:**	**R1**	**R2**	**R3**	**L1**	**L2**	**L3**
Polymorphonuclear cells	0	0	0	0	0	0
Lymphocytes	0	0	0	0	0	0
Plasma cells	0	0	0	0	0	0
Macrophages	0	0	0	0	0	0
Giant cells	0	0	0	0	0	0
Necrosis	0	0	0	0	0	0
**SUB-TOTAL (x 2)**	**0**	**0**	**0**	**0**	**0**	**0**
Neovascularisation	0	0	0	0	0	0
Fibrosis	1	1	1	2	2	2
Fatty infiltrate	0	0	0	0	0	0
**SUB-TOTAL**	**1**	**1**	**1**	**2**	**2**	**2**
**TOTAL**	**1**	**1**	**1**	**2**	**2**	**2**
**AVERAGE**	**1.5**					

Table 54: Semi-quantitative evaluation of implantation sites in male rabbit M2 of the C group

**Test Sample:**	**Control**					
**Implantation Interval:**	**90 days**					
**Group:**	**C**					
**Animal number:**	**M2**					
**Implantation site:**	**R1**	**R2**	**R3**	**L1**	**L2**	**L3**
Polymorphonuclear cells	0	0	0	0	0	0
Lymphocytes	0	0	0	0	0	0
Plasma cells	0	0	0	0	0	0
Macrophages	0	0	0	0	0	0
Giant cells	0	1	0	0	0	0
Necrosis	0	0	0	0	0	0
**SUB-TOTAL (x 2)**	**0**	**2**	**0**	**0**	**0**	**0**
Neovascularisation	0	0	0	0	0	0
Fibrosis	0	2	2	1	1	0
Fatty infiltrate	0	0	0	0	0	0
**SUB-TOTAL**	**0**	**2**	**2**	**1**	**1**	**0**
**TOTAL**	**0**	**4**	**2**	**1**	**1**	**0**
**AVERAGE**	**1.3**					

Table 55: Semi-quantitative evaluation of implantation sites in male rabbit M3 of the C group

**Test Sample:**	**Control**					
**Implantation Interval:**	**90 days**					
**Group:**	**C**					
**Animal number:**	**M3**					
**Implantation site:**	**R1**	**R2**	**R3**	**L1**	**L2**	**L3**
Polymorphonuclear cells	0	0	0	0	0	0
Lymphocytes	0	0	0	0	0	0
Plasma cells	0	0	0	0	0	0
Macrophages	0	0	0	0	0	0
Giant cells	1	1	0	0	0	0
Necrosis	0	0	0	0	0	0
**SUB-TOTAL (x 2)**	**2**	**2**	**0**	**0**	**0**	**0**
Neovascularisation	0	0	0	0	0	0
Fibrosis	2	3	1	2	1	1
Fatty infiltrate	0	0	0	0	0	0
**SUB-TOTAL**	**2**	**3**	**1**	**2**	**1**	**1**
**TOTAL**	**4**	**5**	**1**	**2**	**1**	**1**
**AVERAGE**	**2.3**					

Table 56: Semi-quantitative evaluation of implantation sites in male rabbit M4 of the C group

**Test Sample:**	**Control**					
**Implantation Interval:**	**90 days**					
**Group:**	**C**					
**Animal number:**	**M4**					
**Implantation site:**	**R1**	**R2**	**R3**	**L1**	**L2**	**L3**
Polymorphonuclear cells	0	0	0	0	0	0
Lymphocytes	0	0	0	0	0	0
Plasma cells	0	0	0	0	0	0
Macrophages	0	0	0	0	0	0
Giant cells	0	0	0	0	0	0
Necrosis	0	0	0	0	0	0
**SUB-TOTAL (x 2)**	**0**	**0**	**0**	**0**	**0**	**0**
Neovascularisation	0	0	0	0	0	0
Fibrosis	1	1	1	0	1	0
Fatty infiltrate	0	0	0	0	0	0
**SUB-TOTAL**	**1**	**1**	**1**	**0**	**1**	**0**
**TOTAL**	**1**	**1**	**1**	**0**	**1**	**0**
**AVERAGE**	**0.7**					

Table 57: Semi-quantitative evaluation of implantation sites in male rabbit M5 of the C group

**Test Sample:**	**Control**					
**Implantation Interval:**	**90 days**					
**Group:**	**C**					
**Animal number:**	**M5**					
**Implantation site:**	**R1**	**R2**	**R3**	**L1**	**L2**	**L3**
Polymorphonuclear cells	0	0	0	0	0	0
Lymphocytes	0	0	0	0	0	0
Plasma cells	0	0	0	0	0	0
Macrophages	0	0	0	0	0	0
Giant cells	0	0	0	0	0	0
Necrosis	0	0	0	0	0	0
**SUB-TOTAL (x 2)**	**0**	**0**	**0**	**0**	**0**	**0**
Neovascularisation	0	0	0	0	0	0
Fibrosis	0	1	1	2	0	0
Fatty infiltrate	0	0	0	0	0	0
**SUB-TOTAL**	**0**	**1**	**1**	**2**	**0**	**0**
**TOTAL**	**0**	**1**	**1**	**2**	**0**	**0**
**AVERAGE**	**0.7**					

Table 58: Semi-quantitative evaluation of implantation sites in male rabbit M6 of the G1 group

**Test Sample:**	**HECOLCAP**					
**Implantation Interval:**	**90 days**					
**Group:**	**G1**					
**Animal number:**	**M6**					
**Implantation site:**	**R1**	**R2**	**R3**	**L1**	**L2**	**L3**
Polymorphonuclear cells	0	0	0	0	0	0
Lymphocytes	1	0	1	0	0	0
Plasma cells	0	0	0	0	0	0
Macrophages	0	0	0	0	0	0
Giant cells	3	3	3	3	3	3
Necrosis	0	0	0	0	0	0
**SUB-TOTAL (x 2)**	**8**	**6**	**8**	**6**	**6**	**6**
Neovascularisation	0	0	0	0	0	0
Fibrosis	1	2	1	2	2	2
Fatty infiltrate	0	0	0	0	0	0
**SUB-TOTAL**	**1**	**2**	**1**	**2**	**2**	**2**
**TOTAL**	**9**	**8**	**9**	**8**	**8**	**8**
**AVERAGE**	**8.3**					

Table 59: Semi-quantitative evaluation of implantation sites in male rabbit M8 of the G1 group

**Test Sample:**	**HECOLCAP**					
**Implantation Interval:**	**90 days**					
**Group:**	**G1**					
**Animal number:**	**M8**					
**Implantation site:**	**R1**	**R2**	**R3**	**L1**	**L2**	**L3**
Polymorphonuclear cells	0	0	0	0	0	0
Lymphocytes	0	0	0	0	0	0
Plasma cells	0	0	0	0	0	0
Macrophages	0	0	0	0	0	0
Giant cells	3	4	3	3	3	3
Necrosis	0	0	0	0	0	0
**SUB-TOTAL (x 2)**	**6**	**8**	**6**	**6**	**6**	**6**
Neovascularisation	0	0	0	0	0	0
Fibrosis	1	2	2	2	2	2
Fatty infiltrate	0	0	0	0	0	0
**SUB-TOTAL**	**1**	**2**	**2**	**2**	**2**	**2**
**TOTAL**	**7**	**10**	**8**	**8**	**8**	**8**
**AVERAGE**	**8.2**					

Table 60: Semi-quantitative evaluation of implantation sites in male rabbit M9 of the G1 group

**Test Sample:**	**HECOLCAP**					
**Implantation Interval:**	**90 days**					
**Group:**	**G1**					
**Animal number:**	**M9**					
**Implantation site:**	**R1**	**R2**	**R3**	**L1**	**L2**	**L3**
Polymorphonuclear cells	0	0	0	0	0	0
Lymphocytes	0	0	0	0	0	0
Plasma cells	0	0	0	0	0	0
Macrophages	0	0	0	0	0	0
Giant cells	3	3	3	3	3	3
Necrosis	0	0	0	0	0	0
**SUB-TOTAL (x 2)**	**6**	**6**	**6**	**6**	**6**	**6**
Neovascularisation	0	0	0	0	0	0
Fibrosis	1	1	2	1	2	2
Fatty infiltrate	0	0	0	0	0	0
**SUB-TOTAL**	**1**	**1**	**2**	**1**	**2**	**2**
**TOTAL**	**7**	**7**	**8**	**7**	**8**	**8**
**AVERAGE**	**7.5**					

Table 61: Semi-quantitative evaluation of implantation sites in male rabbit M10 of the G1 group

Test Sample:	HECOLCAP					
Implantation Interval:	90 days					
Group:	G1					
Animal number:	M10					
Implantation site:	R1*	R2*	R3	L1	L2	L3
Polymorphonuclear cells	0	0	0	0	0	0
Lymphocytes	2	2	2	2	1	0
Plasma cells	3	3	3	0	0	0
Macrophages	3	2	0	0	0	0
Giant cells	3	3	0	3	3	0
Necrosis	0	0	0	0	0	0
**SUB-TOTAL (x 2)**	**22**	**20**	**10**	**10**	**8**	**0**
Neovascularisation	0	0	0	0	0	0
Fibrosis	3	3	1	1	1	1
Fatty infiltrate	0	0	0	0	0	0
**SUB-TOTAL**	**3**	**3**	**1**	**1**	**1**	**1**
**TOTAL**	**25**	**23**	**11**	**11**	**9**	**1**
**AVERAGE**	**13.3**					

* fracture, evaluation not included

Table 62: Semi-quantitative evaluation of implantation sites in male rabbit M18 of the G1 group

Test Sample:	HECOLCAP					
Implantation Interval:	90 days					
Group:	G1					
Animal number:	M18					
Implantation site:	R1	R2	R3	L1	L2	L3
Polymorphonuclear cells	0	0	0	0	0	0
Lymphocytes	1	1	2	1	1	1
Plasma cells	0	0	0	0	0	0
Macrophages	0	0	0	0	0	0
Giant cells	3	3	3	3	3	3
Necrosis	0	0	0	0	0	0
**SUB-TOTAL (x 2)**	**8**	**8**	**10**	**8**	**8**	**8**
Neovascularisation	0	0	0	0	0	0
Fibrosis	0	1	2	2	1	1
Fatty infiltrate	0	0	0	0	0	0
**SUB-TOTAL**	**0**	**1**	**2**	**2**	**1**	**1**
**TOTAL**	**8**	**9**	**12**	**10**	**9**	**9**
**AVERAGE**	**9.5**					

Table 63: Semi-quantitative evaluation of implantation sites in male rabbit M12 of the G2 group

**Test Sample:**	**HECOLCAP + vancomycin**					
**Implantation Interval:**	**90 days**					
**Group:**	**G2**					
**Animal number:**	**M12**					
**Implantation site:**	**R1**	**R2**	**R3**	**L1**	**L2**	**L3**
Polymorphonuclear cells	0	0	0	0	0	0
Lymphocytes	2	1	1	1	1	0
Plasma cells	0	0	0	0	0	0
Macrophages	0	0	0	0	0	0
Giant cells	3	3	3	3	2	1
Necrosis	0	0	0	0	0	0
**SUB-TOTAL (x 2)**	**10**	**8**	**8**	**8**	**6**	**2**
Neovascularisation	0	0	0	0	0	0
Fibrosis	2	2	2	2	2	2
Fatty infiltrate	0	0	0	0	0	0
**SUB-TOTAL**	**2**	**2**	**2**	**2**	**2**	**2**
**TOTAL**	**12**	**10**	**10**	**10**	**8**	**4**
**AVERAGE**	**9.0**					

Table 64: Semi-quantitative evaluation of implantation sites in male rabbit M13 of the G2 group

**Test Sample:**	**HECOLCAP + vancomycin**					
**Implantation Interval:**	**90 days**					
**Group:**	**G2**					
**Animal number:**	**M13**					
**Implantation site:**	**R1**	**R2**	**R3**	**L1**	**L2**	**L3**
Polymorphonuclear cells	0	0	0	0	0	0
Lymphocytes	1	1	1	1	0	0
Plasma cells	0	0	0	0	0	0
Macrophages	0	0	0	0	0	0
Giant cells	2	3	3	1	0	0
Necrosis	0	0	0	0	0	0
**SUB-TOTAL (x 2)**	**6**	**8**	**8**	**4**	**0**	**0**
Neovascularisation	0	0	0	0	0	0
Fibrosis	1	2	1	1	2	0
Fatty infiltrate	0	0	0	0	0	0
**SUB-TOTAL**	**1**	**2**	**1**	**1**	**2**	**0**
**TOTAL**	**7**	**10**	**9**	**5**	**2**	**0**
**AVERAGE**	**5.5**					

Table 65: Semi-quantitative evaluation of implantation sites in male rabbit M14 of the G2 group

**Test Sample:**	**HECOLCAP + vancomycin**					
**Implantation Interval:**	**90 days**					
**Group:**	**G2**					
**Animal number:**	**M14**					
**Implantation site:**	**R1**	**R2**	**R3**	**L1**	**L2**	**L3**
Polymorphonuclear cells	0	0	0	0	0	0
Lymphocytes	2	1	1	0	0	0
Plasma cells	0	0	0	0	0	0
Macrophages	0	0	0	0	0	0
Giant cells	2	3	2	3	3	3
Necrosis	0	0	0	0	0	0
**SUB-TOTAL (x 2)**	**8**	**8**	**6**	**6**	**6**	**6**
Neovascularisation	0	0	0	0	0	0
Fibrosis	0	2	2	2	2	2
Fatty infiltrate	0	0	0	0	0	0
**SUB-TOTAL**	**0**	**2**	**2**	**2**	**2**	**2**
**TOTAL**	**8**	**10**	**8**	**8**	**8**	**8**
**AVERAGE**	**8.3**					

Table 66: Semi-quantitative evaluation of implantation sites in male rabbit M16 of the G2 group

**Test Sample:**	**HECOLCAP + vancomycin**					
**Implantation Interval:**	**90 days**					
**Group:**	**G2**					
**Animal number:**	**M16**					
**Implantation site:**	**R1**	**R2**	**R3**	**L1**	**L2**	**L3**
Polymorphonuclear cells	0	0	0	0	0	0
Lymphocytes	0	1	2	3	0	0
Plasma cells	0	0	0	0	0	0
Macrophages	0	0	0	0	0	0
Giant cells	3	2	2	3	3	1
Necrosis	0	0	0	0	0	0
**SUB-TOTAL (x 2)**	**6**	**6**	**8**	**12**	**6**	**2**
Neovascularisation	0	0	0	0	0	0
Fibrosis	1	2	1	2	2	1
Fatty infiltrate	0	0	0	0	0	0
**SUB-TOTAL**	**1**	**2**	**1**	**2**	**2**	**1**
**TOTAL**	**7**	**8**	**9**	**14**	**8**	**3**
**AVERAGE**	**8.2**					

Table 67: Semi-quantitative evaluation of implantation sites in male rabbit M17 of the G2 group

**Test Sample:**	**HECOLCAP + vancomycin**					
**Implantation Interval:**	**90 days**					
**Group:**	**G2**					
**Animal number:**	**M17**					
**Implantation site:**	**R1**	**R2**	**R3**	**L1**	**L2**	**L3**
Polymorphonuclear cells	0	0	0	0	0	0
Lymphocytes	0	0	0	0	1	0
Plasma cells	0	0	0	0	0	0
Macrophages	0	0	0	0	0	0
Giant cells	3	2	1	0	2	0
Necrosis	0	0	0	0	0	0
**SUB-TOTAL (x 2)**	**6**	**4**	**2**	**0**	**6**	**0**
Neovascularisation	0	0	0	0	0	0
Fibrosis	1	1	1	1	2	2
Fatty infiltrate	0	0	0	0	0	0
**SUB-TOTAL**	**1**	**1**	**1**	**1**	**2**	**2**
**TOTAL**	**7**	**5**	**3**	**1**	**8**	**2**
**AVERAGE**	**4.3**					

Table 68: Gross pathology findings - summary animal data

**Dose group:**	**C**	**G1**	**G2**
**Sex:**	**M**	**M**	**M**
**Number of animals:**	**5**	**5/6**	**5/7**
**Organ findings:**			
**Kidneys**			
- granular surface	-	2	-
- red foci	-	1	-
**Femur (implant. site)**			
**Right**			
- focal depression	1	-	-
- fracture	-	1	-
- solid protuberance	1	2	2
- red focus	2	-	1
**Left**			
- fracture	-	-	1
- solid protuberance	1	1	-
- red focus	1	2	-
**Lungs**			
- focal hemorrhage	1	1	-
- red-brown foci	-	1	-
- whitish foci	-	1	-
- venostasis	-	2	4
**Muscle skeletal**			
**Hind leg left**			
- whitish granular substance	-	5	5
**Hind leg right**			
- whitish granular substance	-	3	5
**Muzzle**			
- yellowish discharge	-	1	-
**Trachea**			
- hemorrhage in mucosa	-	1	-
- hyperemia	3	-	3

Table 69: Microscopic findings – summary animal data

**Dose group:**	**C**	**G1**	**G2**
**Sex:**	**M**	**M**	**M**
**Number of animals:**	**5**	**5/6**	**5/7**
**Organ findings:**			
**Brain**			
- chronic ependymitis	1	-	-
- chronic meningitis	1	-	-
- granulomatous encephalitis	2	2	1
**Kidneys**			
- chronic pyelitis	3	-	-
- cortical scar	1	2	1
- focal chronic inflamm.	3	3	1
- pelvic edema	-	-	1
- venostasis	-	1	-
**Lacrimal gland**			
- focal chronic inflamm.	2	1	2
**Liver**			
- chronic productive pericholangoitis	4	4	3
- clear cell change	2	-	2
- focal hemorrhage	-	-	1
- focal necrosis	-	1	-
- venostasis	3	5	4
**Lungs**			
- chronic bronchitis	5	2	4
- edema	-	3	4
- focal hemorrhage	-	2	-
- granuloma	-	1	-
- purulent bronchopneumonia	-	1	-
- purulent pleuritis	-	1	-
- venostasis	1	4	5
**Lymph nodes**			
**Cervical**			
- hyperplasia	1	-	-
**Iliac**			
- hemosiderin	-	-	1
**Muscle skeletal**			
**Hind leg left**			
- foreign body granuloma	-	3	5
- scar	-	1	5

To be continued

Table 69: (continued) Microscopic findings – summary animal data

**Hind leg right**			
- foreign body granuloma	-	3	5
- scar	-	1	4
**Prostate gland**			
- focal chronic inflamm.	1	-	-
- focal squamous metaplasia	3	1	2
**Spinal cord**			
- granulomatous inflammation	-	1	-
**Spleen**			
- venostasis	1	4	1
**Thymus**			
- lipomatous atrophy	4	2	4
**Thyroid gland**			
- nodular hyperplasia	-	1	-
**Trachea**			
- chronic inflammation	3	2	2
- mucosal edema	5	3	3
- venostasis	3	3	3
**Urinary bladder**			
- focal chronic inflamm.	1	-	-

Table 70: Microscopic findings in implantation sites – summary animal data

**Dose group:**	**C**	**G1**	**G2**
**Sex:**	**M**	**M**	**M**
**Number of animals:**	**5**	**5/6**	**5/7**
**No. of implant. sites**	**30**	**30**	**30**
**Organ findings:**			
**Femur**			
- bone defect healed	30	28	30
- fracture	-	2	1
- new bone formation	-	28	24
- foreign body granuloma in bone marrow	-	28	27
- foreign body granuloma in thigh muscle	-	27	24
- organizing hematoma	-	2	-
- ossifying fibrous callus	-	2	-
- periosteal fibrosis	23	29	28

Table 71: Average values of Irritation Index

Dose group	C	G1	G2
Test item	CONTROL	HECOLCAP	HECOLCAP + VANCOMYCIN
Administration	Bone implantation	Bone implantation	Bone implantation
Average Irritation Index	1.30	8.38	7.06
Irritation index (TI – Control)	-	7.08	5.76

Statement of Quality Assurance Unit

Study: **HECOLCAP, 90-day Subchronic Toxicity Study after Intra-osseous Implantation in Rabbits**

Date of the Final Report: 03. 11. 2020

Inspector QAU: K. Lacinová, K. Ptáčková

Below listed study-based inspections were carried out.

Study-based inspections:

Study phase	Date of inspection	Date of reporting to SD and Management
Study plan	03. 03. 2020	03. 03. 2020
Initiation phase	05. 03. 2020	05. 03. 2020
Initiation phase II	12. 03. 2020	12. 03. 2020
In-process phase I	10 – 12. 03. 2020	12. 03. 2020
In-process phase II	31. 03. 2020	31. 03. 2020
In-process phase III	08. – 10. 06. 2020	16. 06. 2020
In-process phase IV	29. 06. 2020	30. 06. 2020
Reporting phase (Final report)	03. 11. 2020	03. 11. 2020

The Final Report was reviewed, it fully and accurately reflects the procedures and raw data generated during the conduct of the study.

QAU certifies that the study presented has been carried out in accordance with approved Study Plan, valid Standard Operating Procedures, and in compliance with OECD Principles of Good Laboratory Practice.

During the study, the inspectors of QAU also carried out facility- and process-based inspections.

Regular inspections performed during the duration of the study:

Inspection	Date of inspection	Date of reporting to SD and Management
Facility-based	03. – 06. 03. 2020	09. 03. 2020
Processes-based	06. – 09. 04. 2020	14. 04. 2020
Inspection of Department of Biochemistry	14. 05. 2020	18. 05. 2020
Facility-based	16. – 18. 06. 2020	22. 06. 2020
Facility-based	15. 08. 2020	17. 08. 2020
Facility-based	09. – 10. 09. 2020	10. 09. 2020
Processes-based	06. – 08. 10. 2020	09. 10. 2020

Date: 03. 11. 2020

Kateřina Ptáčková
Head of Quality Assurance Unit

Quality Assurance Unit MEDITOX s.r.o. Good Laboratory Practice ①

GLP-certificate of the Test Facility

SÚKL

STÁTNÍ ÚSTAV
PRO KONTROLU LÉČIV

Šrobárova 48
100 41 Praha 10

Telefon: +420 272 185 111
Fax: +420 271 732 377

E-mail: posta@sukl.cz
Web: www.sukl.cz

Certifikát spisová zn.: / Certificate Ref. No.: sukls288373/2018

CERTIFIKÁT SPRÁVNÉ LABORATORNÍ PRAXE
CERTIFICATE OF GOOD LABORATORY PRACTICE

Vydaný v souladu s §13 odst. 2 písm. a bod 4 zákona č. 378/2007 Sb., o léčivech a o změnách některých souvisejících zákonů (zákon o léčivech), ve znění pozdějších předpisů

Příslušný orgán České republiky potvrzuje následující:

Testovací zařízení:
MediTox s.r.o.

Sídlo:
Pod Zámkem 279, 281 25 Konárovice

Adresa testovacího zařízení:
Pod Zámkem 279, 281 25 Konárovice

bylo inspektováno v souladu se článkem 3 směrnice 2004/9/ES a § 101, odst. 3 zákona č. 378/2007 Sb., o léčivech a o změnách některých souvisejících zákonů (zákon o léčivech), ve znění pozdějších předpisů a při této inspekci bylo provedeno posouzení souladu se správnou laboratorní praxí podle směrnice 2004/9/ES.

Na základě znalostí získaných během poslední inspekce tohoto testovacího zařízení, která byla provedena dne 23.08.2018, je toto testovací zařízení považováno za subjekt splňující principy správné laboratorní praxe dané

- Zásadami správné laboratorní praxe podle OECD (C(97) 186 v konečném znění)
- směrnicí 2004/10/ES a
- vyhláškou č. 86/2008 Sb., o stanovení zásad SLP

Tento certifikát odráží stav testovacího zařízení v čase výše zmíněné inspekce a nemělo by se spoléhat na to, že bude odrážet stav shody po uplynutí více než tří let od data inspekce. Po této době by měl být konzultován vydávající orgán.
Pravost tohoto certifikátu může být ověřena u vydávajícího orgánu.

Issued following in accordance with Section 13, paragraph 2, letter a, point 4 of the Act No 378/2007 Coll., on Pharmaceuticals and on Amendments to Some Related Acts (Act on Pharmaceuticals), as amended

The competent authority of the Czech Republic confirms the following:

Test facility:
MediTox s.r.o.

Head office:
Pod Zámkem 279, 281 25 Konárovice

Site address:
Pod Zámkem 279, 281 25 Konárovice

has been inspected in accordance with Art. 3 of Directive 2004/9/EC and Section 101 paragraph 3 of the Act No 378/2007 Coll., on Pharmaceuticals and on Amendments to Some Related Acts (Act on Pharmaceuticals), as amended and during this inspection assessment of conformity with GLP according to Directive 2004/9/EC has been performed.

From the knowledge gained during inspection of this test facility, the latest of which was conducted on 23/08/2018, it is considered that it complies with
the principles of good laboratory practice given by

- OECD Principles of Good Laboratory Practice (C(97) 186 final)
- Directive 2004/10/EC and
- Decree No 86/2008 Coll. on GLP principles

This certificate reflects the status of the test facility at the time of the inspection noted above and should not be relied upon to reflect the compliance status if more than three years have elapsed since the date of inspection, after which time the issuing authority should be consulted.
The authenticity of this certificate may be verified with the issuing authority.

Certifikát SLP sp.zn.: sukls288373/2018
Datum: 22.11.2018
Strana 1 z 2
Jméno: František Chuchma, v z.
e-mail: posta@sukl.cz
Podpis:

GLP Certificate Ref.No.: sukls288373/2018
Date: 22.11.2018
Page 1 / 2
Name
Phone number: +420 272 185 832
Signature of the authorised person of the competent authority

Rozsah činnosti
Toxikologické studie

Scope of activities
Toxicological studies

Jakékoli omezení nebo vysvětlení vztahující se k rozsahu certifikátu: ---

Any restrictions or clarifying remarks related to the scope of this certificate: ---

Datum: 22.11.2018

Date: 22.11.2018

podpis oprávněné osoby příslušného orgánu České republiky

signature of the authorised person of the competent authority of the Czech Republic

František Chuchma, v z.
ředitel inspekčního odboru

František Chuchma in deputy
Director of the Inspection Section

Státní ústav pro kontrolu léčiv
Šrobárova 48
100 41 Praha 10
Česká republika
e-mail: posta@sukl.cz
telefon: +420 272 185 832
fax: +420 271 732 377

State Institute for Drug Control
Šrobárova 48
100 41 Prague 10
Czech Republic
e-mail: posta@sukl.cz
phone: +420 272 185 832
fax: +420 271 732 377

SUPPLEMENTS II

Photo-documentation

Photo-documentation: Control Group C

Figure 1: Animal no. 1, left femur
Figure 2: Animal no. 1, right femur
Figure 3: Animal no. 2, left femur
Figure 4: Animal no. 2, right femur
Figure 5: Animal no. 3, left femur
Figure 6: Animal no. 3, right femur
Figure 7: Animal no. 4, left femur
Figure 8: Animal no. 4, right femur
Figure 9: Animal no. 5, left femur
Figure 10: Animal no. 5, right femur

Photo-documentation: Group G1

Figure 11: Animal no. 6, left femur
Figure 12: Animal no. 6, right femur
Figure 13: Animal no. 8, left femur
Figure 14: Animal no. 8, right femur
Figure 15: Animal no. 9, left femur
Figure 16: Animal no. 9, right femur
Figure 17: Animal no. 10, left femur
Figure 18: Animal no. 10, right femur
Figure 19: Animal no. 18, left femur
Figure 20: Animal no. 18, right femur

Photo-documentation: Group G2

Figure 21: Animal no. 12, left femur
Figure 22: Animal no. 12, right femur
Figure 23: Animal no. 13, left femur
Figure 24: Animal no. 13, right femur
Figure 25: Animal no. 14, left femur
Figure 26: Animal no. 14, right femur
Figure 27: Animal no. 16, left femur
Figure 28: Animal no. 16, right femur
Figure 29: Animal no. 17, left femur
Figure 30: Animal no. 17, right femur

X-Ray Photos

X-Ray photos: Control Group C

Figure 31: Animal no. 1, left femur
Figure 32: Animal no. 1, right femur
Figure 33: Animal no. 2, left femur
Figure 34: Animal no. 2, right femur
Figure 35: Animal no. 3, left femur
Figure 36: Animal no. 3, right femur
Figure 37: Animal no. 4, left femur
Figure 38: Animal no. 4, right femur
Figure 39: Animal no. 5, left femur
Figure 40: Animal no. 5, right femur

X-Ray photos: Group G1

Figure 41: Animal no. 6, left femur
Figure 42: Animal no. 6, right femur
Figure 43: Animal no. 8, left femur
Figure 44: Animal no. 8, right femur
Figure 45: Animal no. 9, left femur
Figure 46: Animal no. 9, right femur
Figure 47: Animal no. 10, left femur
Figure 48: Animal no. 10, right femur
Figure 49: Animal no. 18, left femur
Figure 50: Animal no. 18, right femur

X-Ray photos: Group G2

Figure 51: Animal no. 12, left femur
Figure 52: Animal no. 12, right femur
Figure 53: Animal no. 13, left femur
Figure 54: Animal no. 13, right femur
Figure 55: Animal no. 14, left femur
Figure 56: Animal no. 14, right femur
Figure 57: Animal no. 16, left femur
Figure 58: Animal no. 16, right femur
Figure 59: Animal no. 17, left femur
Figure 60: Animal no. 17, right femur

Photo-documentation: Control Group C

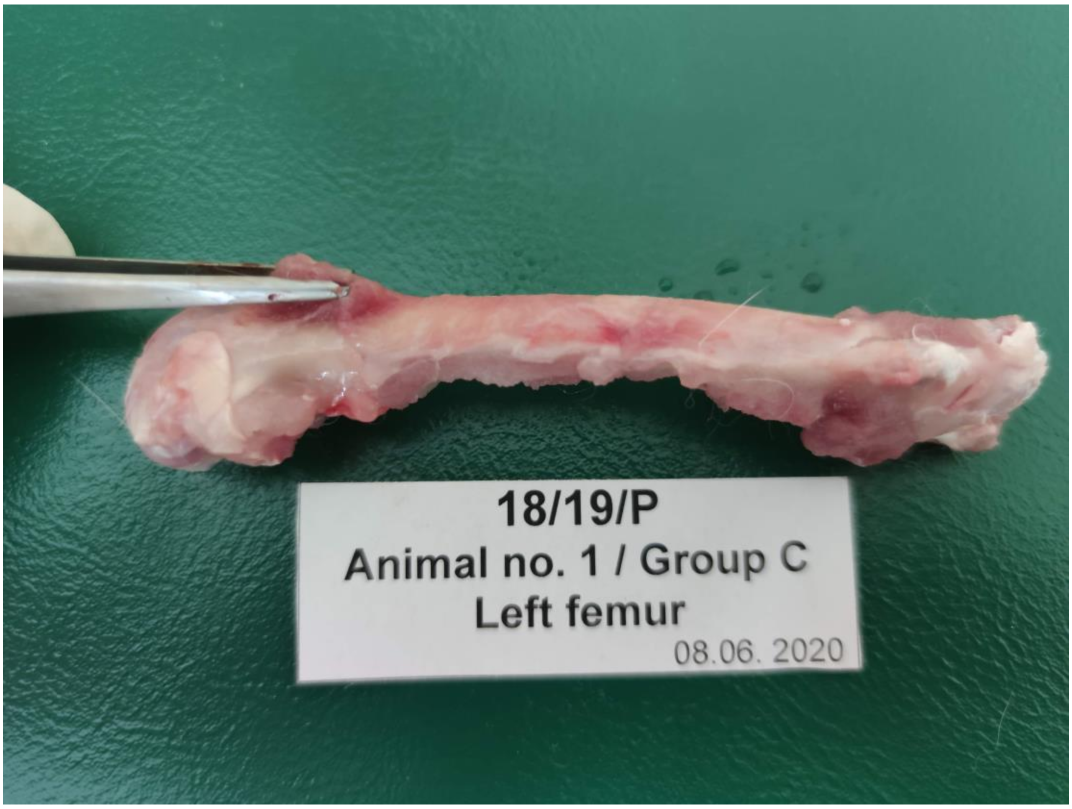

Figure 1: Animal no. 1, left femur

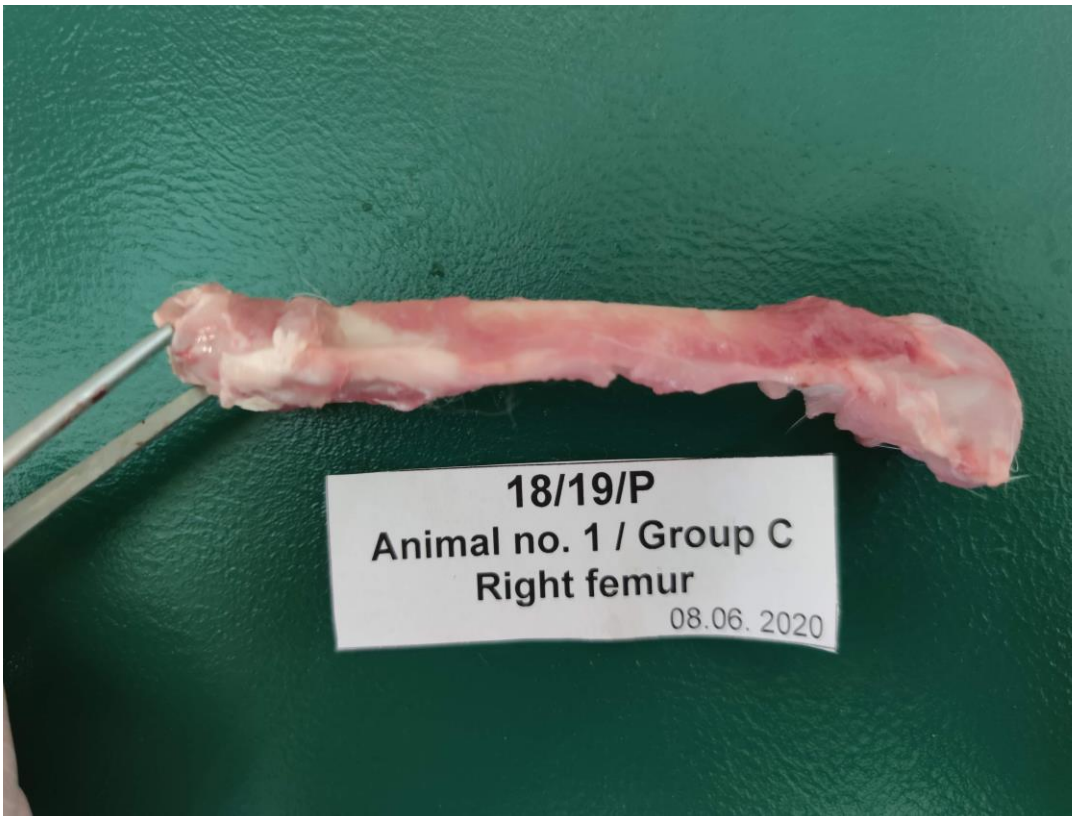

Figure 2: Animal no. 1, right femur

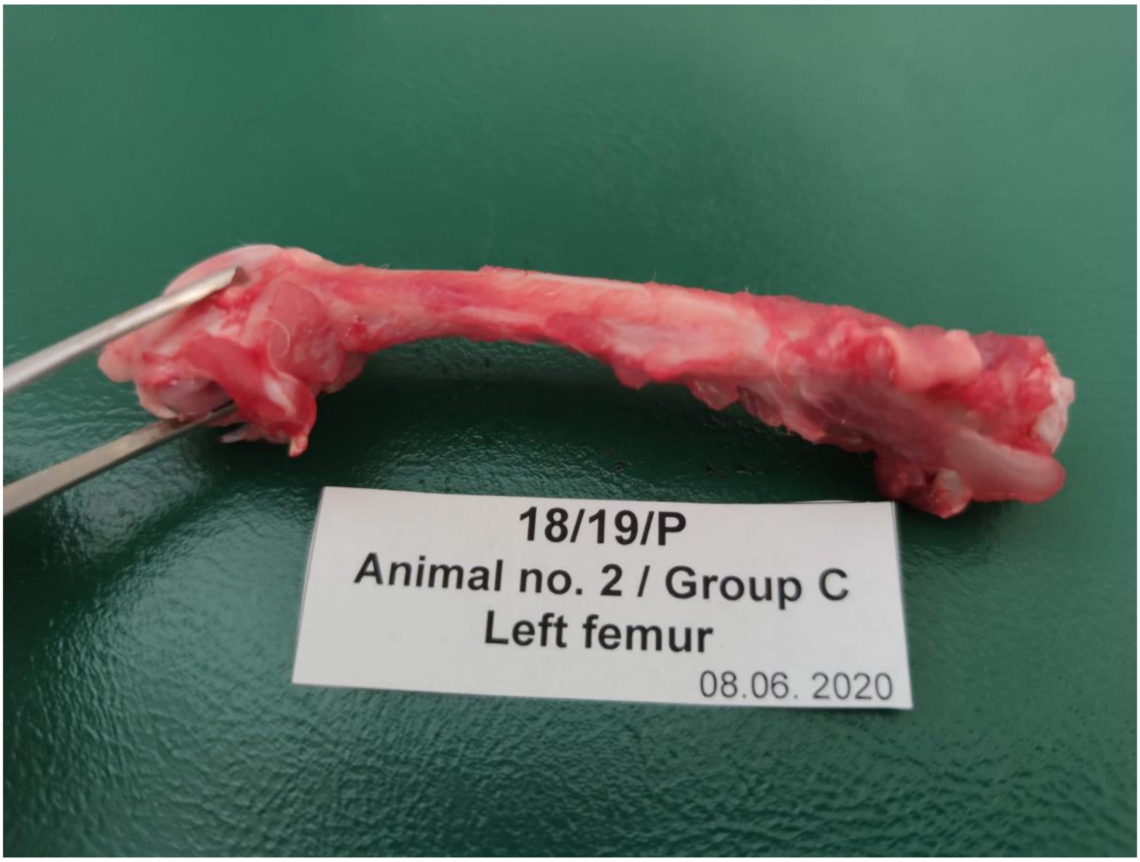

Figure 3: Animal no. 2, left femur

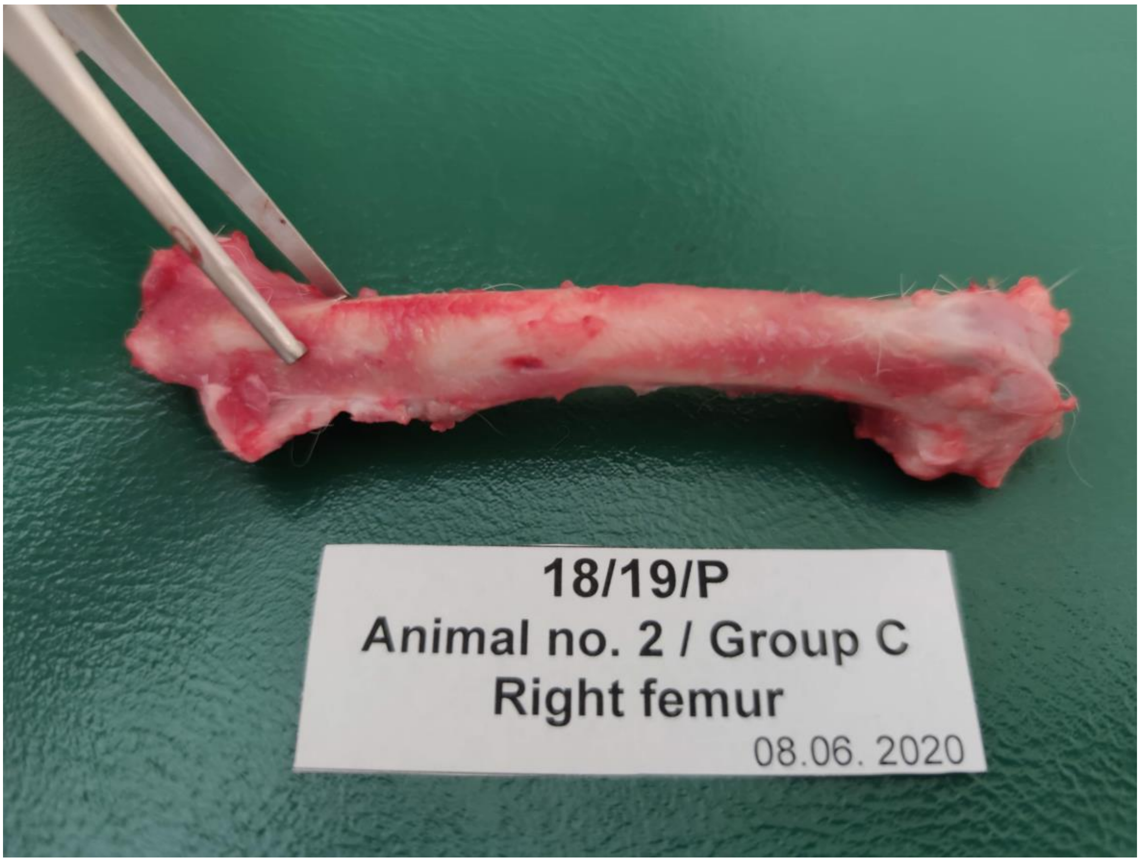

Figure 4: Animal no. 2, right femur

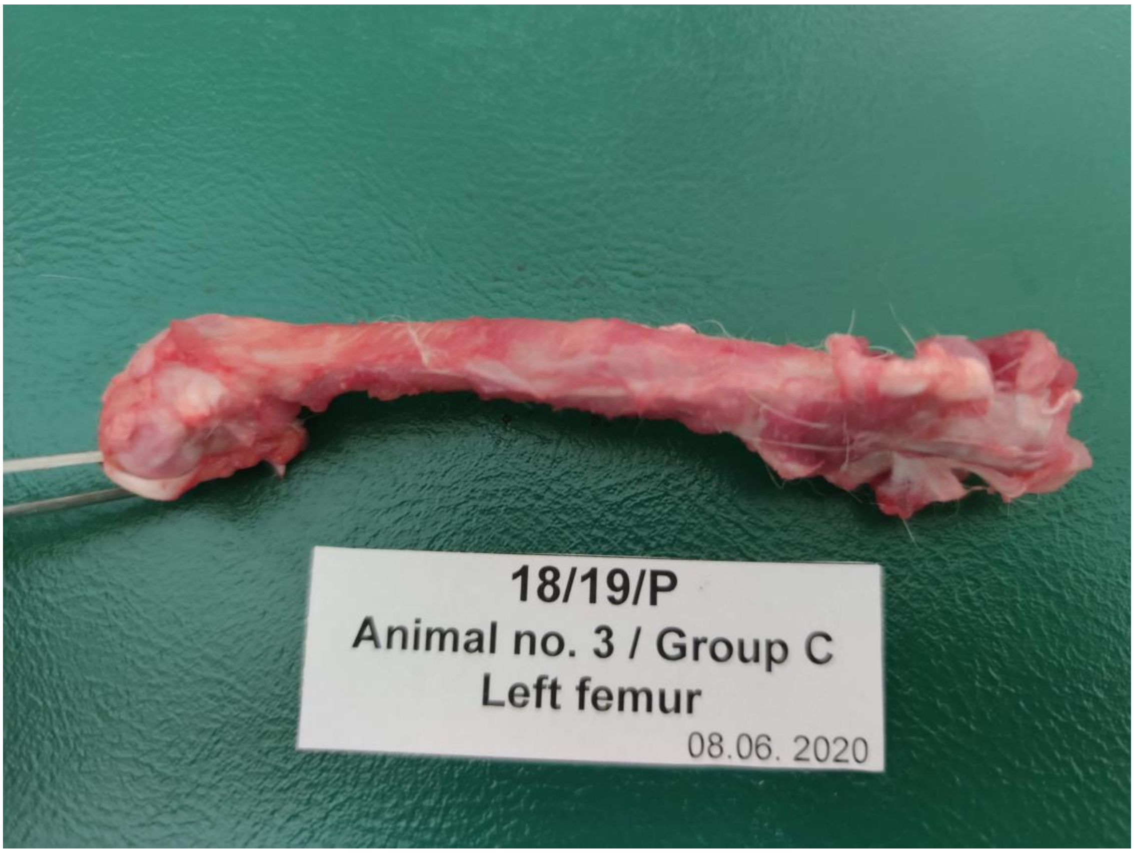

Figure 5: Animal no. 3, left femur

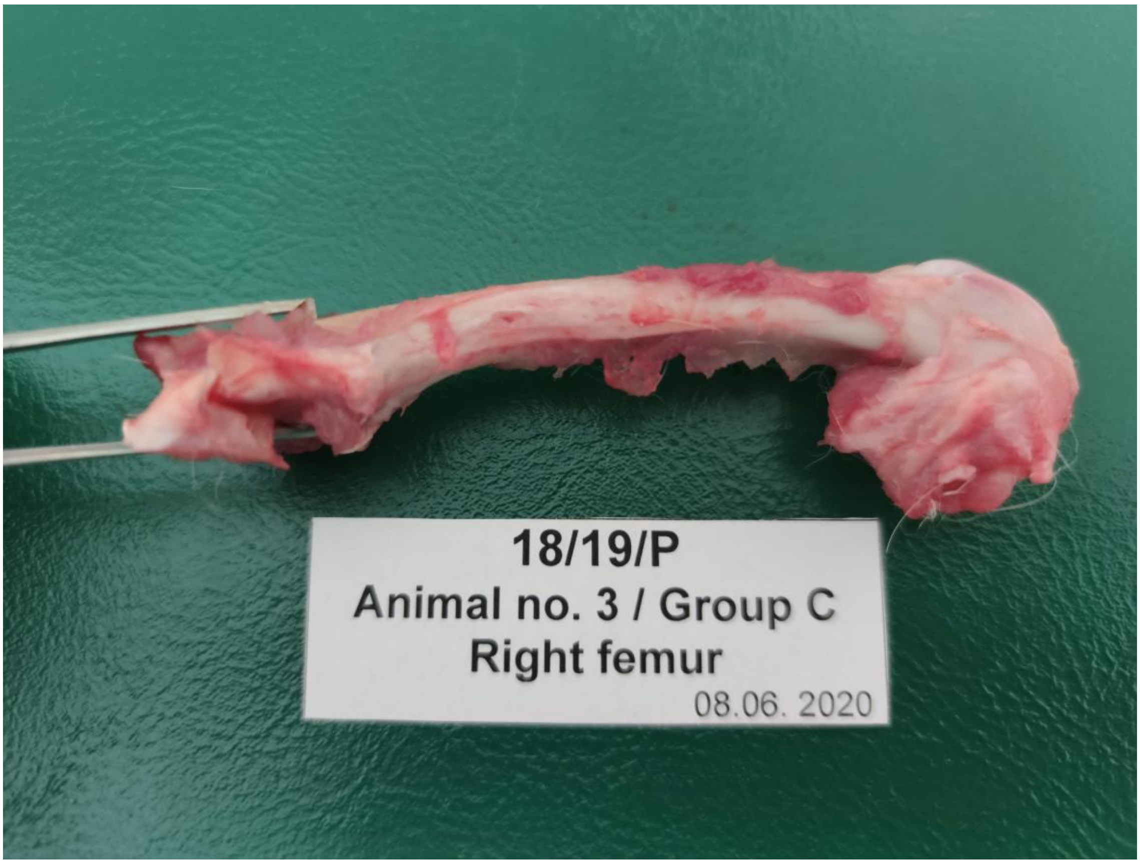

Figure 6: Animal no. 3, right femur

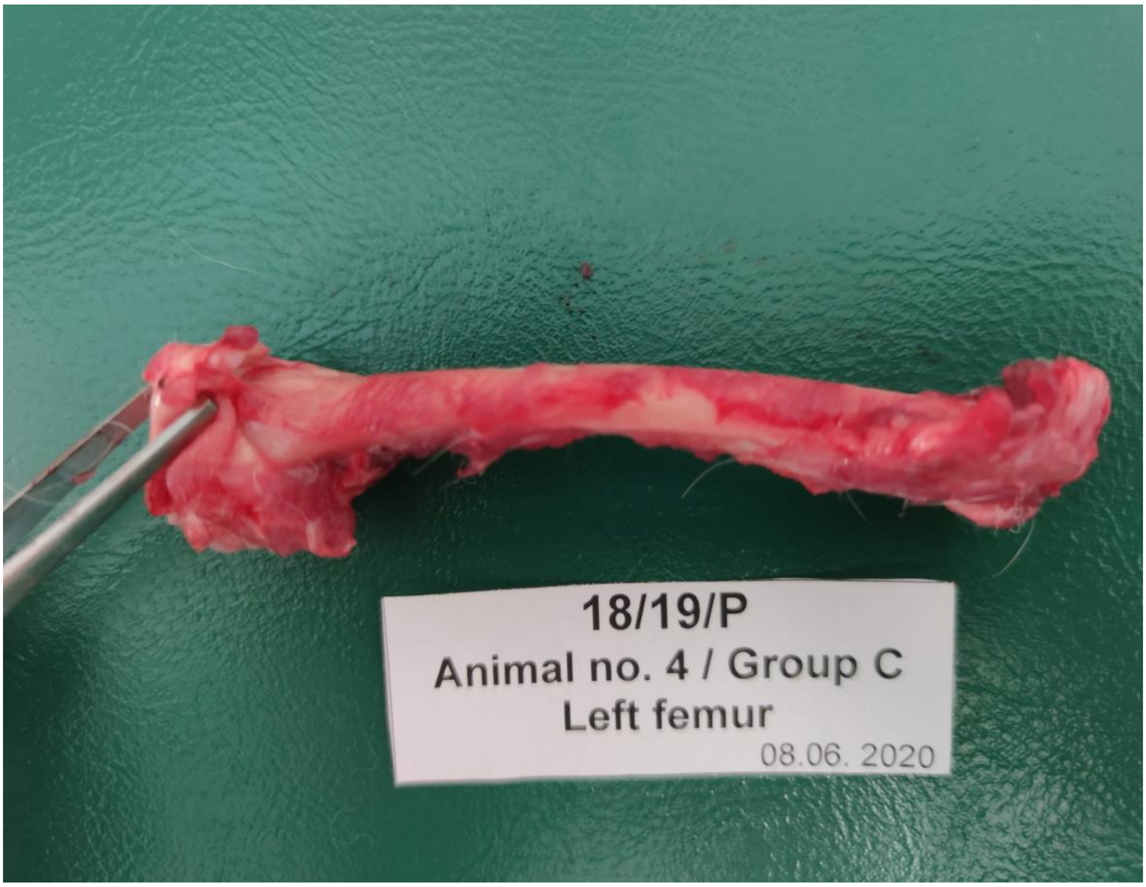

Figure 7: Animal no. 4, left femur

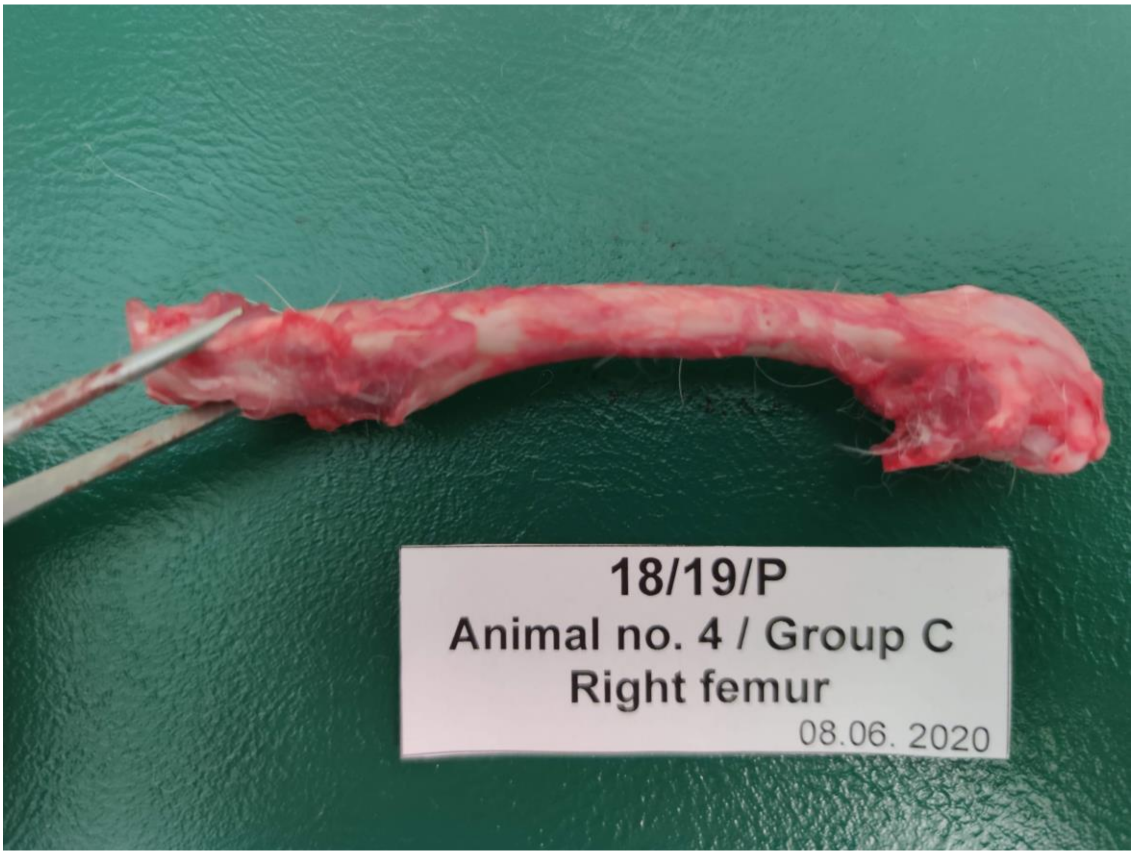

Figure 8: Animal no. 4, right femur

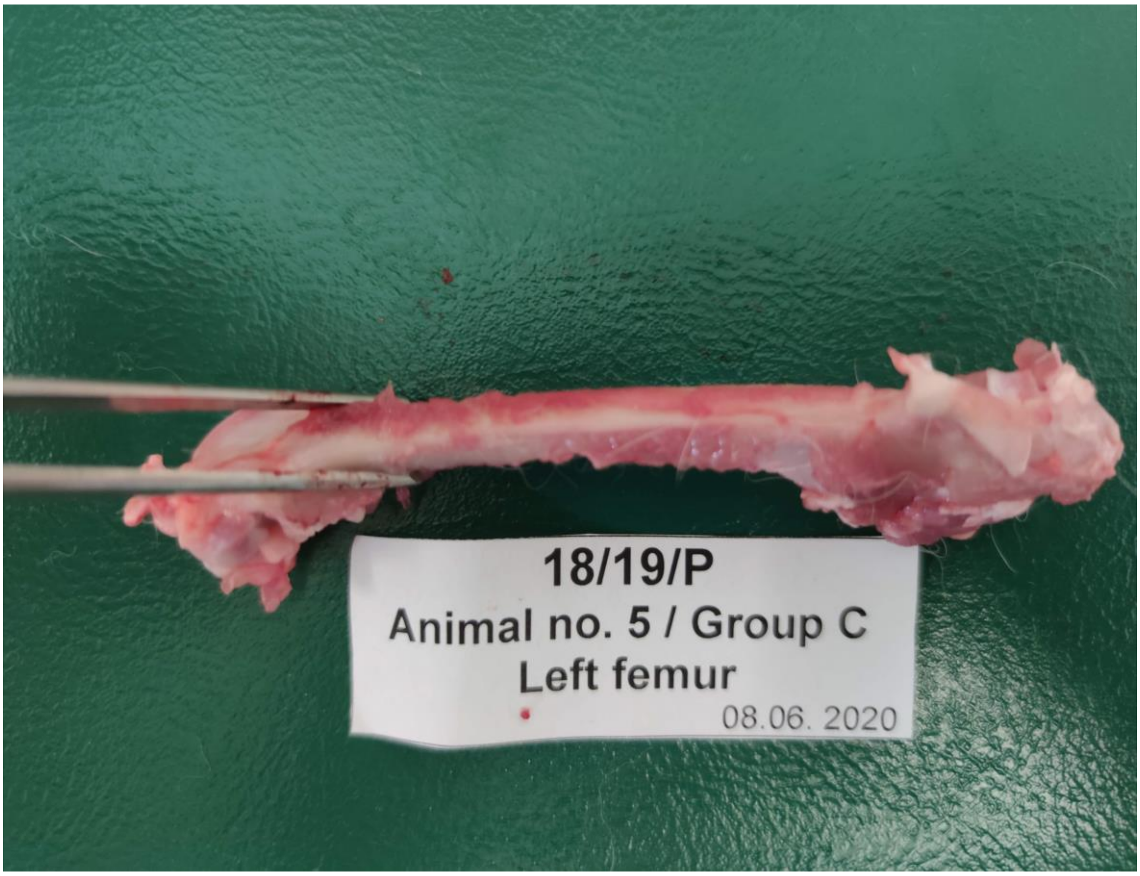

Figure 9: Animal no. 5, left femur

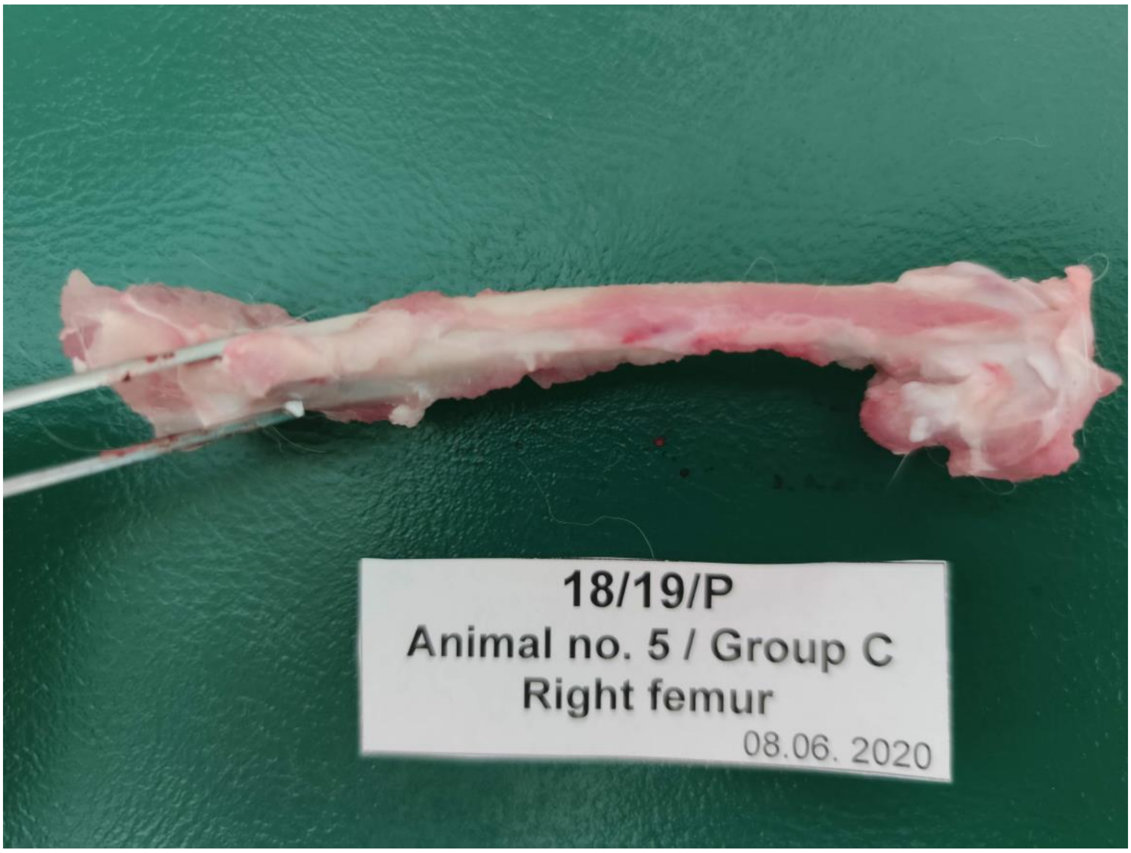

Figure 10: Animal no. 5, right femur

Photo-documentation: Group G1

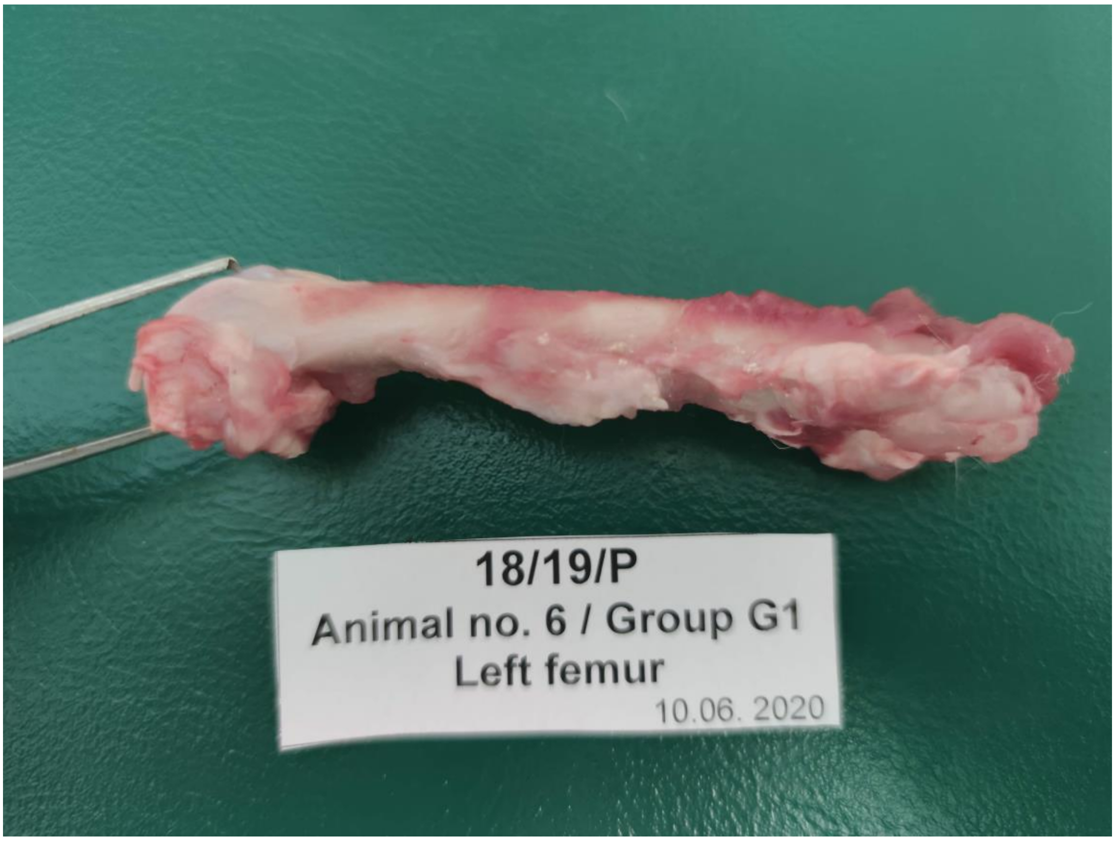

Figure 11: Animal no. 6, left femur

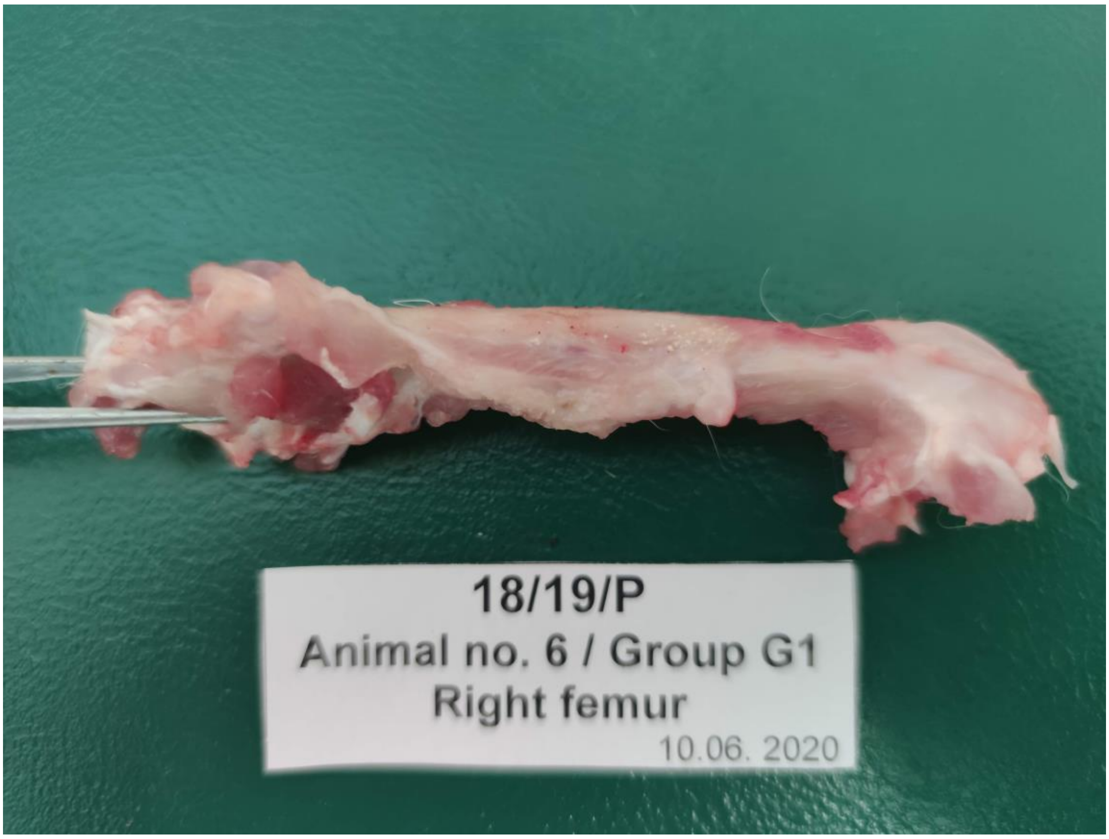

Figure 12: Animal no. 6, right femur

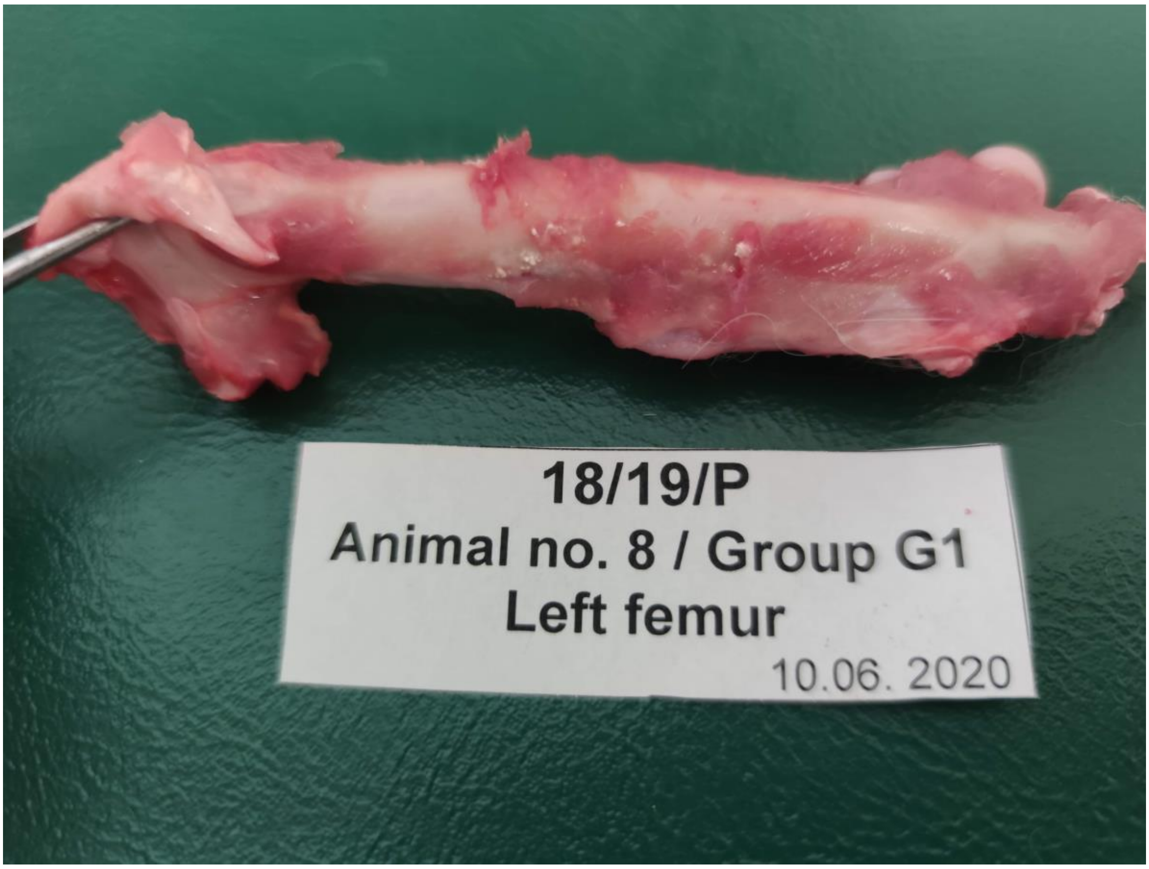

Figure 13: Animal no. 8, left femur

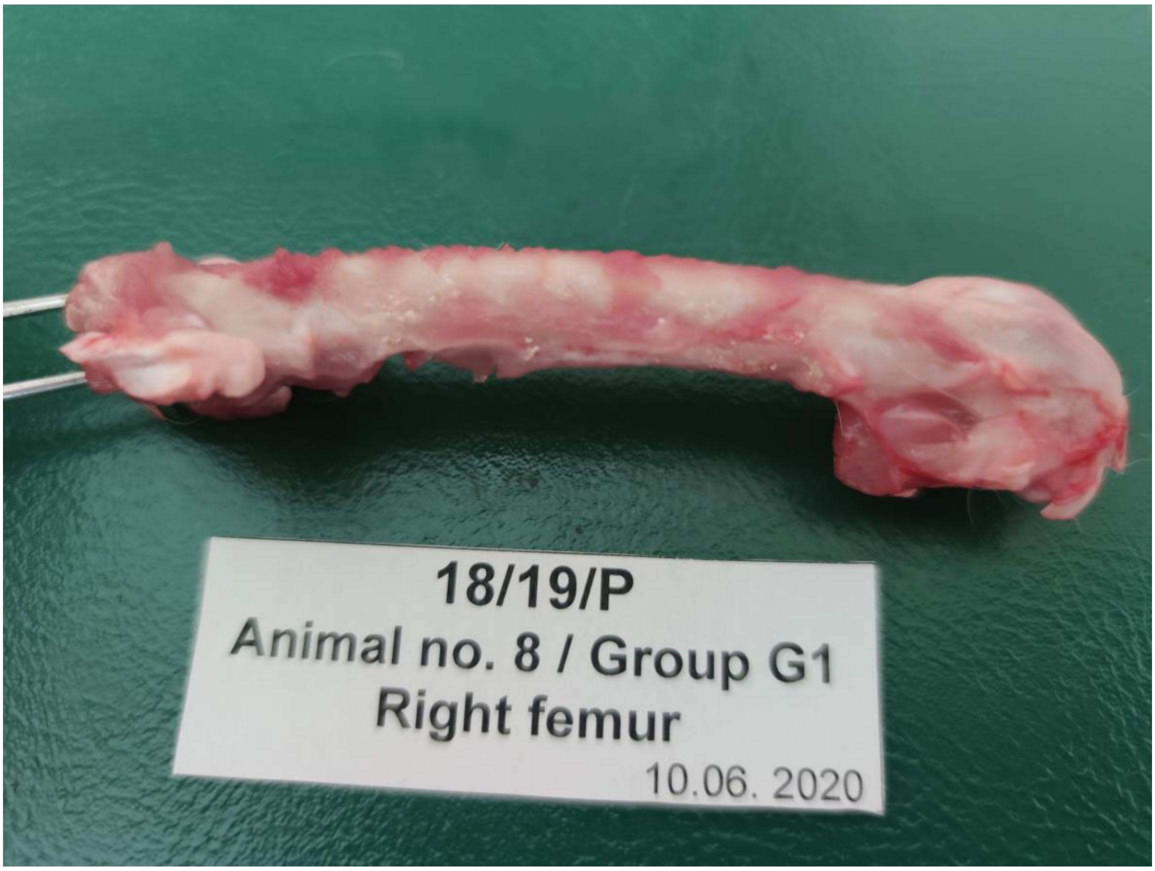

Figure 14: Animal no. 8, right femur

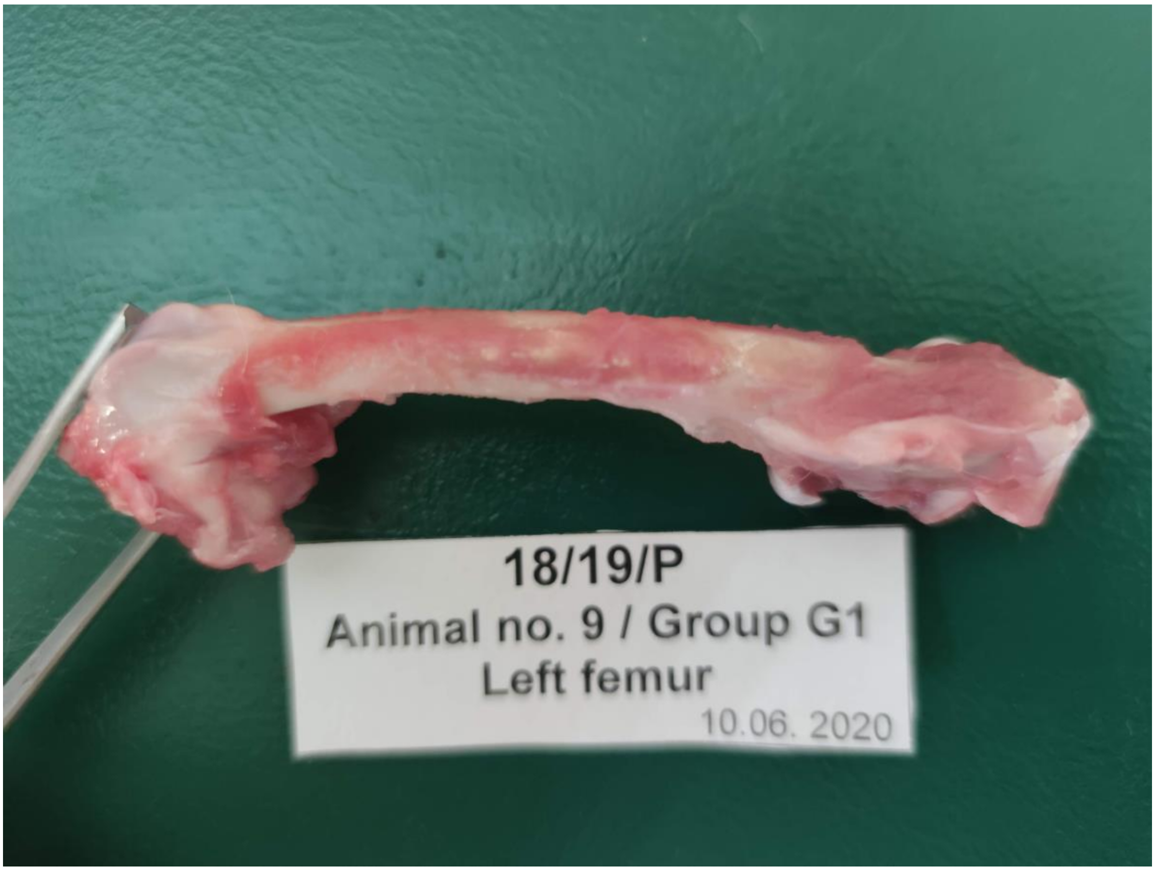

Figure 15: Animal no. 9, left femur

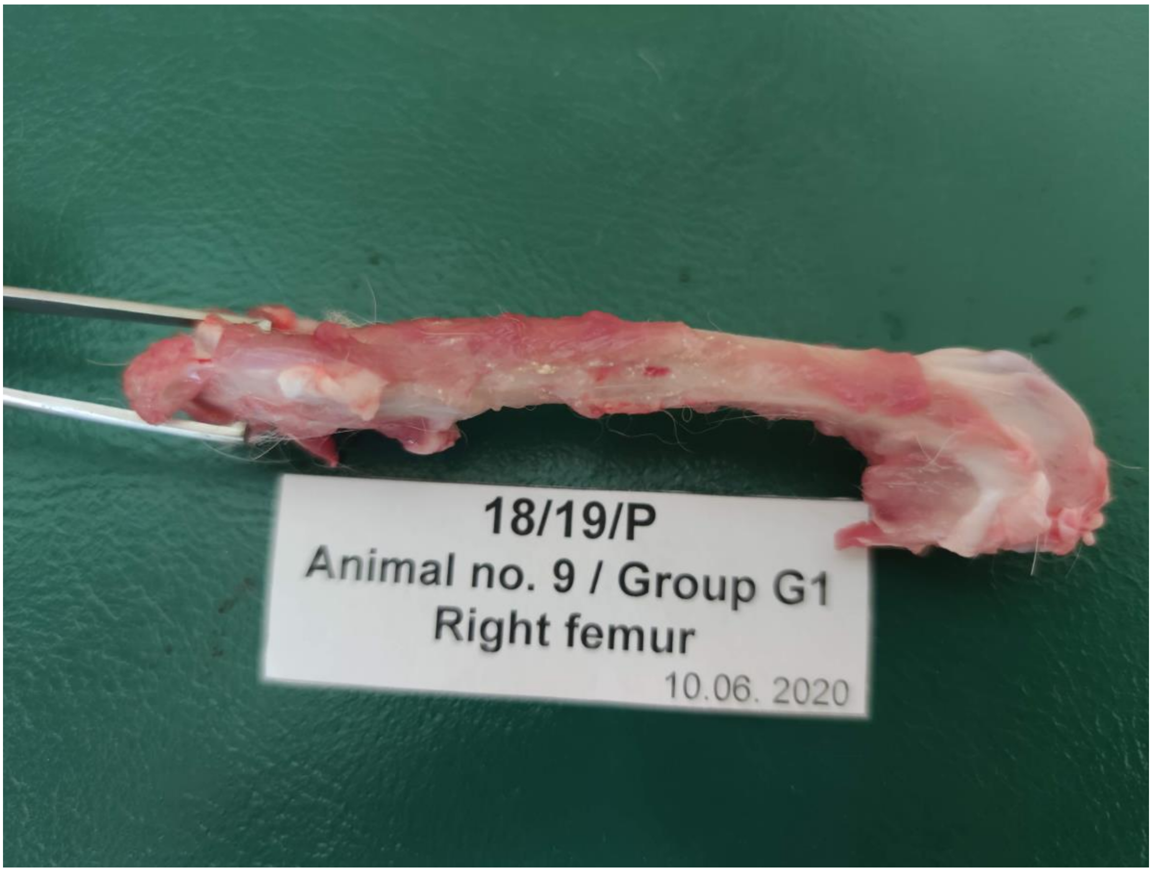

Figure 16: Animal no. 9, right femur

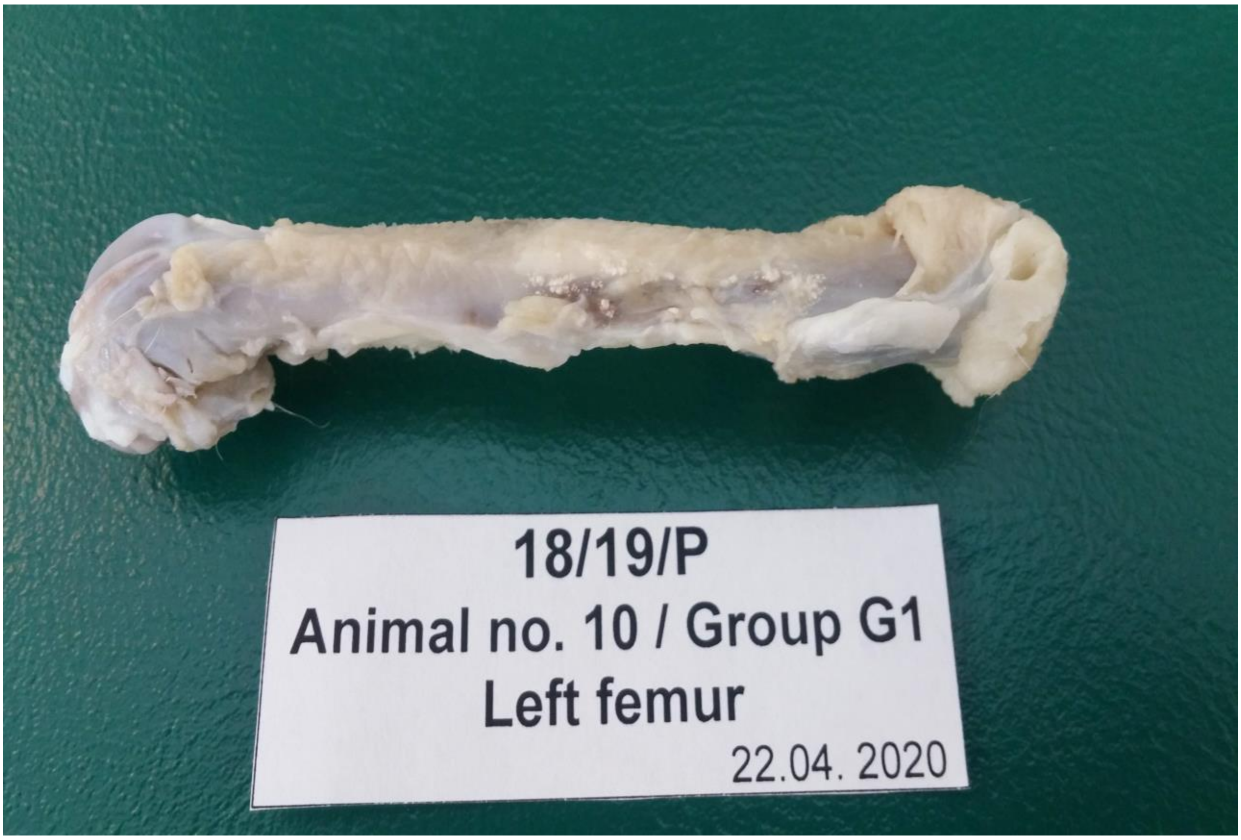

Figure 17: Animal no. 10, left femur

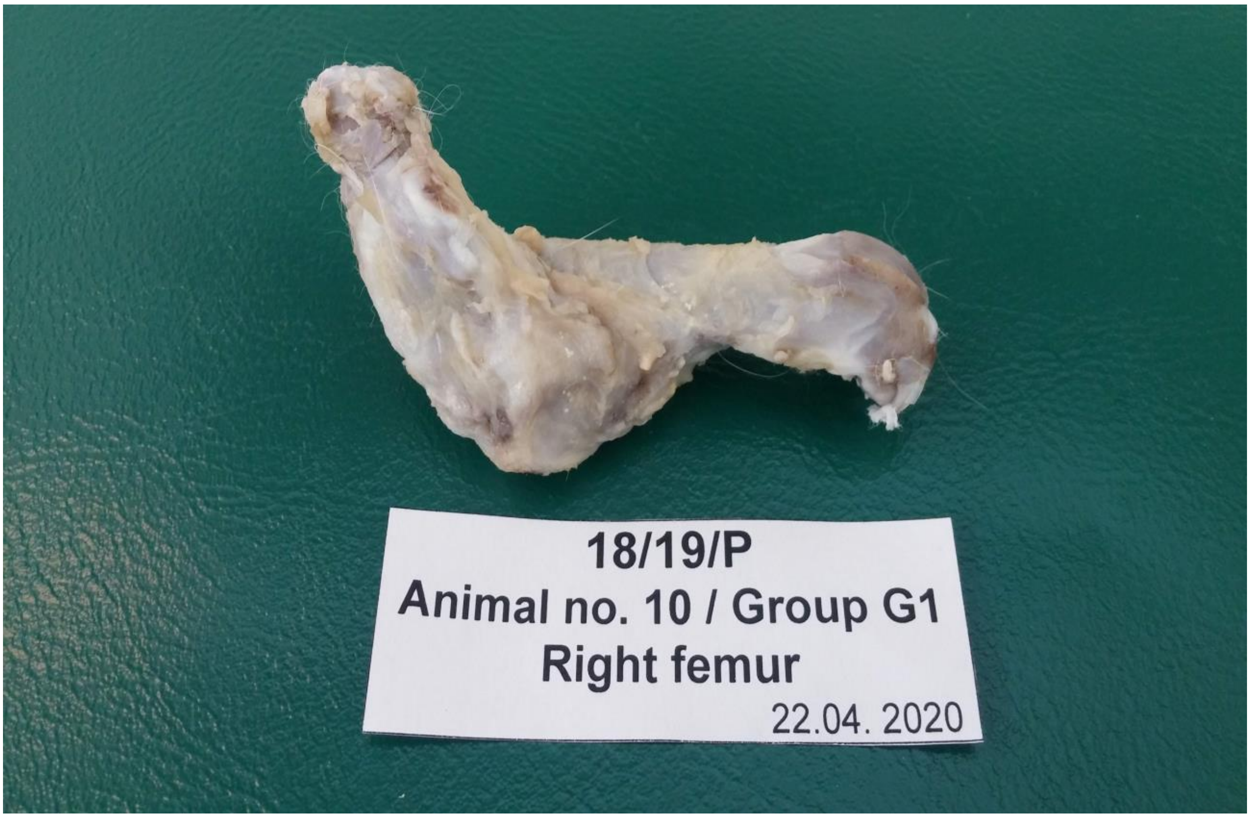

Figure 18: Animal no. 10, right femur

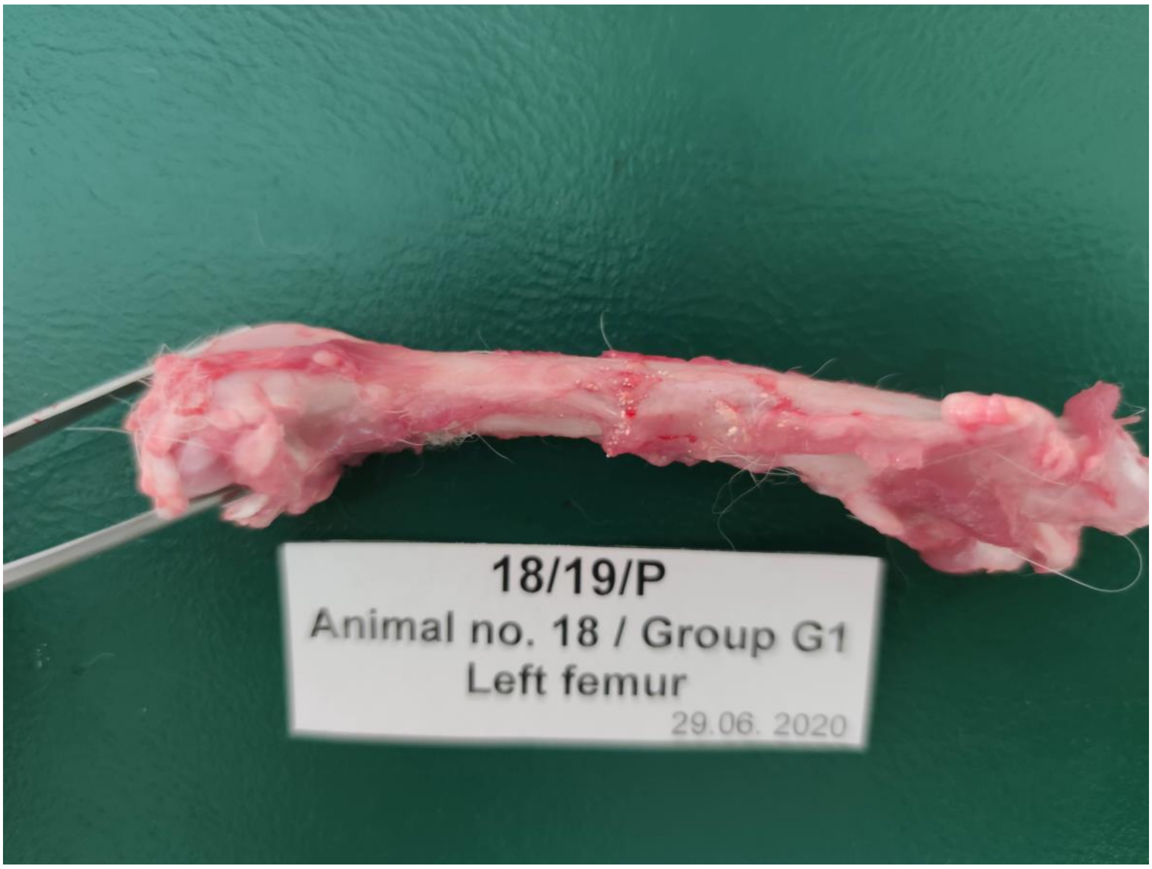

Figure 19: Animal no. 18, left femur

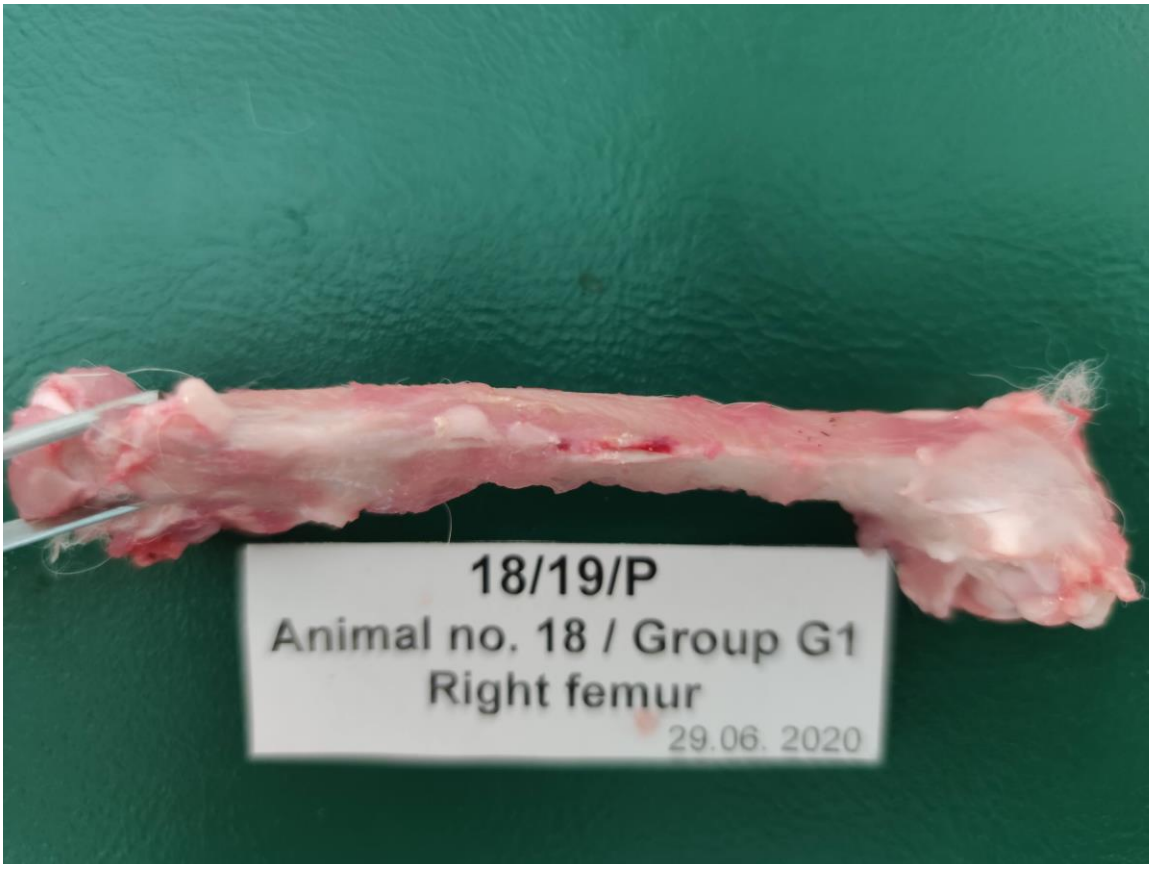

Figure 20: Animal no. 18, right femur

Photo-documentation: Group G2

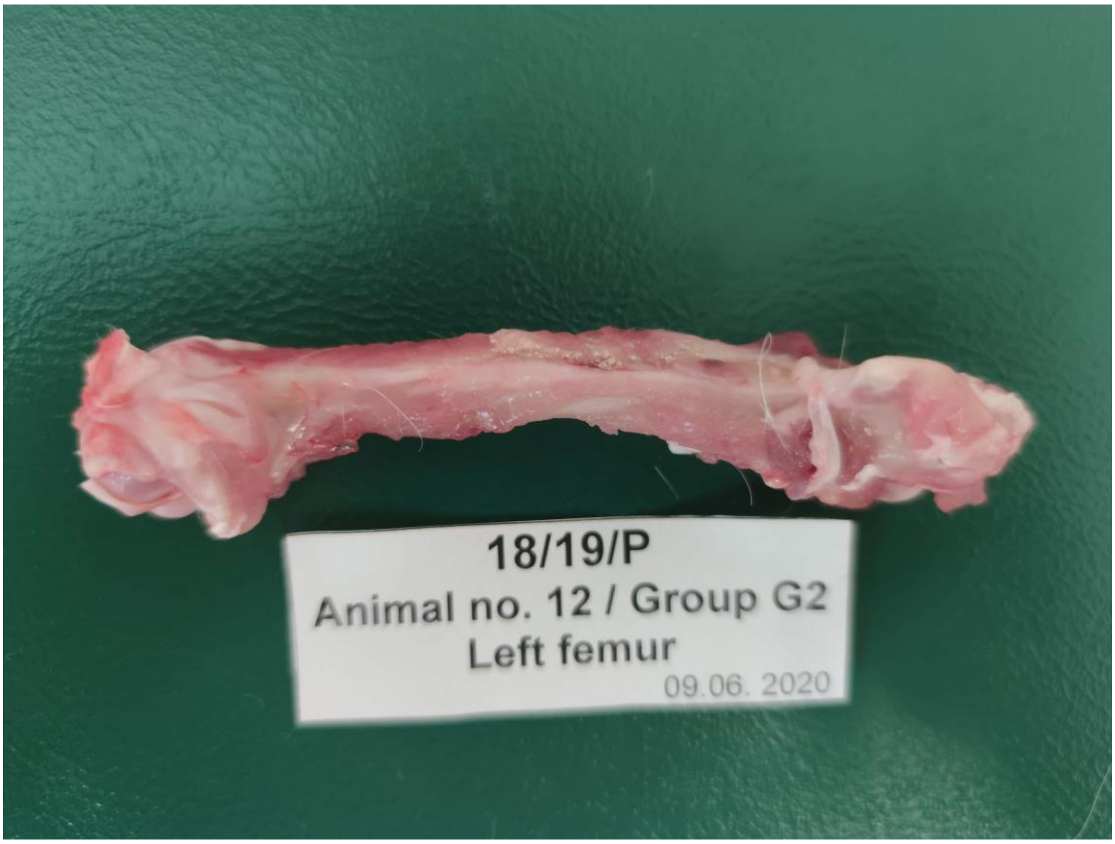

Figure 21: Animal no. 12, left femur

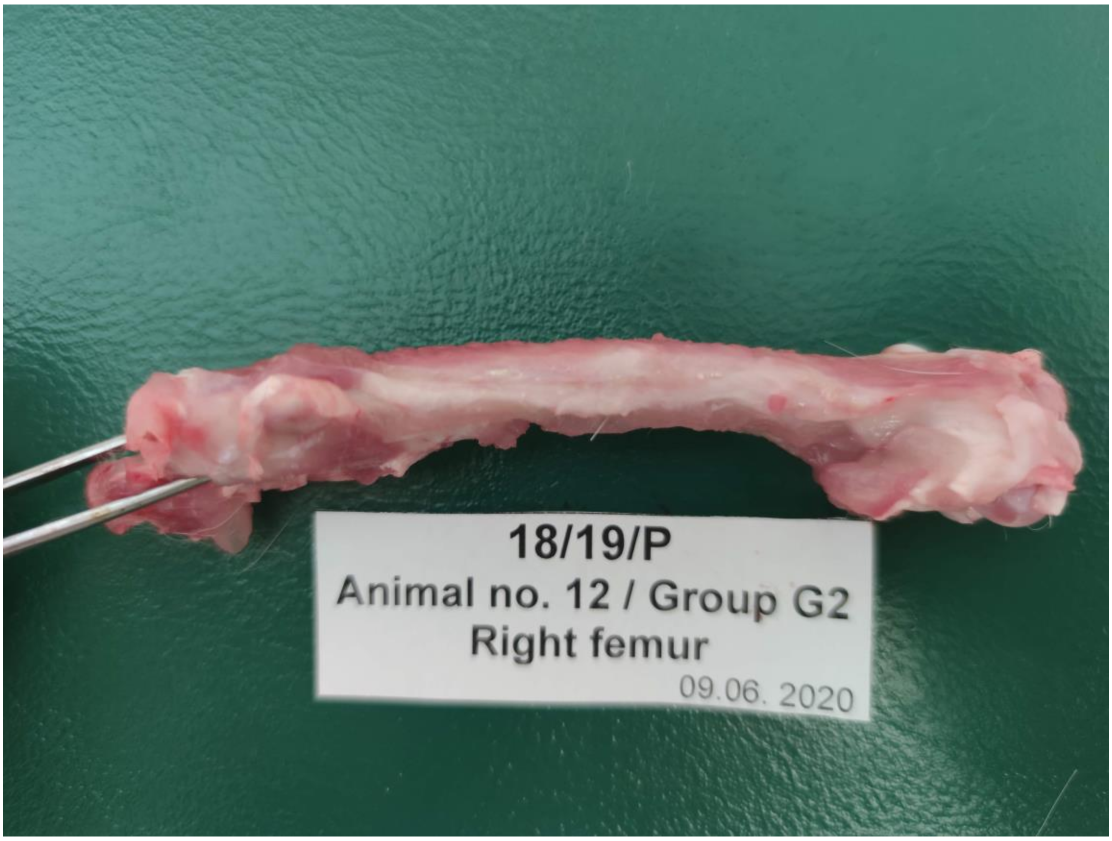

Figure 22: Animal no. 12, right femur

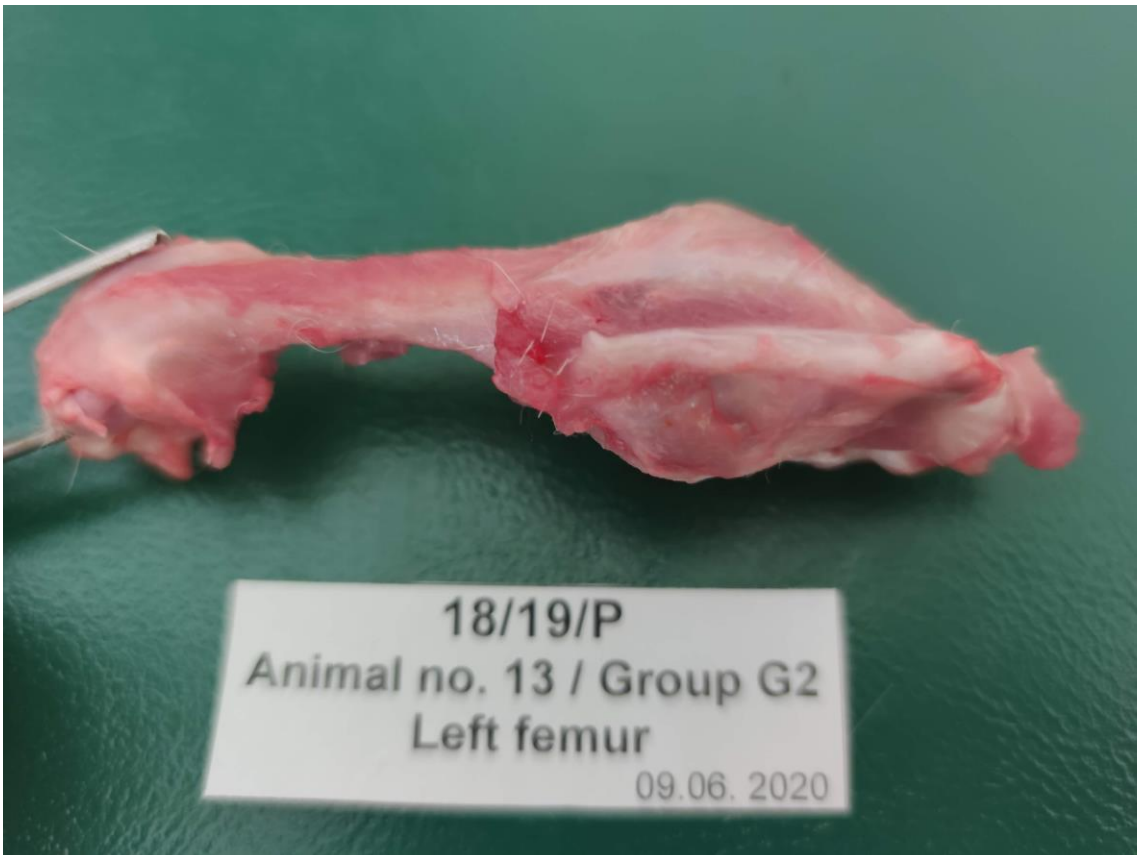

Figure 23: Animal no. 13, left femur

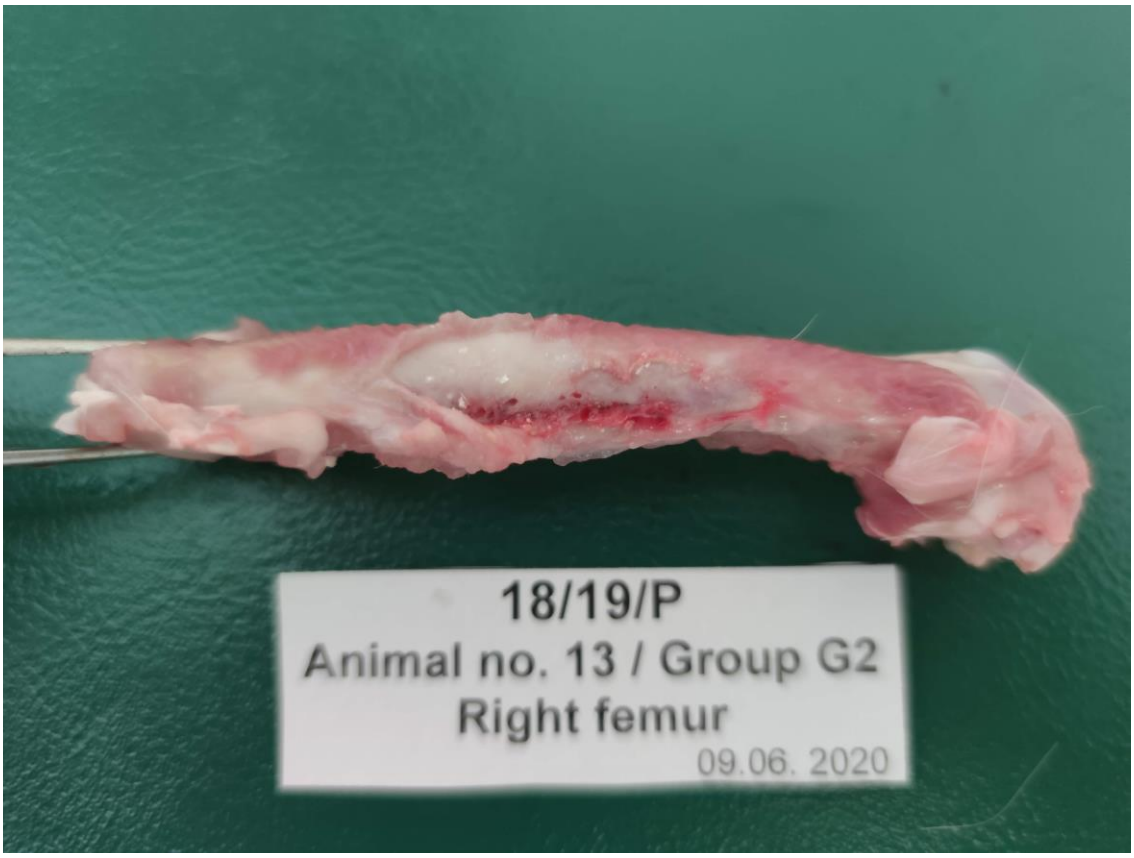

Figure 24: Animal no. 13, right femur

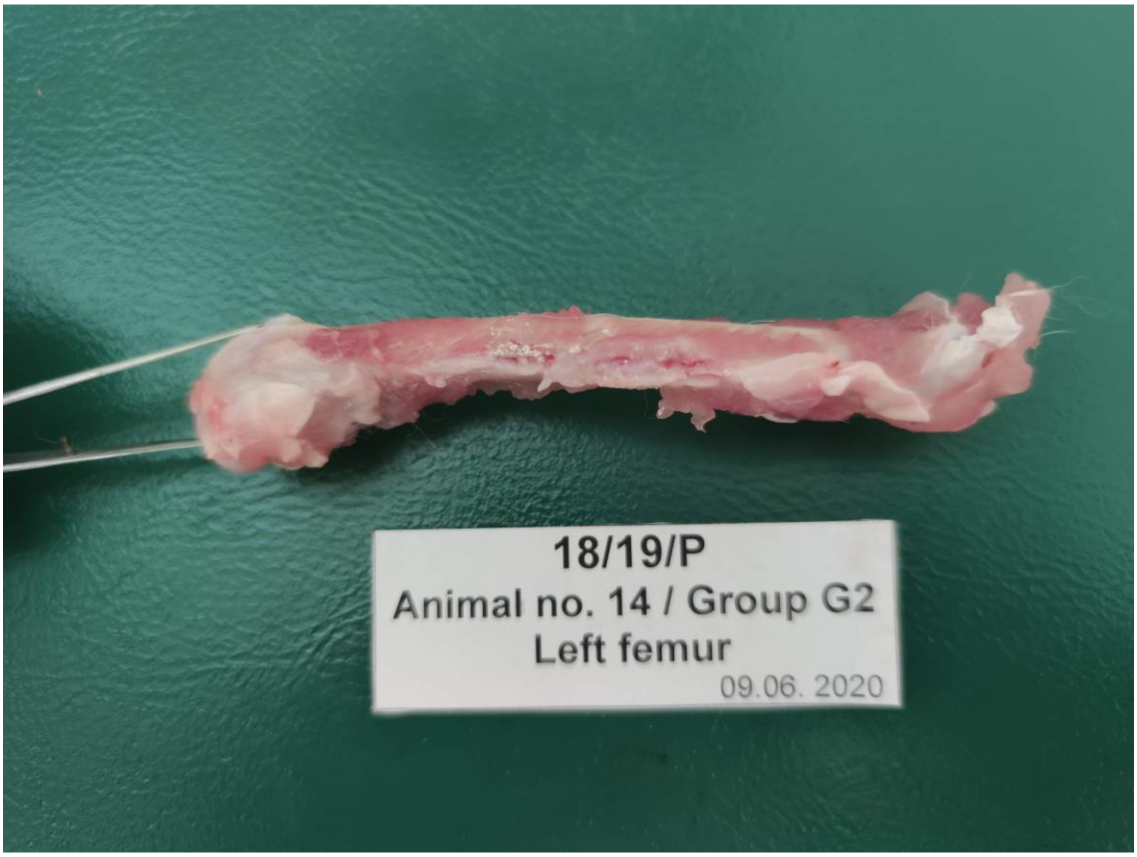

Figure 25: Animal no. 14, left femur

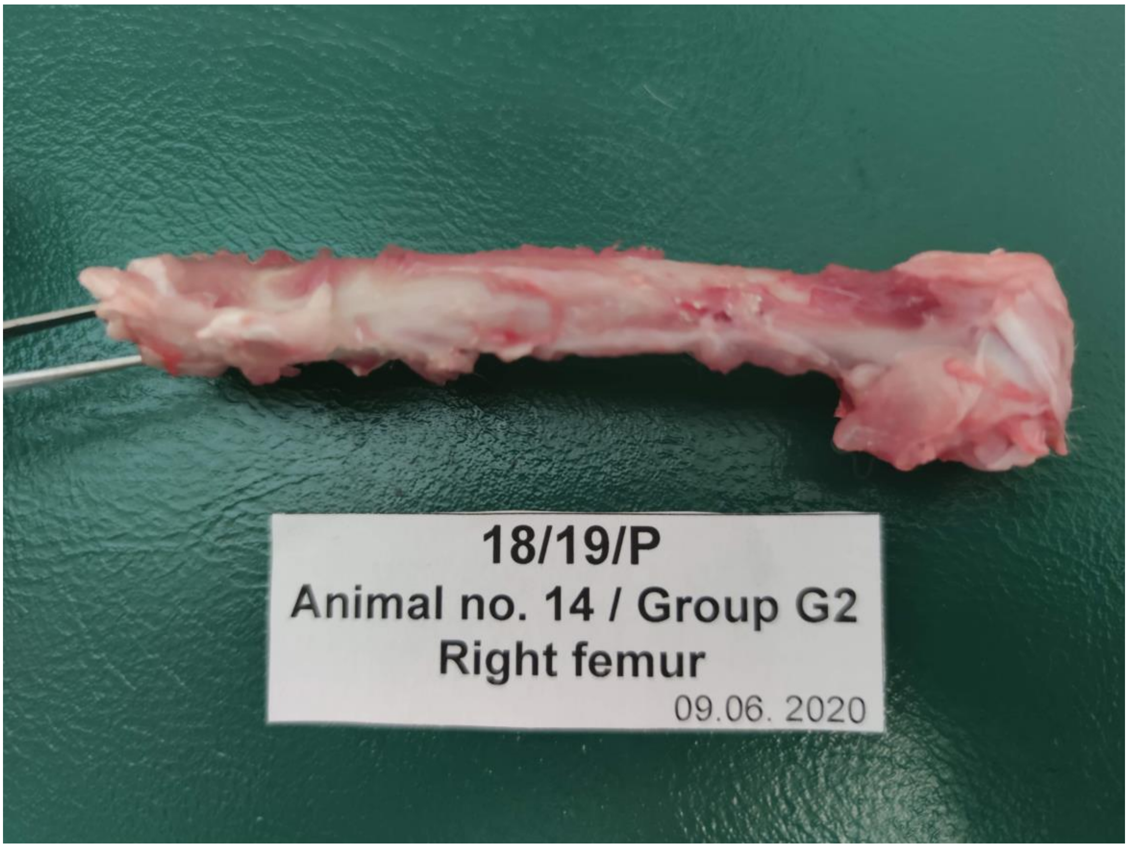

Figure 26: Animal no. 14, right femur

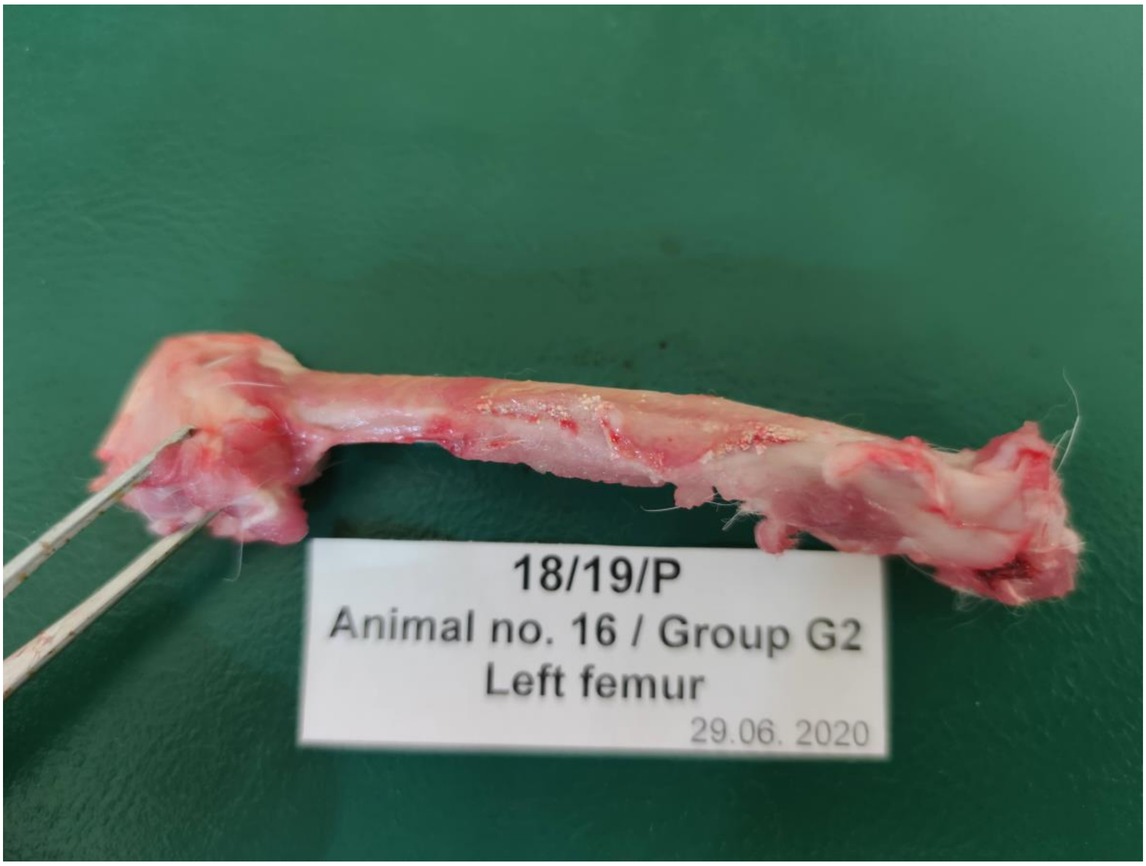

Figure 27: Animal no. 16, left femur

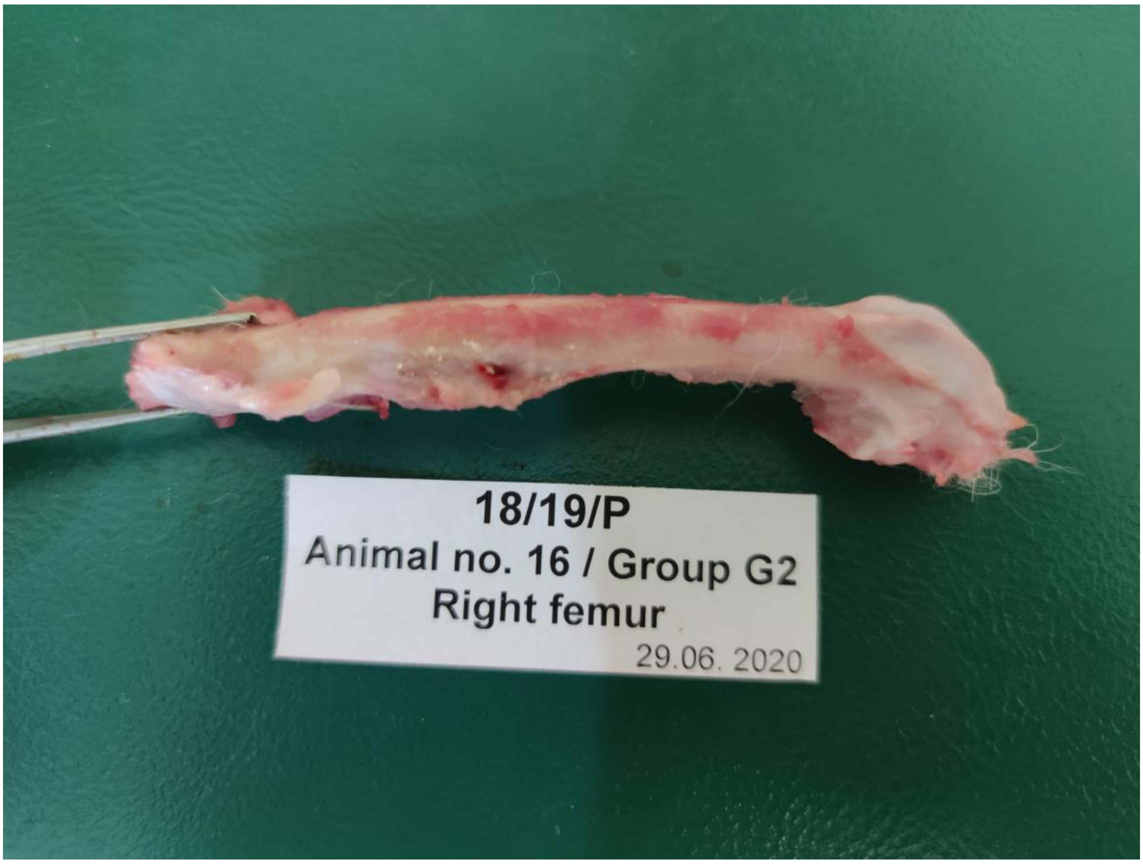

Figure 28: Animal no. 16, right femur

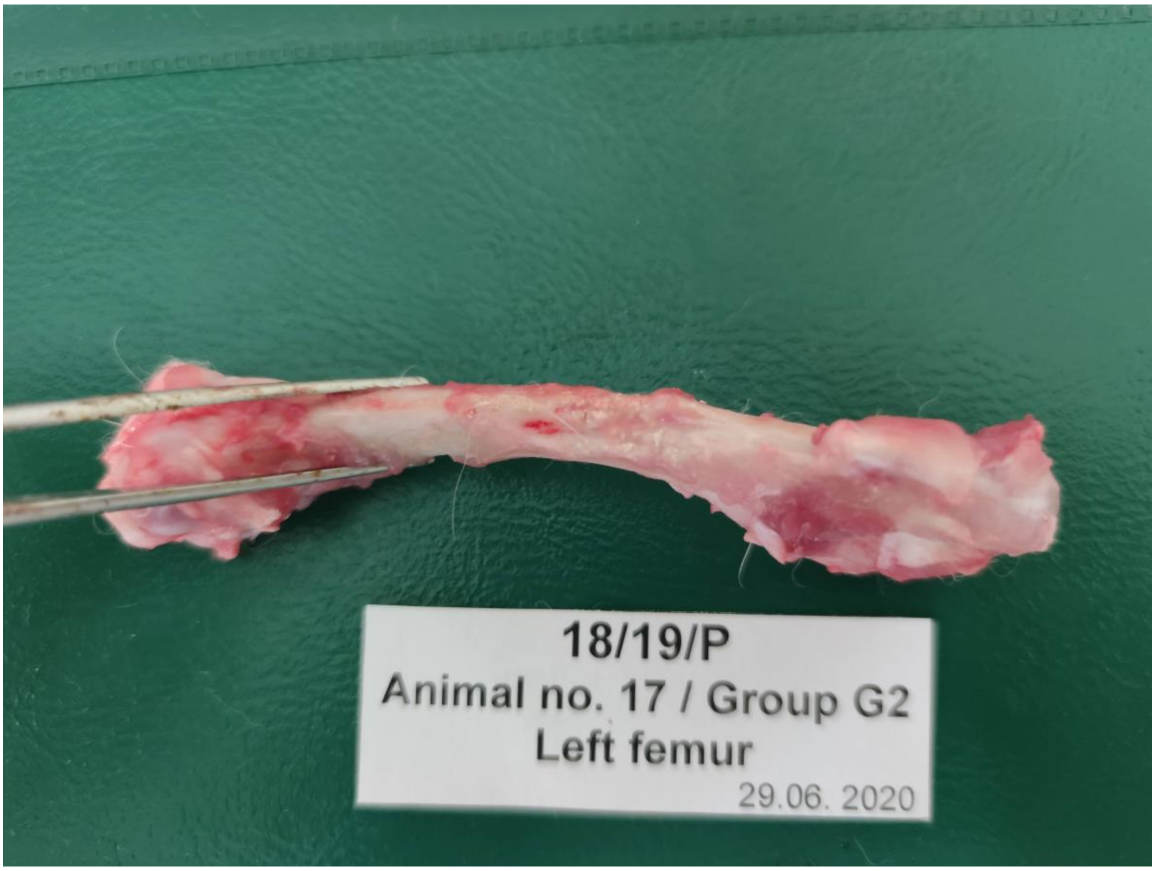

Figure 29: Animal no. 17, left femur

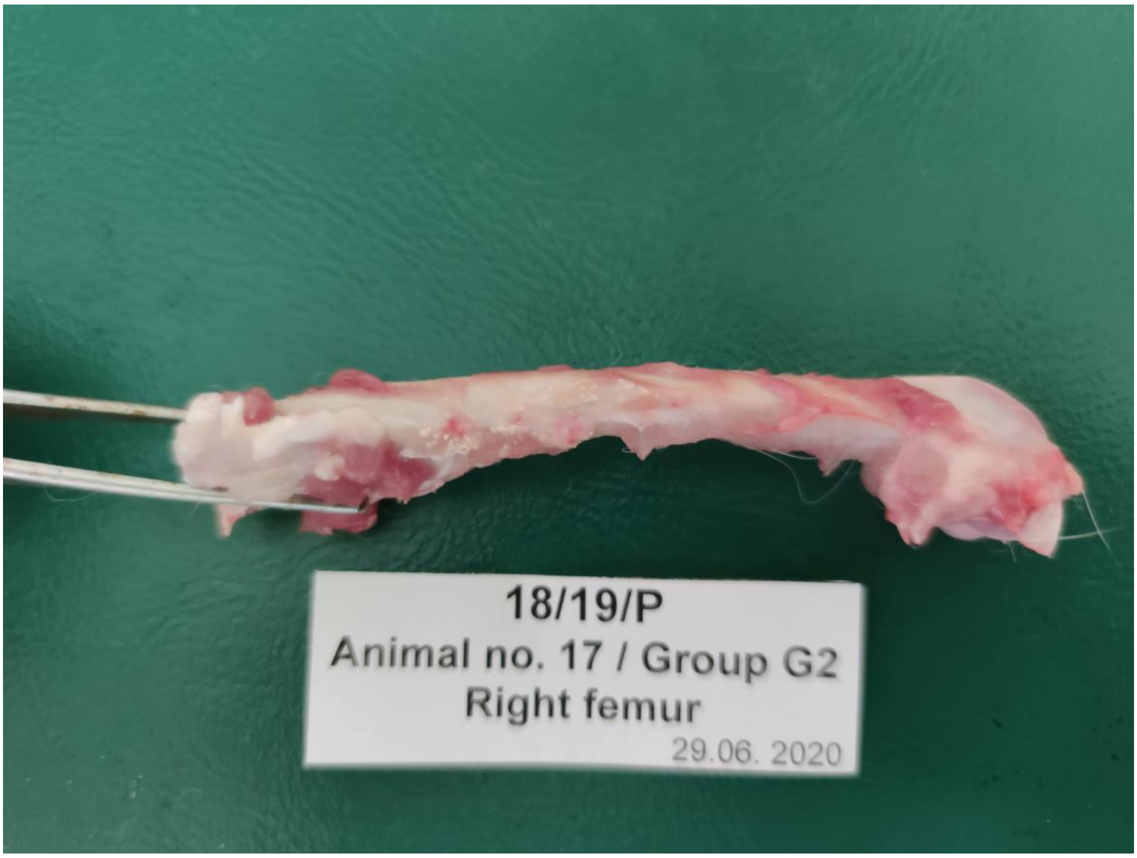

Figure 30: Animal no. 17, right femur

X-Ray photos: Control Group C

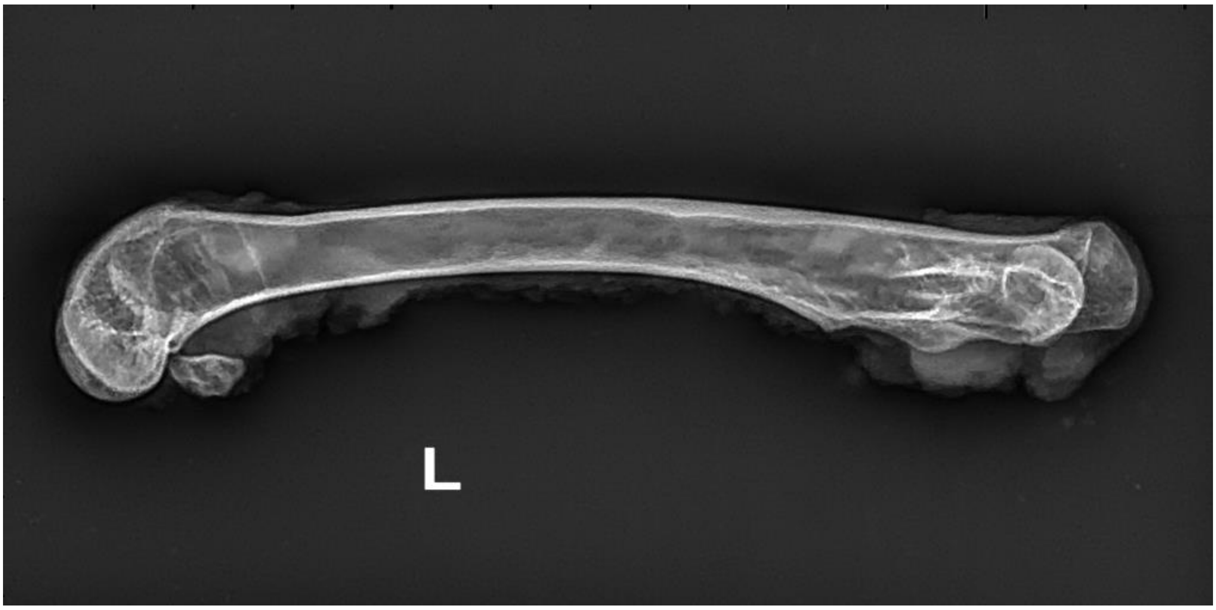

Figure 31: Animal no. 1, left femur

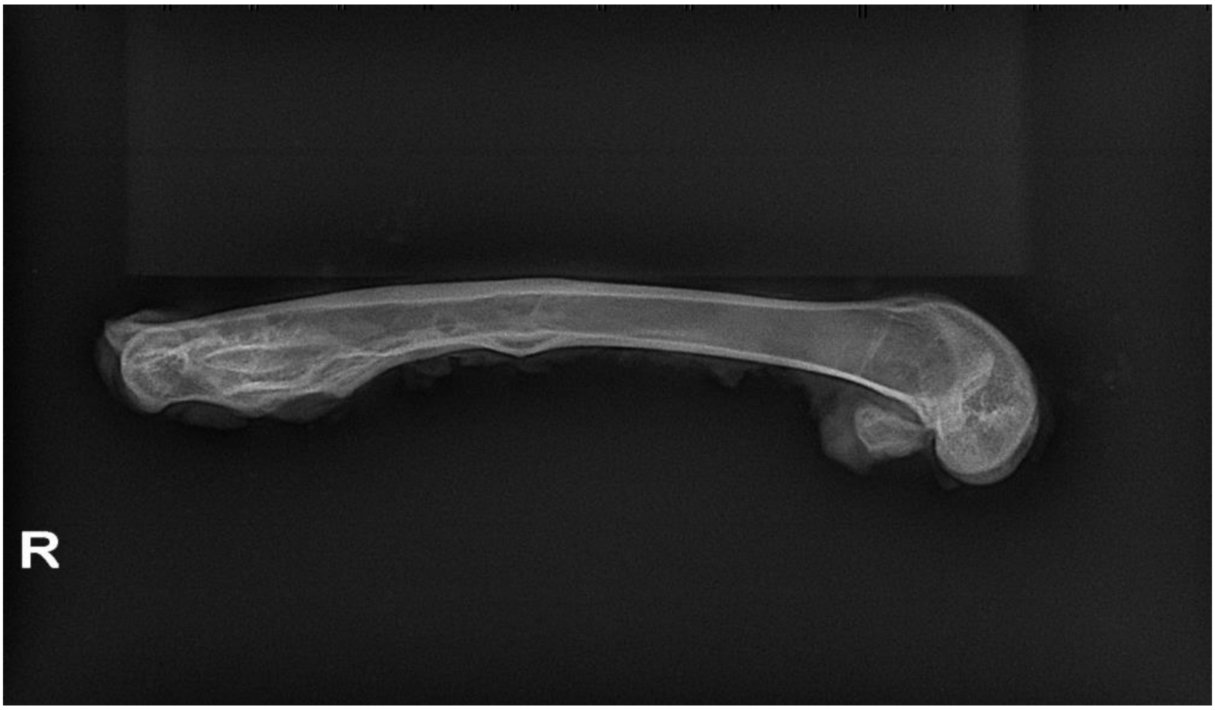

Figure 32: Animal no. 1, right femur

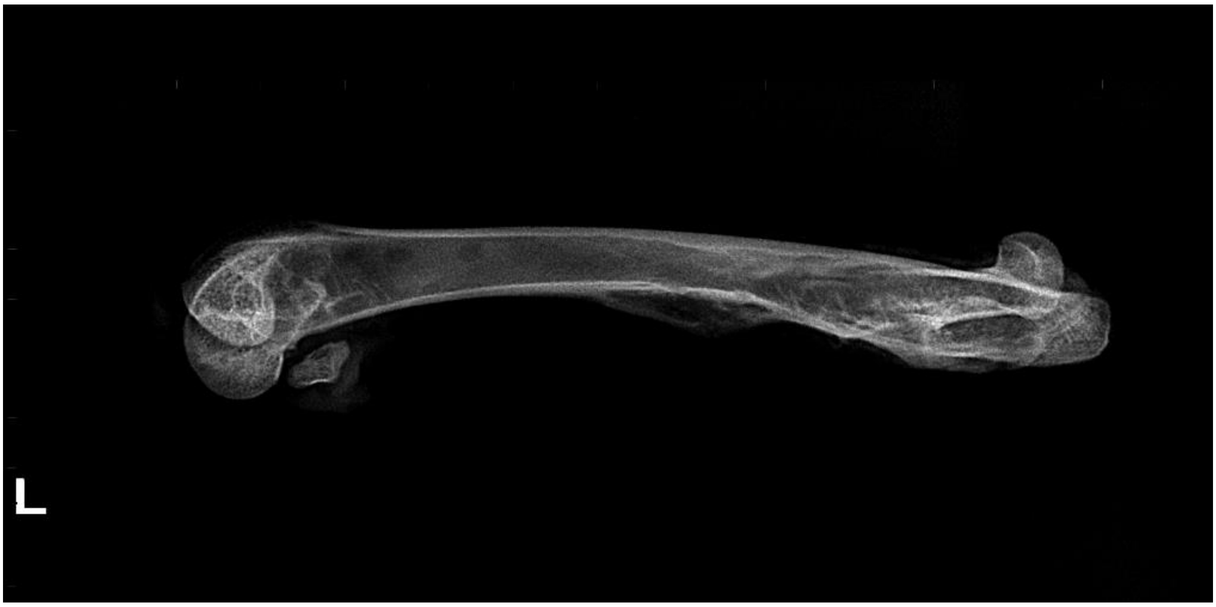

Figure 33: Animal no. 2, left femur

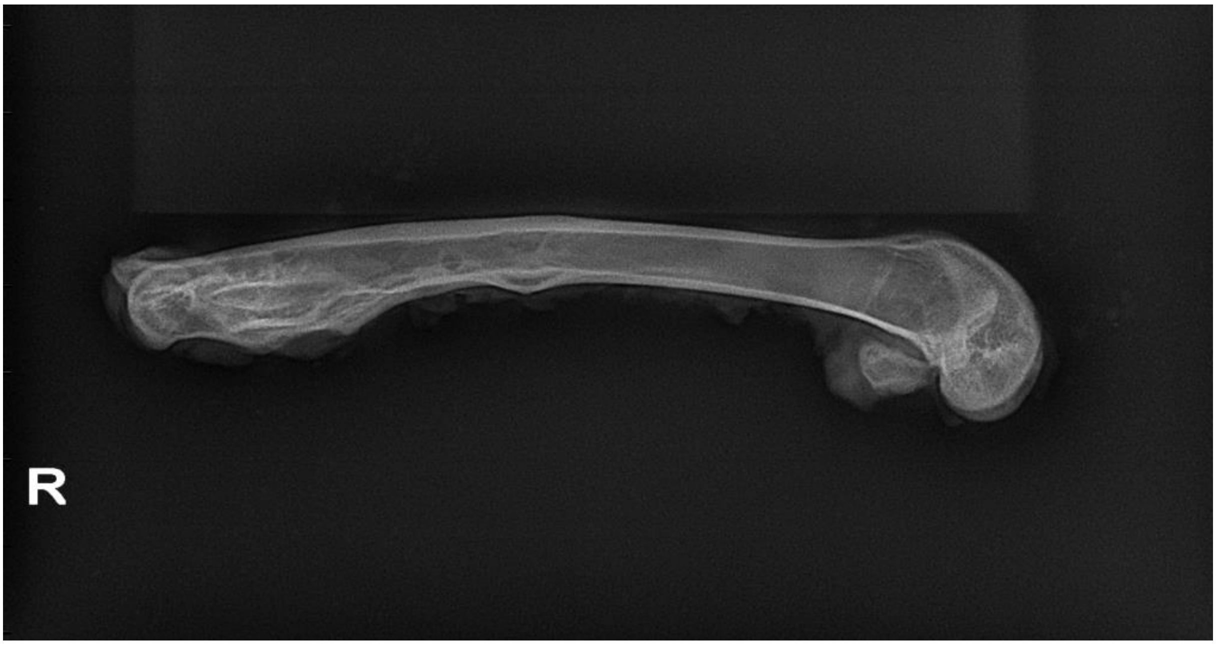

Figure 34: Animal no. 2, right femur

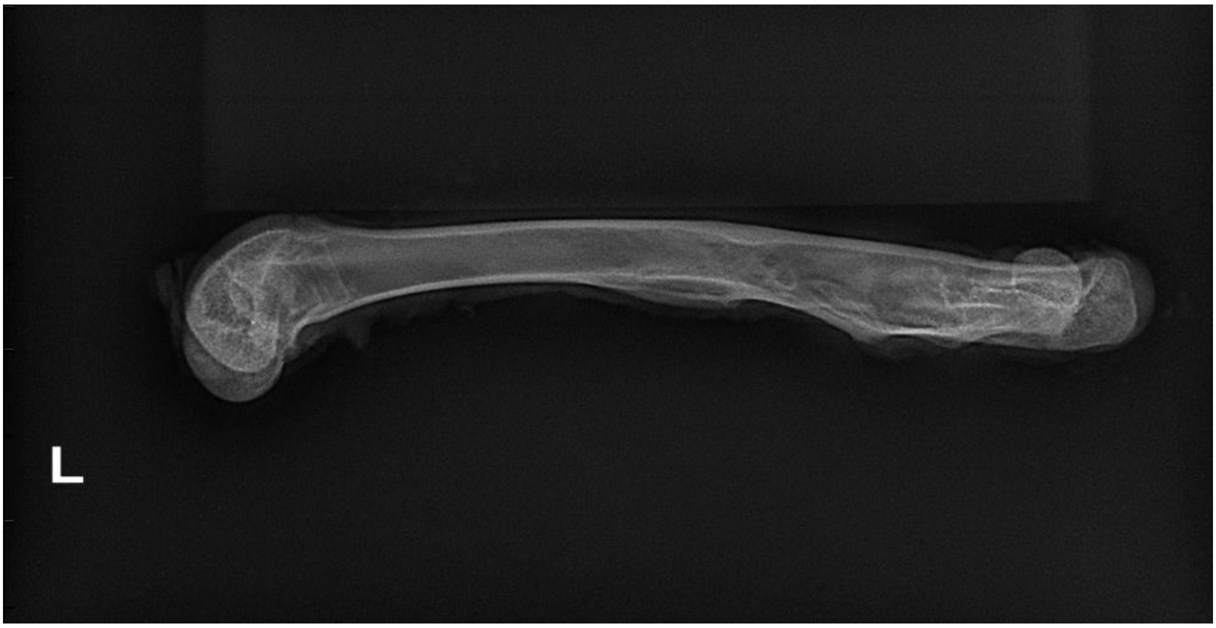

Figure 35: Animal no. 3, left femur

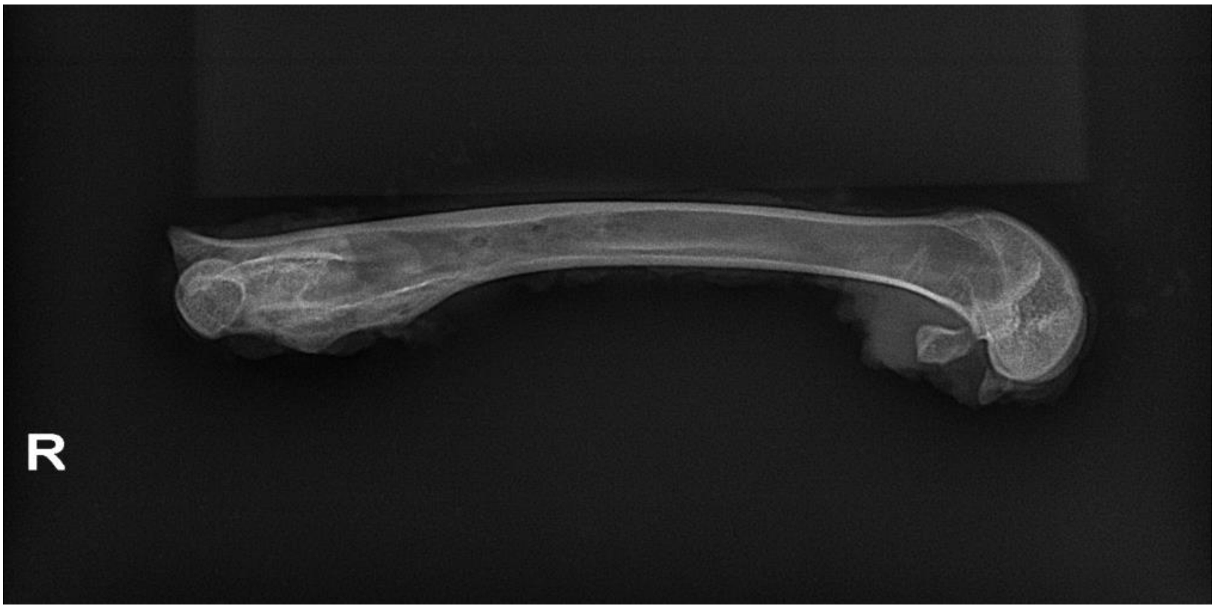

Figure 36: Animal no. 3, right femur

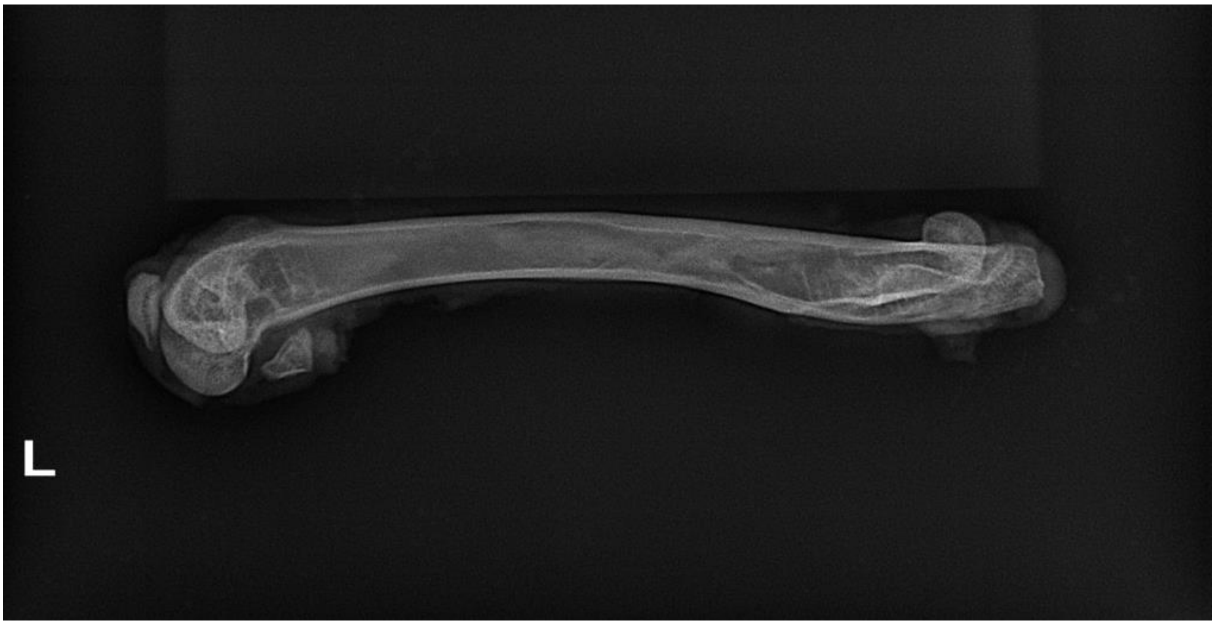

Figure 37: Animal no. 4, left femur

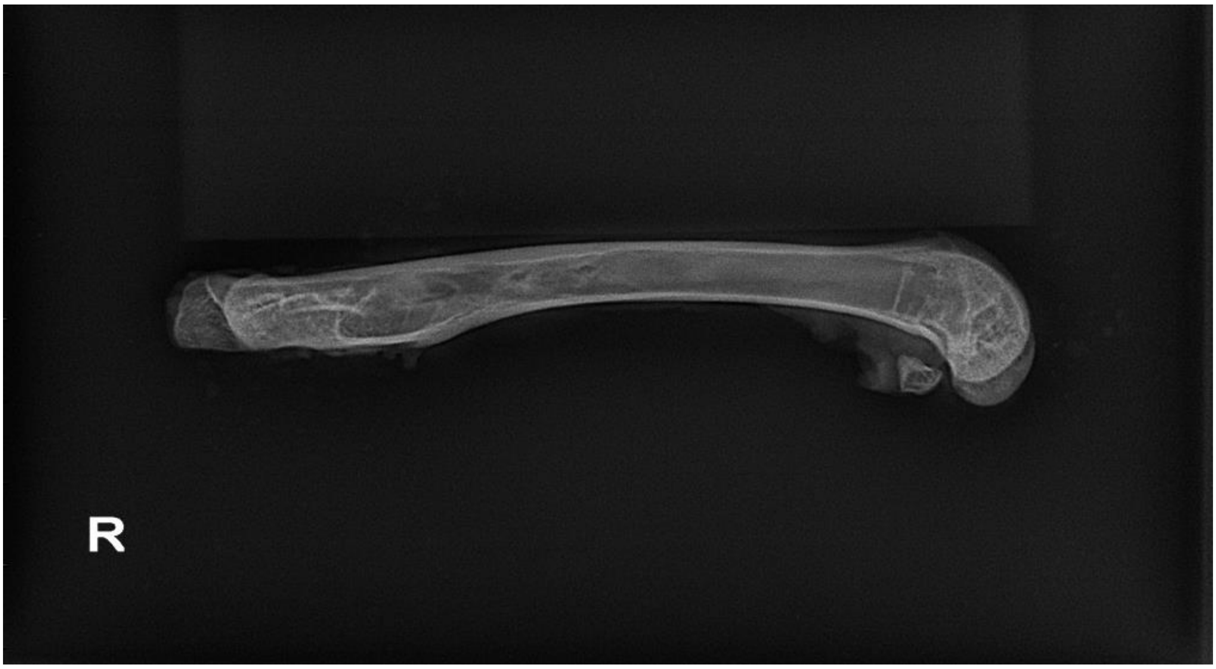

Figure 38: Animal no. 4, right femur

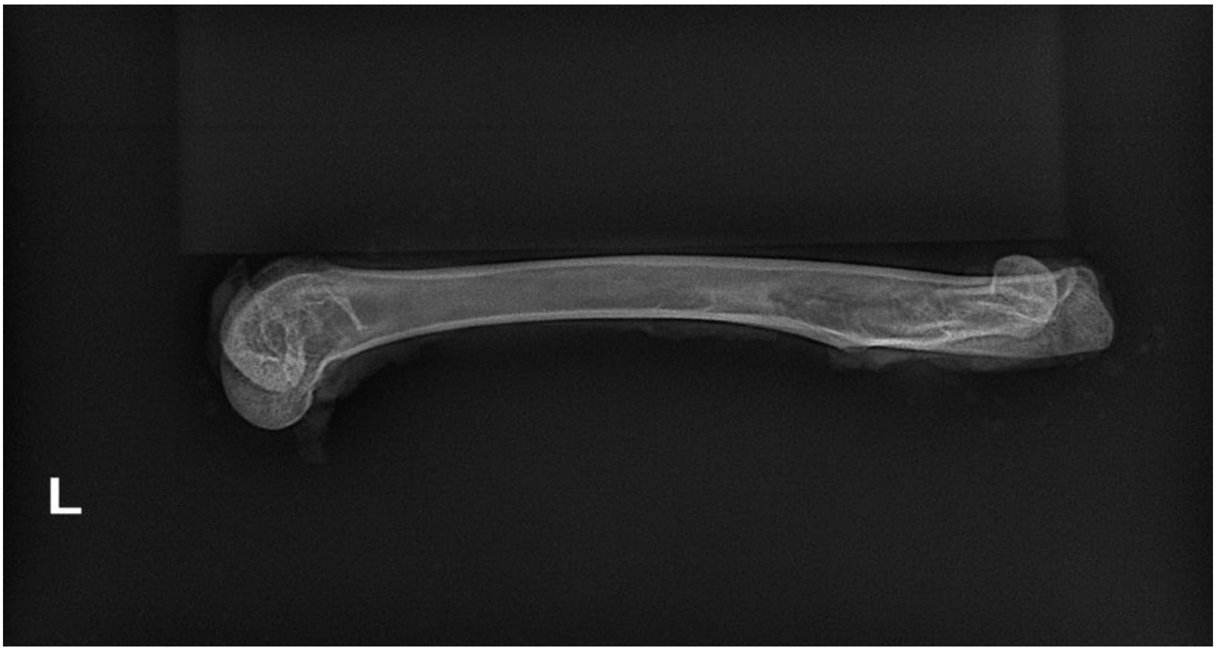

Figure 39: Animal no. 5, left femur

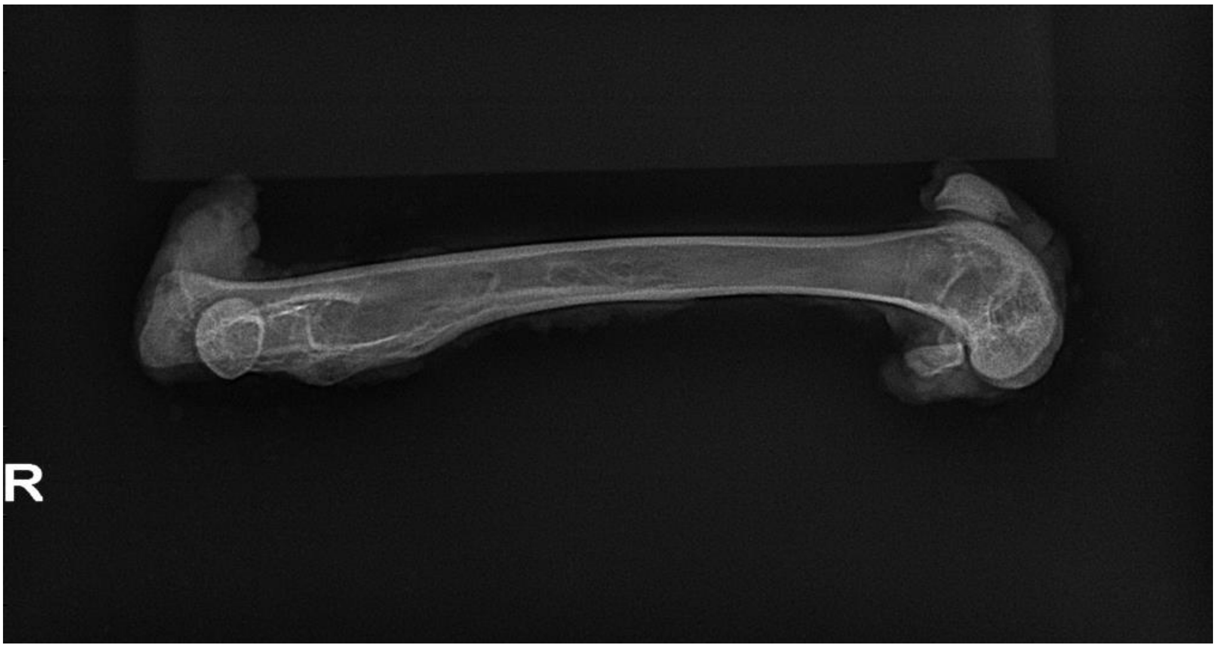

Figure 40: Animal no. 5, right femur

X-Ray photos: Group G1

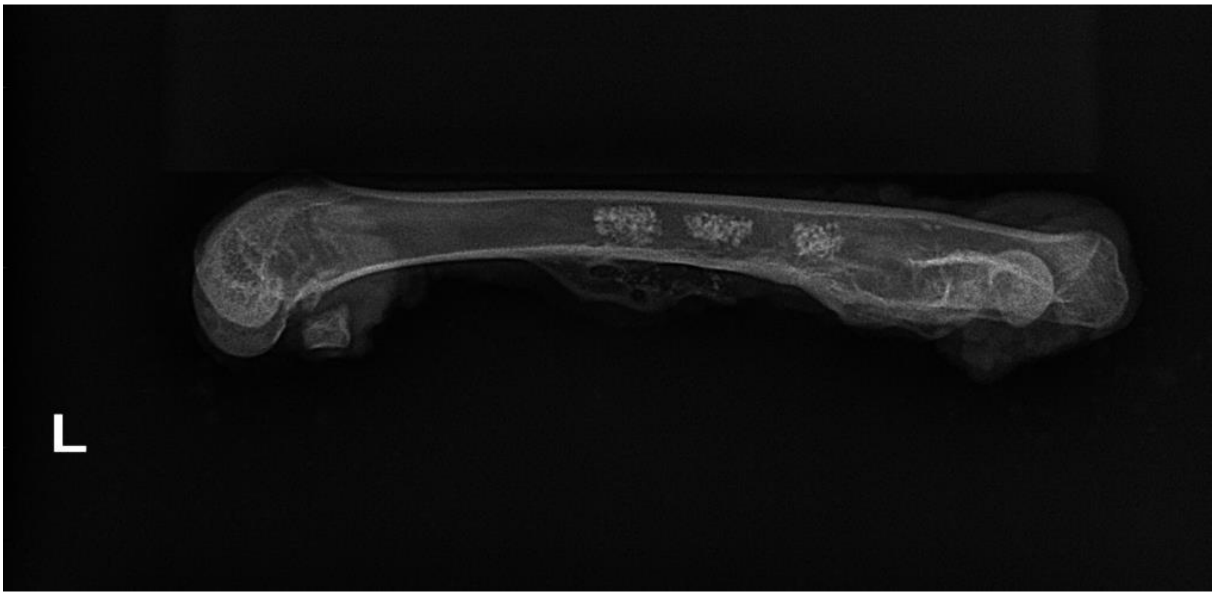

Figure 41: Animal no. 6, left femur

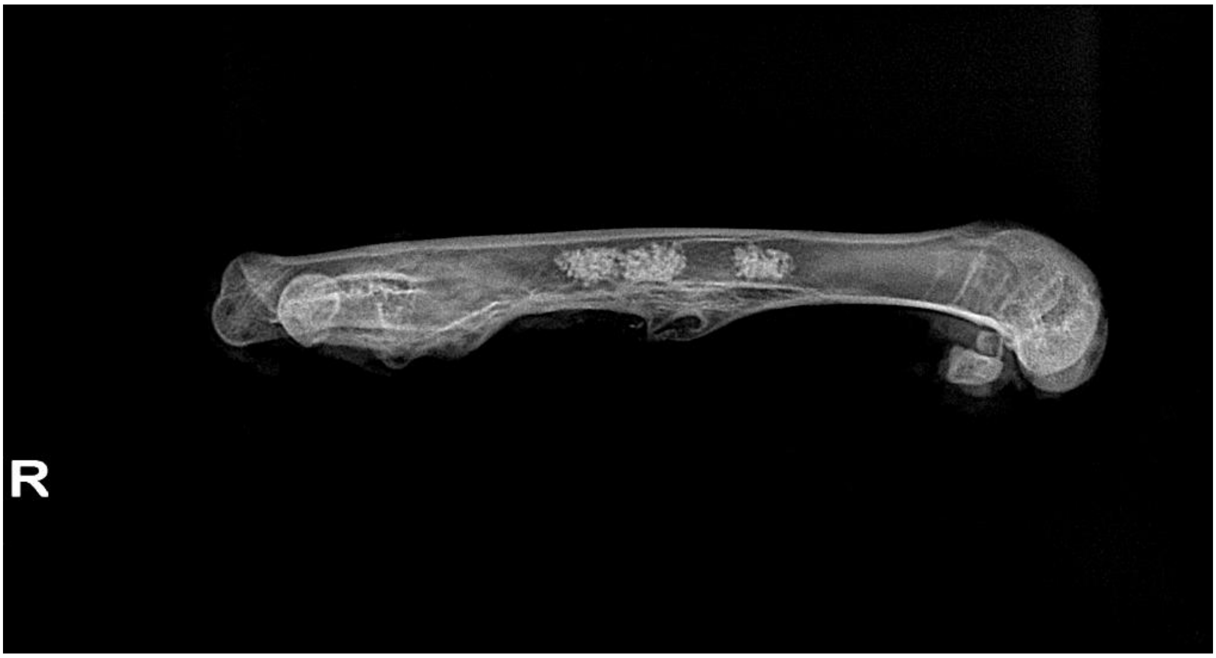

Figure 42: Animal no. 6, right femur

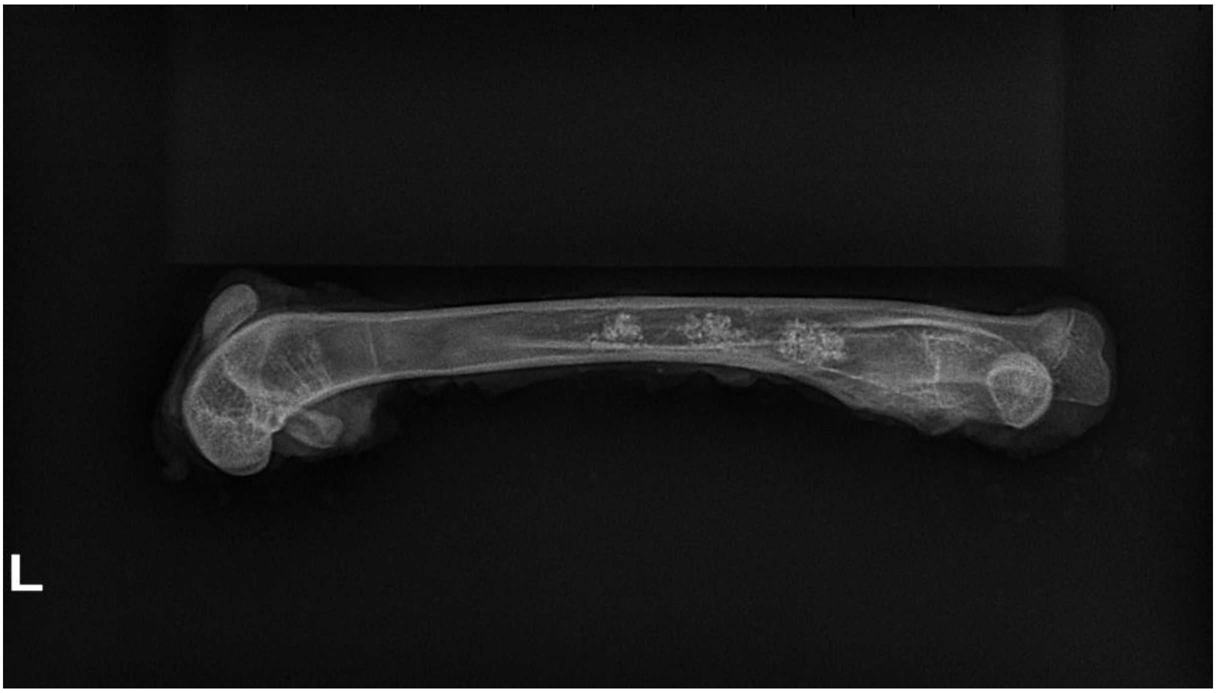

Figure 43: Animal no. 8, left femur

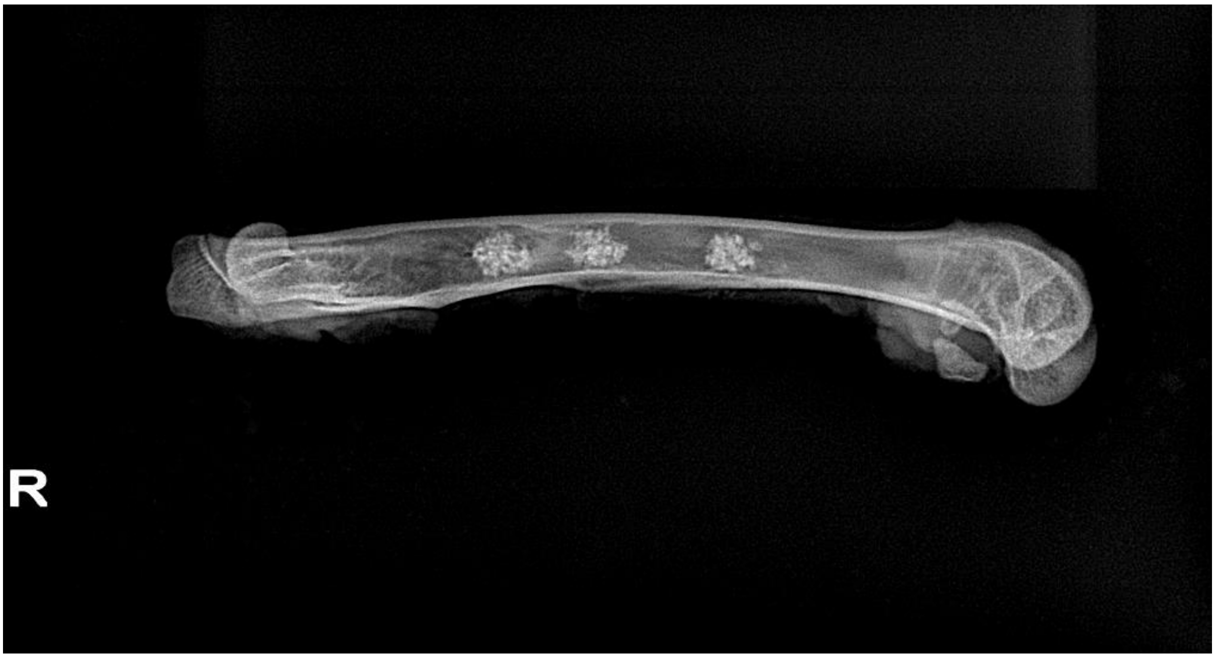

Figure 44: Animal no. 8, right femur

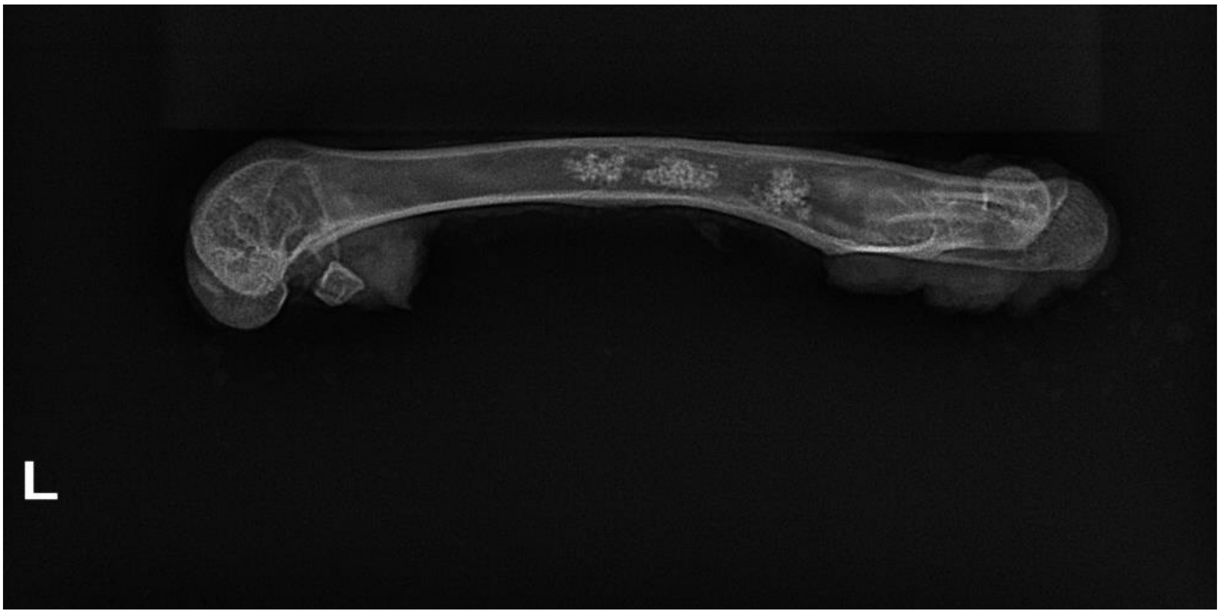

Figure 45: Animal no. 9, left femur

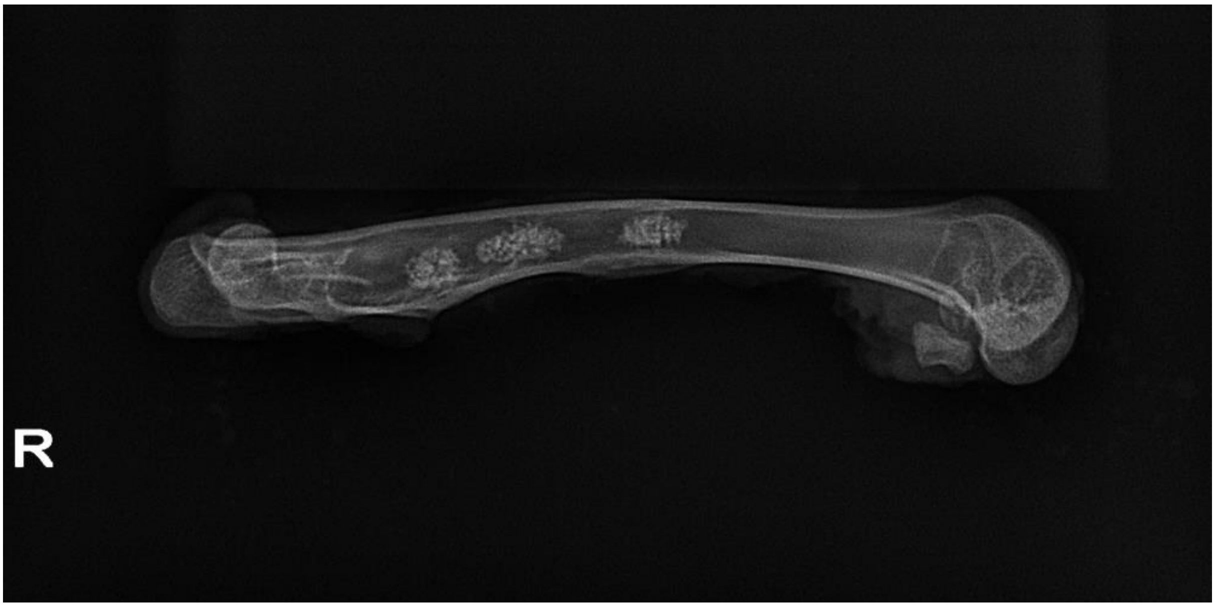

Figure 46: Animal no. 9, right femur

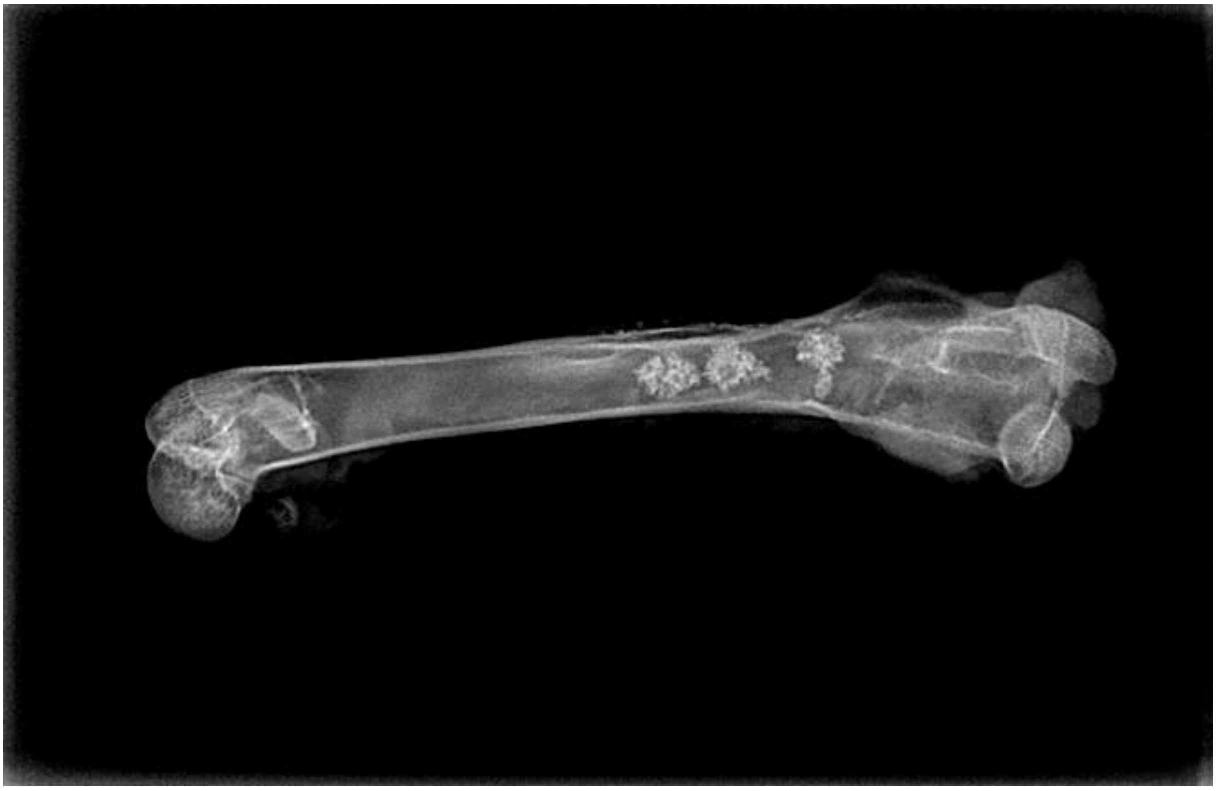

Figure 47: Animal no. 10, left femur

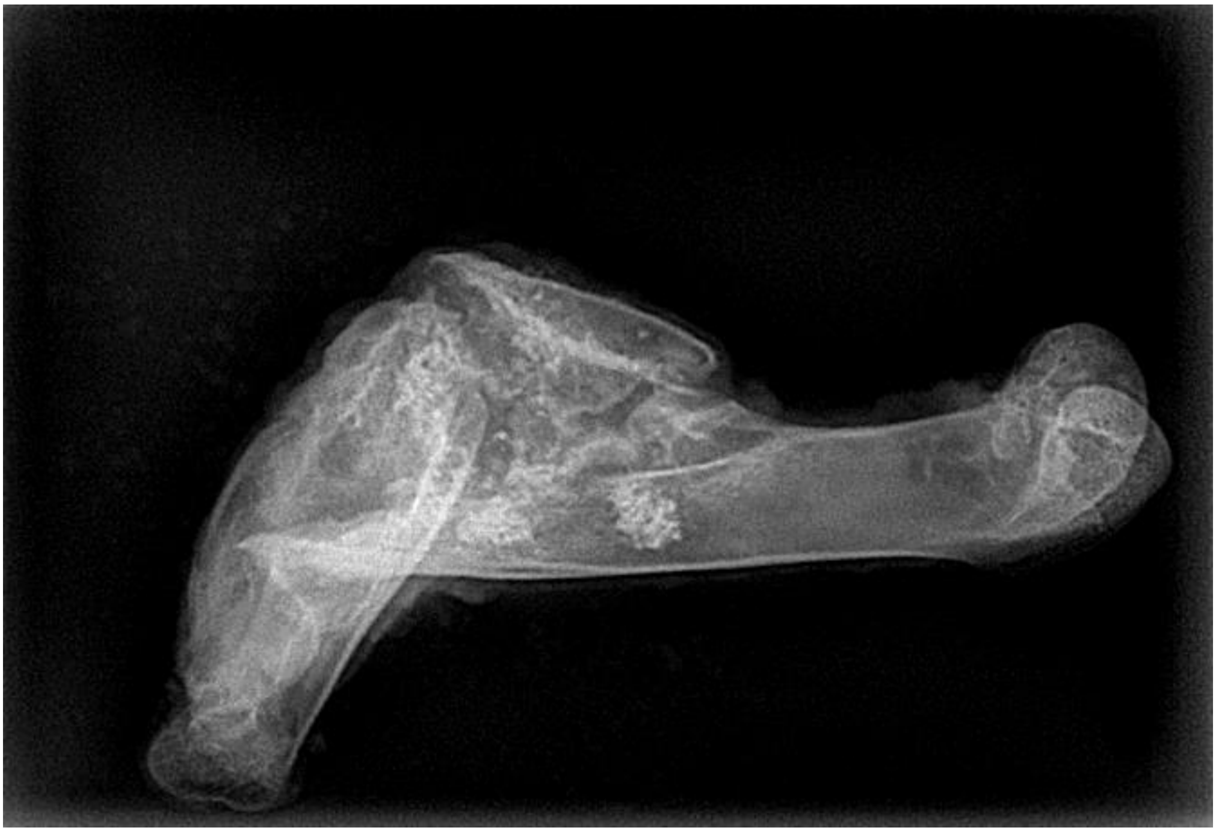

Figure 48: Animal no. 10, right femur

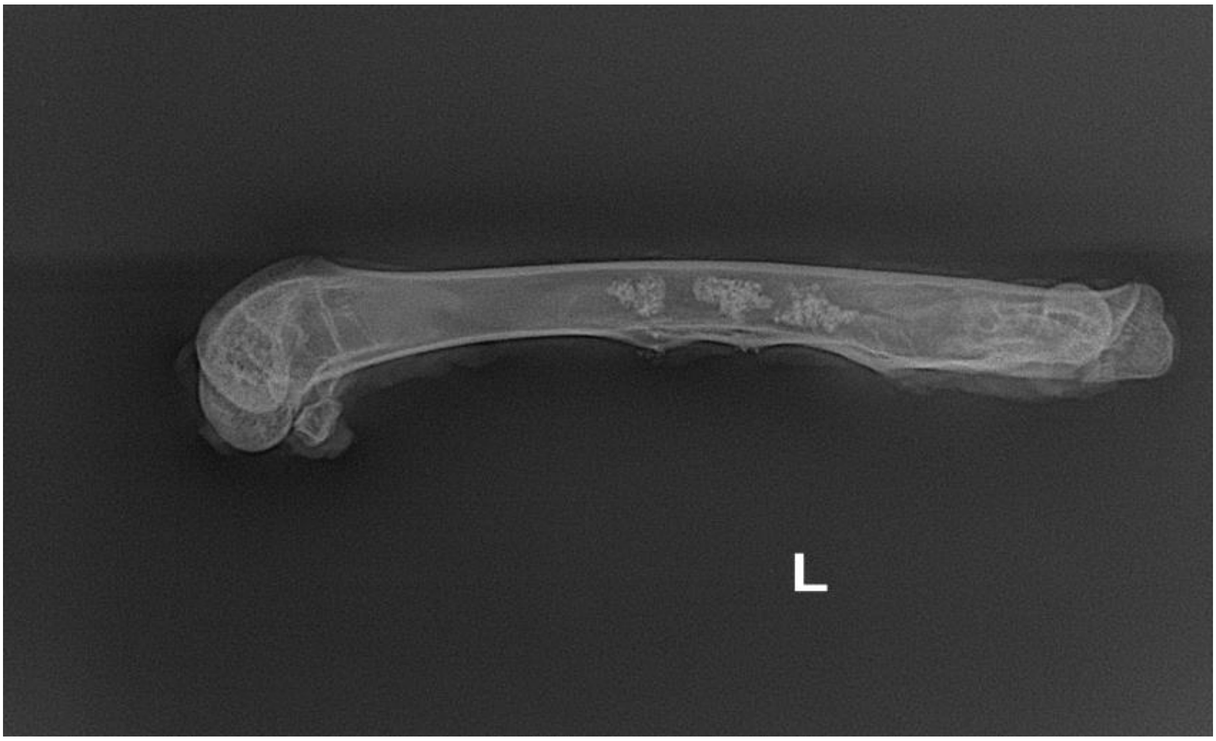

Figure 49: Animal no. 18, left femur

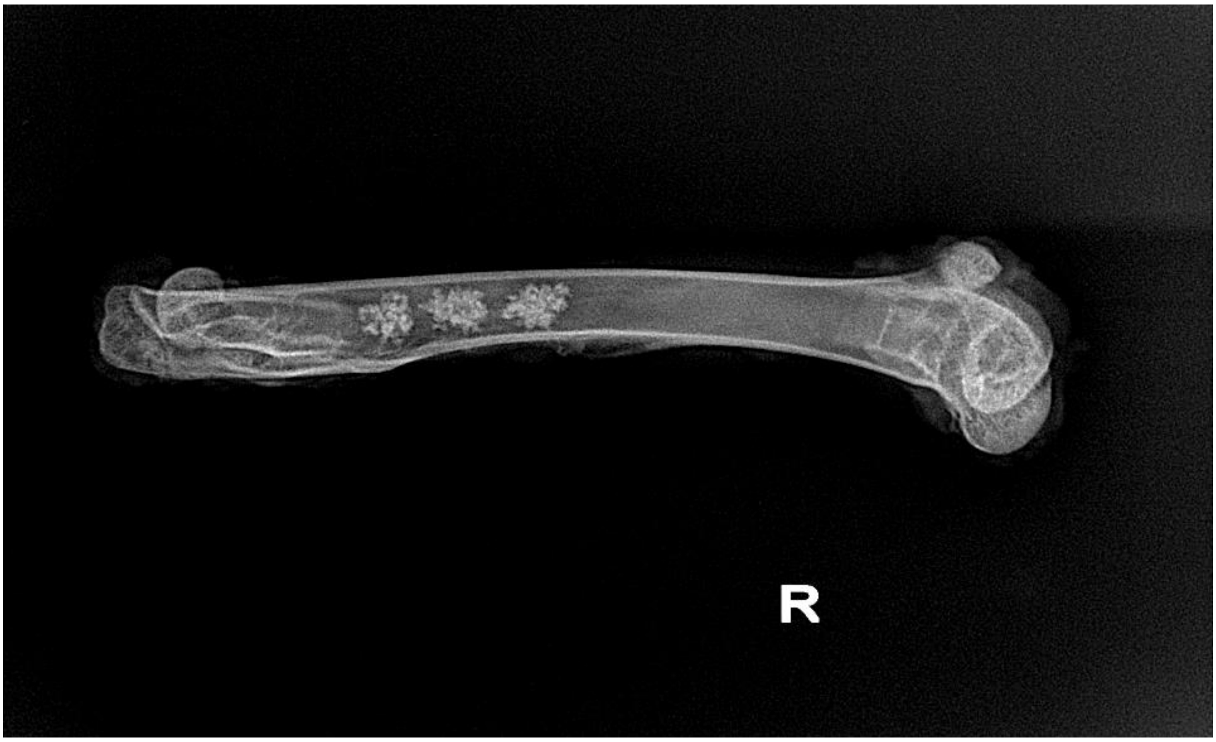

Figure 50: Animal no. 18, right femur

X-Ray photos: Group G2

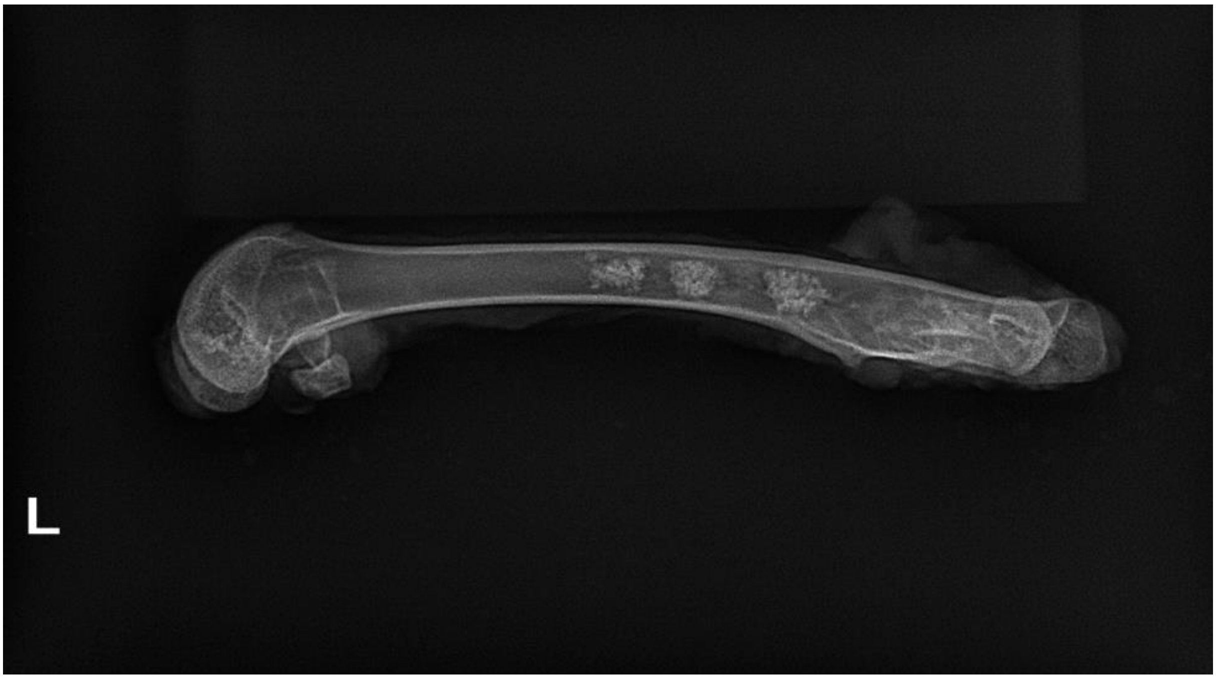

Figure 51: Animal no. 12, left femur

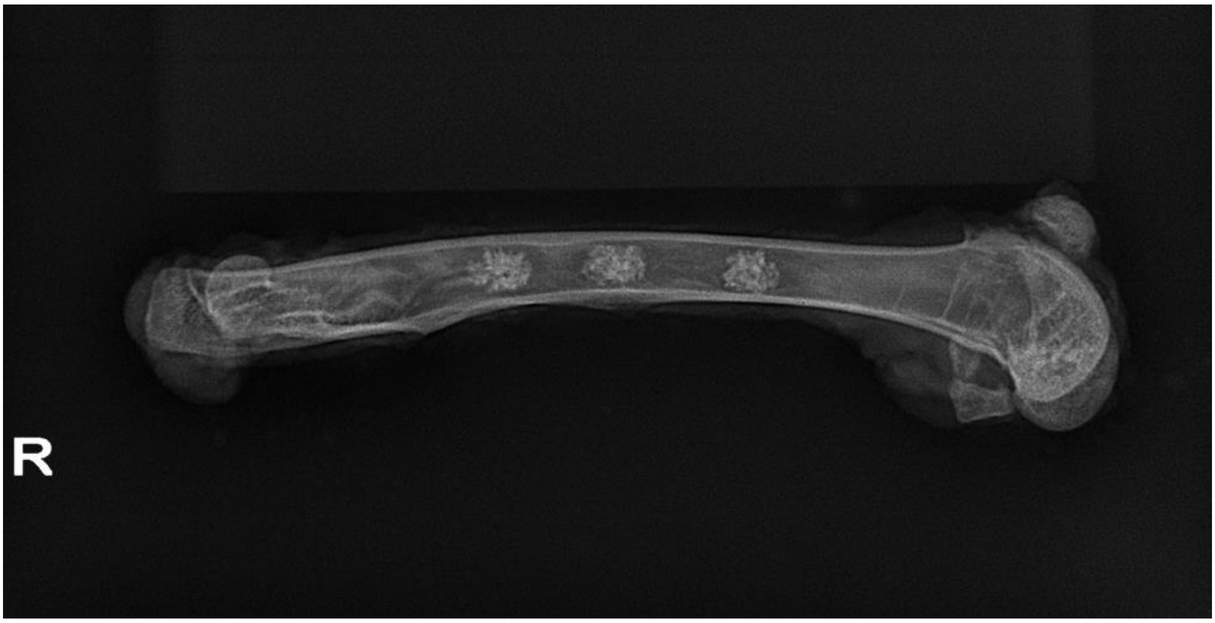

Figure 52: Animal no. 12, right femur

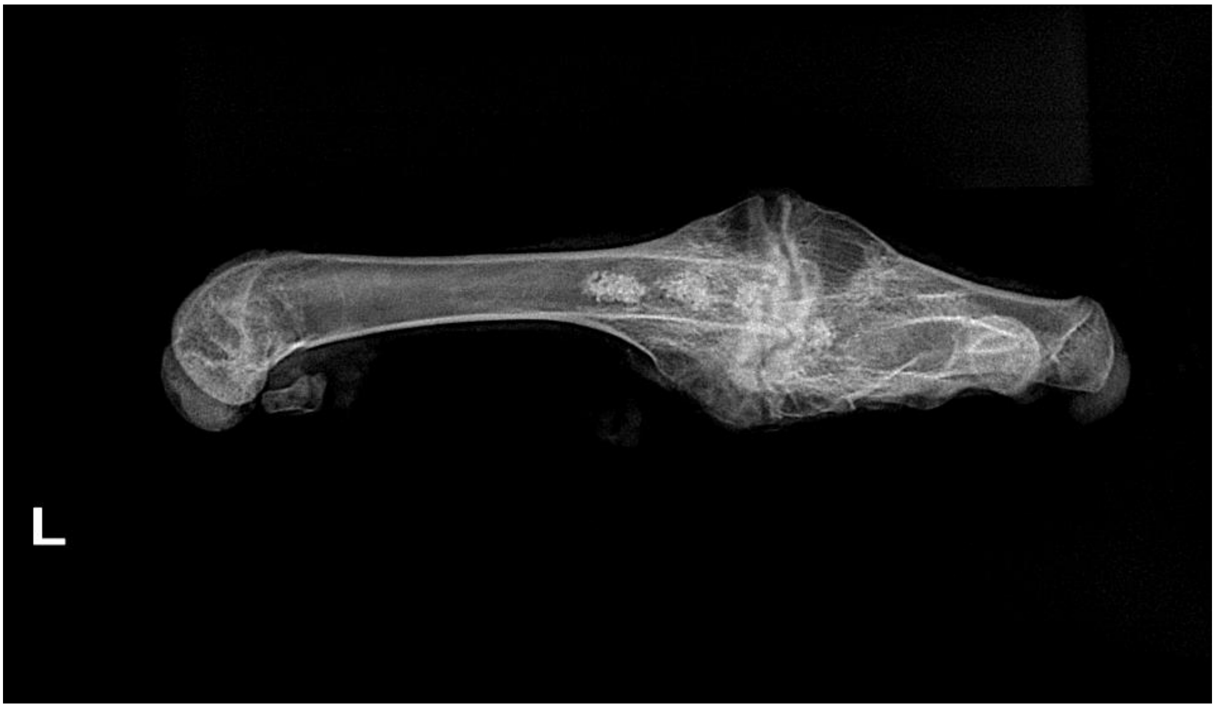

Figure 53: Animal no. 13, left femur

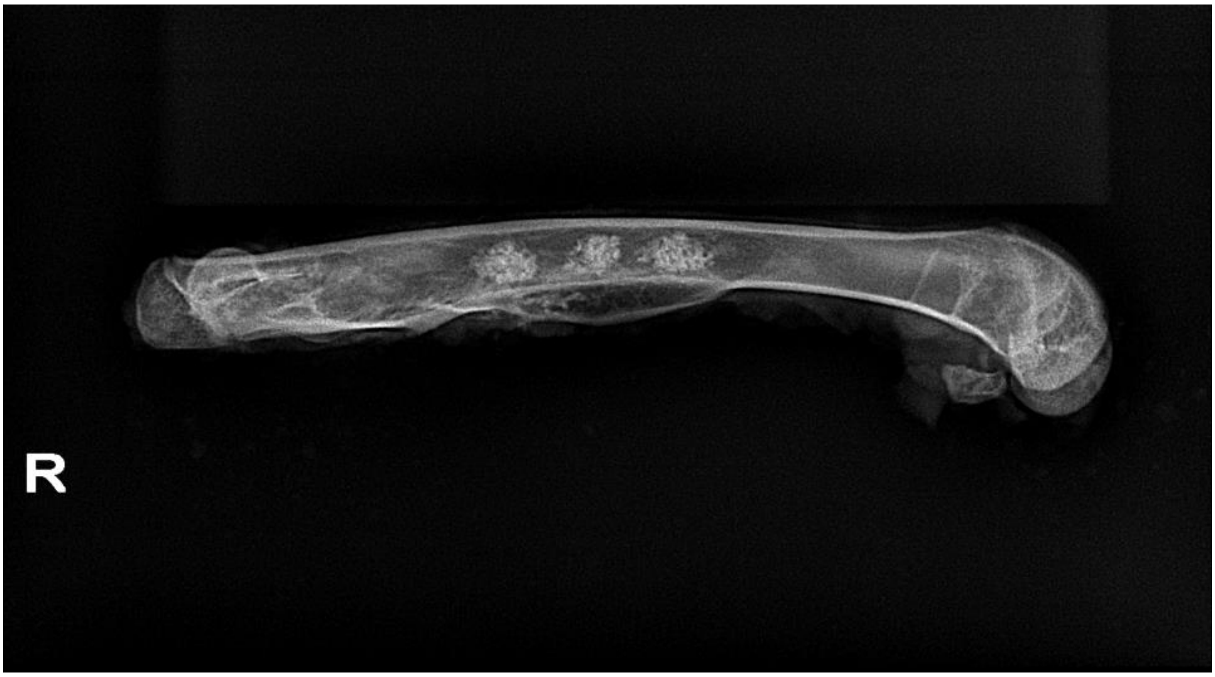

Figure 54: Animal no. 13, right femur

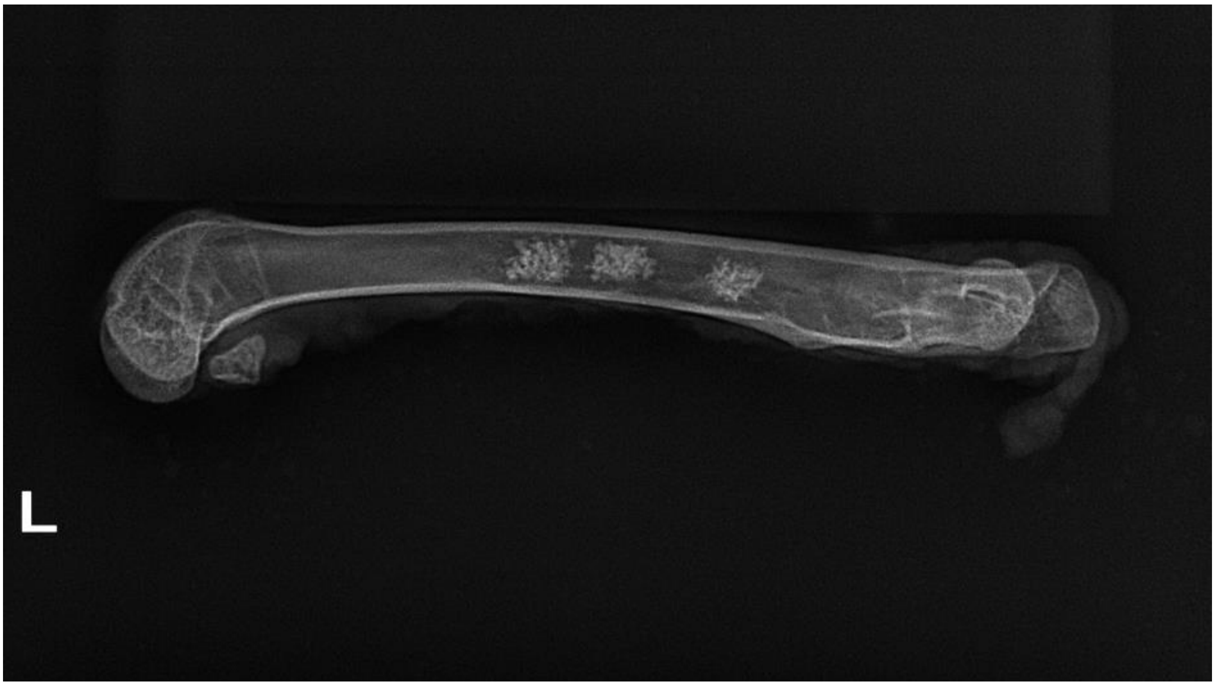

Figure 55: Animal no. 14, left femur

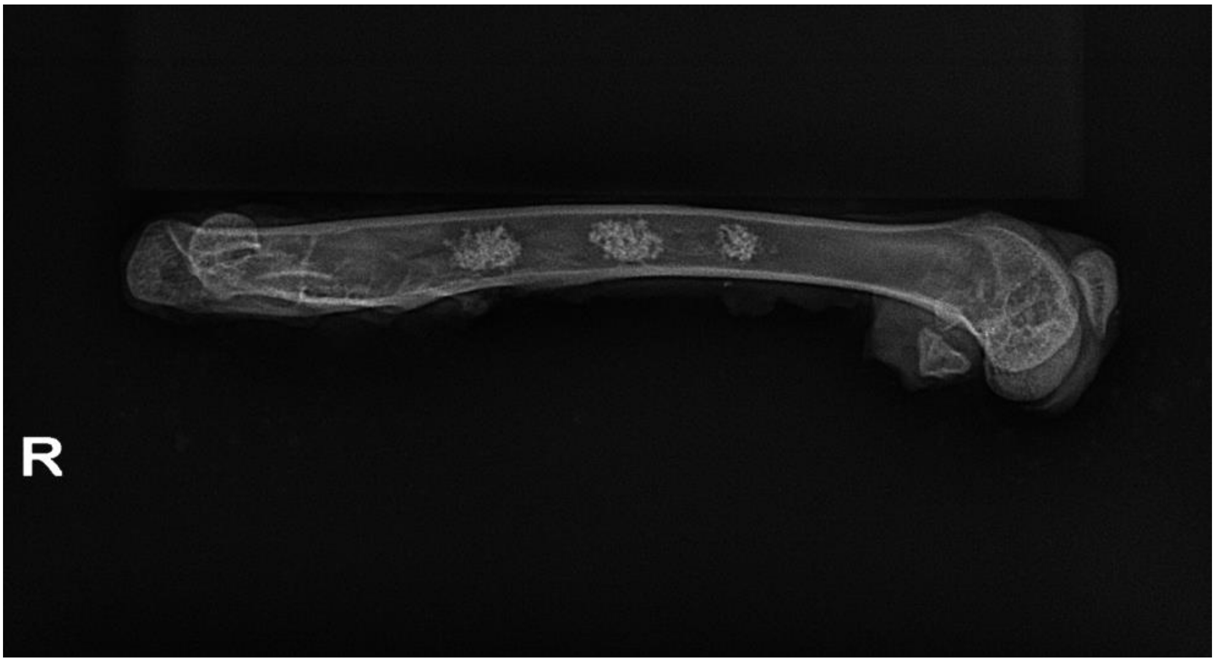

Figure 56: Animal no. 14, right femur

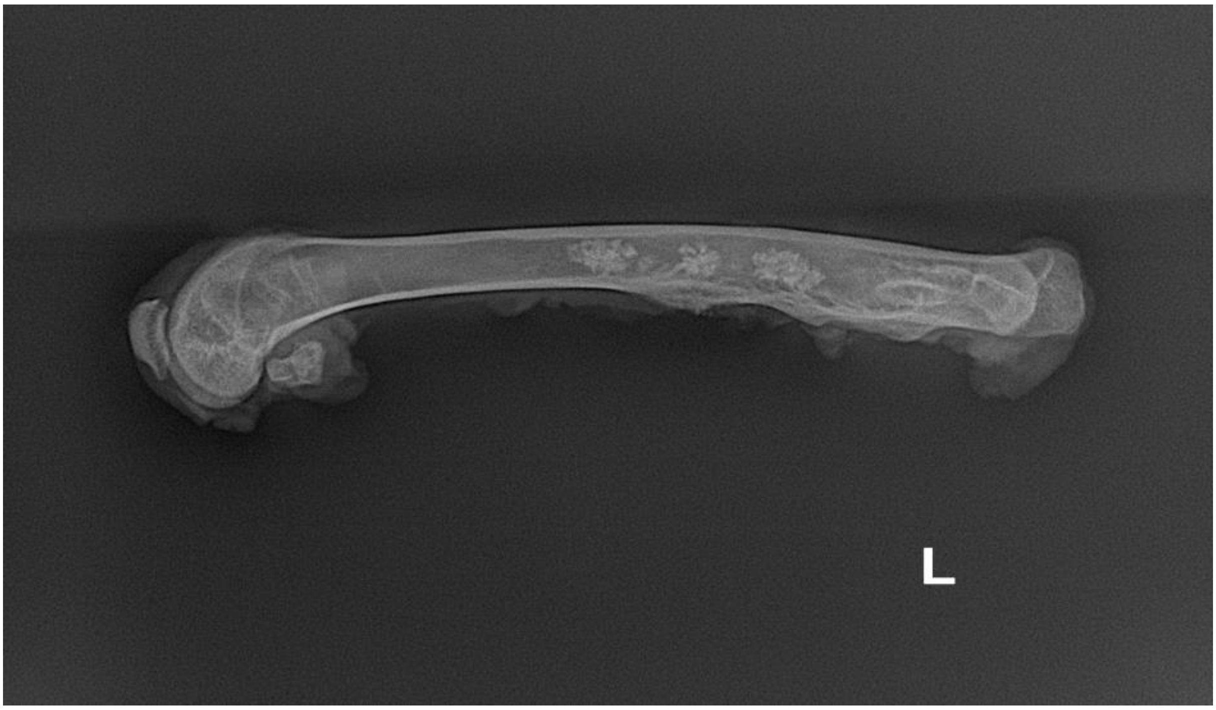

Figure 57: Animal no. 16, left femur

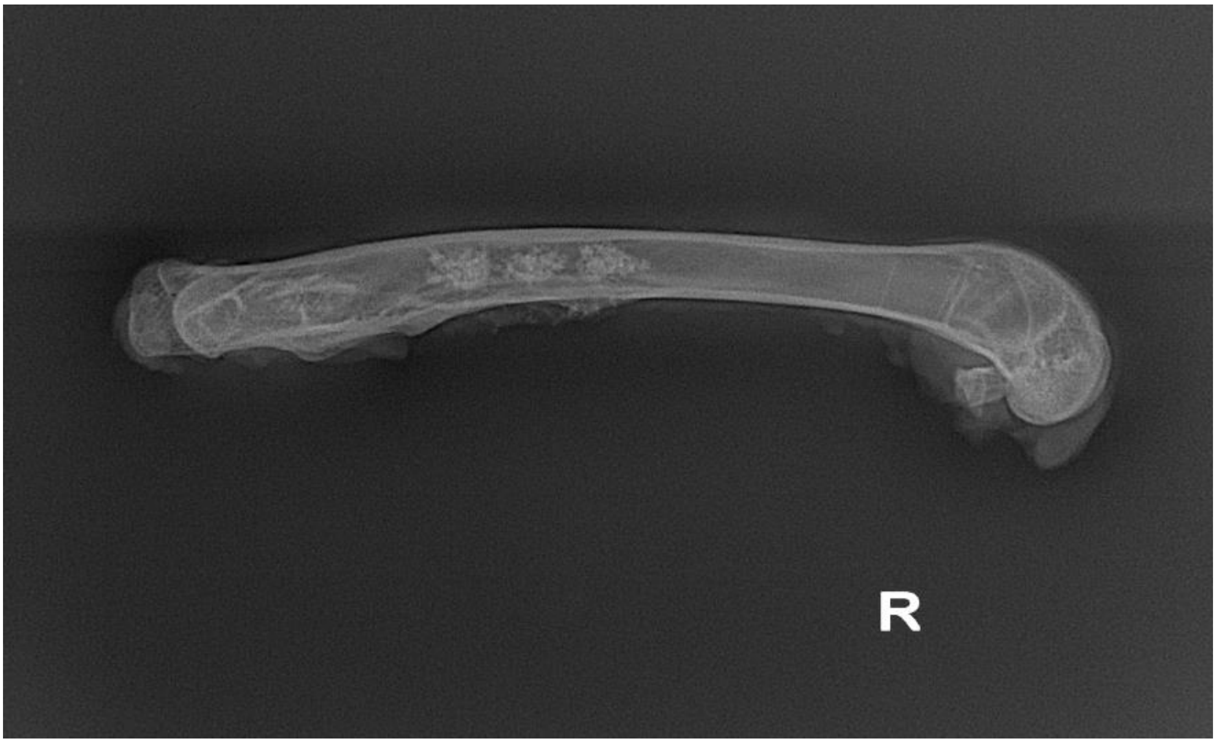

Figure 58: Animal no. 16, right femur

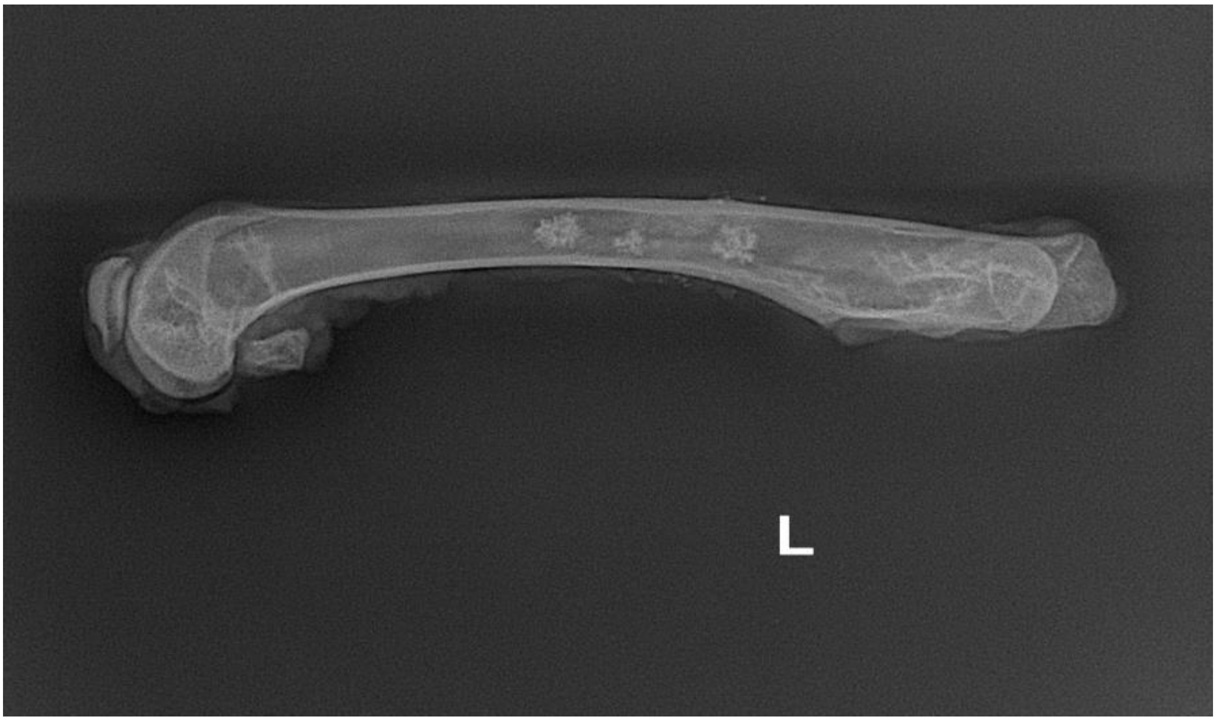

Figure 59: Animal no. 17, left femur

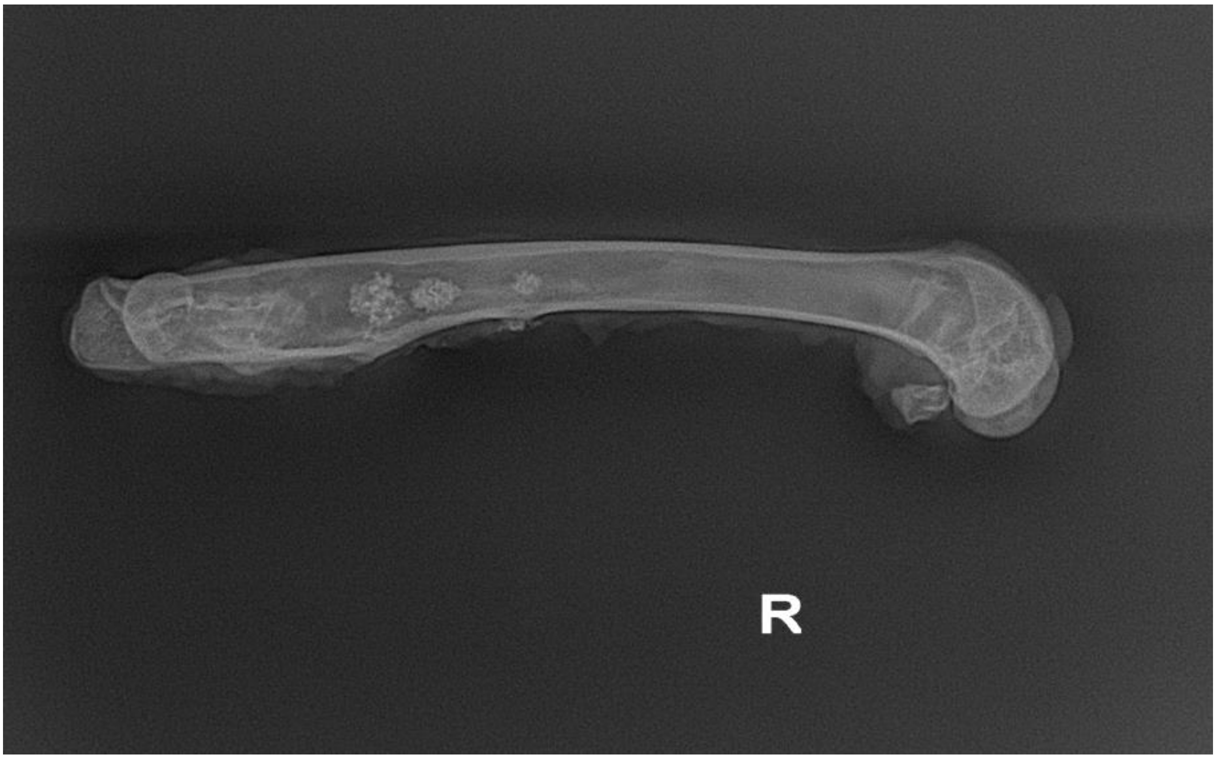

Figure 60: Animal no. 17, right femur
